# Iron‐MOFs for Biomedical Applications

**DOI:** 10.1002/adhm.202402630

**Published:** 2024-10-10

**Authors:** Zhihao Yu, Mathilde Lepoitevin, Christian Serre

**Affiliations:** ^1^ Institut des Matériaux Poreux de Paris ENS ESPCI Paris CNRS PSL University Paris France

**Keywords:** biomedicine, drug delivery, Fe‐based metal–organic frameworks, ferroptosis, porous materials

## Abstract

Over the past two decades, iron‐based metal–organic frameworks (Fe‐MOFs) have attracted significant research interest in biomedicine due to their low toxicity, tunable degradability, substantial drug loading capacity, versatile structures, and multimodal functionalities. Despite their great potential, the transition of Fe‐MOFs–based composites from laboratory research to clinical products remains challenging. This review evaluates the key properties that distinguish Fe‐MOFs from other MOFs and highlights recent advances in synthesis routes, surface engineering, and shaping technologies. In particular, it focuses on their applications in biosensing, antimicrobial, and anticancer therapies. In addition, the review emphasizes the need to develop scalable, environmentally friendly, and cost‐effective production methods for additional Fe‐MOFs to meet the specific requirements of various biomedical applications. Despite the ability of Fe‐MOFs–based composites to combine therapies, significant hurdles still remain, including the need for a deeper understanding of their therapeutic mechanisms and potential risks of resistance and overdose. Systematically addressing these challenges could significantly enhance the prospects of Fe‐MOFs in biomedicine and potentially facilitate their integration into mainstream clinical practice.

## Graphical abstract

This review introduces the unique properties of iron‐based metal–organic frameworks (Fe‐MOFs), the advances in synthesis and formulation, and their state‐of‐the‐art applications in biomedicine. It discusses the challenges in transitioning Fe‐MOFs from research to clinical use, emphasizing the need for eco‐friendly production methods, and a deeper understanding of their therapeutic mechanisms and potential risks.

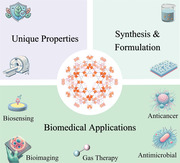

## 1 Introduction

**Figure 1:**
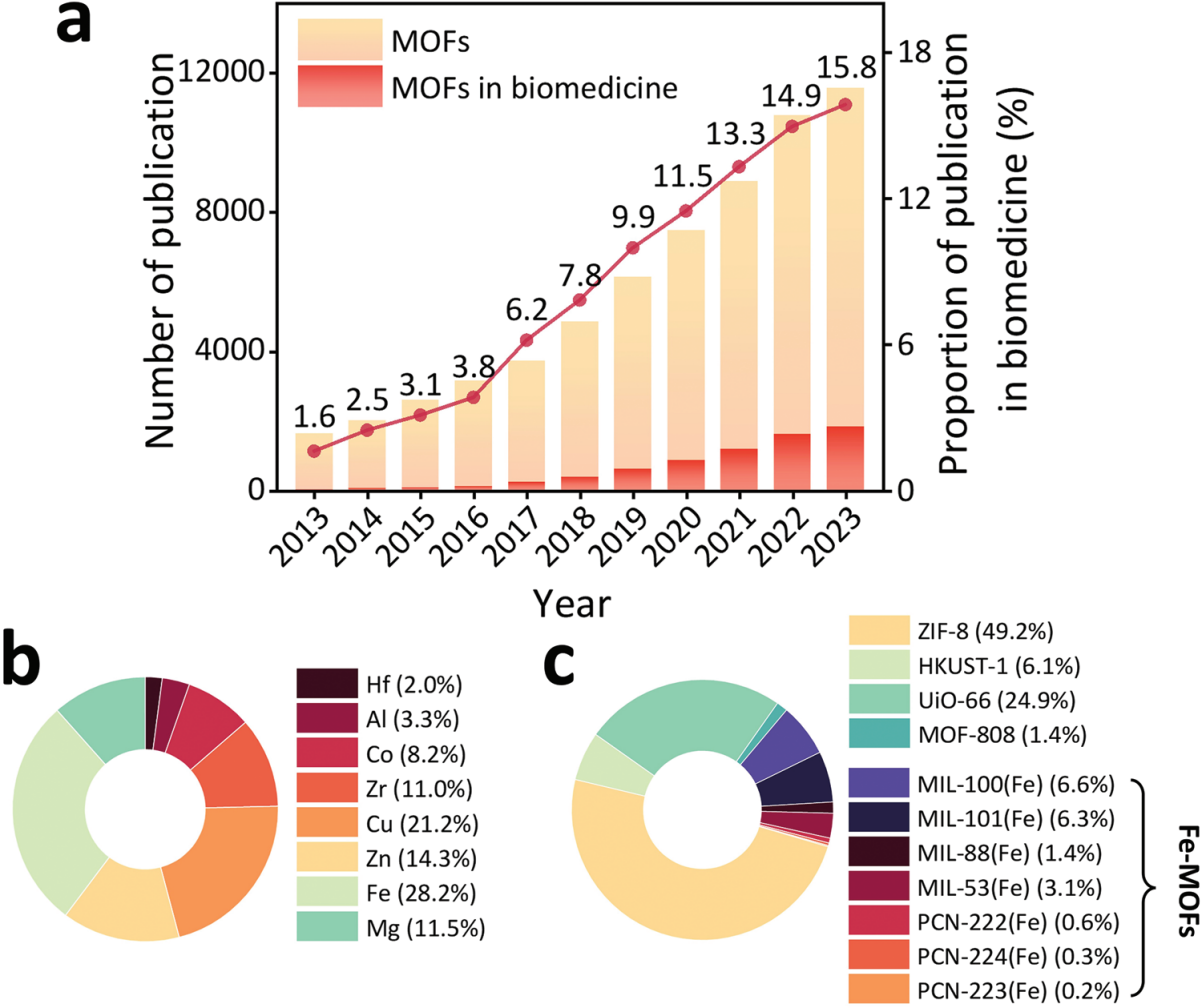
a) The growth in publications of MOFs and MOFs for biomedical use over the past decade, with the rising trend of biomedical MOFs as a proportion of total MOF publications. b) The proportion of different metals used in MOF biomedical research. c) The proportion of common MOFs employed in the biomedical field. The data was achieved through the SCOPUS database, the search string for MOFs was [“metal–organic framework” OR “metal organic framework”]. For MOFs in biomedicine, the search string was [“metal–organic framework” OR “metal organic framework” AND (biomedical OR biomedicine OR bioimaging OR biosensing OR cancer OR tumor OR microbial OR bacteria OR cardiovascular OR wound OR tissue OR bone OR inflammation OR anti‐inflammatory OR disease OR diagnosis OR therapy OR theranostics)]. Specific metals or MOFs were searched by adding their names to the search string of MOFs in biomedicine.

Over the past two decades, metal–organic frameworks (MOFs) have been instrumental in uncovering a multitude of ground‐breaking findings. MOFs, a class of organic–inorganic hybrid nanomaterials synthesized from metal sub‐units (clusters, chains, and layers) and organic linkers (carboxylates, azolates, phosphonates, etc), exhibit a remarkable diversity in terms of composition, pore size/shape.^[^ ^1^, ^2^, ^3^ ^]^ This makes them ideal for a wide range of applications, including gas storage and separation, heat reallocation, catalysis, energy conversion, and storage, as well as sensing, diagnosis, therapy, and theranostics. In particular, MOFs to capture CO_2_ in air are now commercially available while other industrial applications of MOFs, such as gas purification, dehumidification, or catalysis, are under development.^[^ ^4^, ^5^, ^6^ ^]^ In addition, the customizable architecture of biocompatible MOFs exhibit potential applications in biosensing, bioimaging, and dynamic drug delivery systems (DDS), particularly for antimicrobial and antitumor therapies.^[^ ^7^, ^8^, ^9^, ^10^ ^]^ However, it is easy to imagine that the requirements for biomedical applications, especially clinical ones, are much more stringent than for other commercial activities. Although much of the work on biological applications of MOFs is still in its early stages and presents challenges, the proportion of MOF publications in this field has increased tenfold over this period, from 1.6% to 16% (**Figure** **1**a).^[^ ^11^ ^]^ Chemists are increasingly seeking collaborations with biologists and clinicians to guide their research efforts.

In 2023, MOFs have progressed from laboratory research to clinical trials.^[^ ^12^ ^]^ Wenbin Lin's team at the University of Chicago has developed RiMO‐301,^[^ ^12^ ^]^ the first MOF to enter human clinical trials. Early clinical results support the use of RiMO‐301 as a potential radiotherapy enhancer and are prompting further Phase II trials to explore its use in patients undergoing radiotherapy.^[^ ^12^ ^]^ In addition, Russell Morris's team (University of St Andrews, UK) has embedded NO‐loaded MOFs in bandages to promote wound healing and coating stents and catheters with the material to prevent thrombosis and reduce the risk of infection, marking the first clinical trials of MOFs on human skin.^[^ ^13^, ^14^ ^]^

Iron is the most utilized metal in MOFs for biomedical applications (Figure 1b). Several representative Fe‐MOFs (Figure 1c) have been reported and studied due to their low toxicity, stability, biodegradability, high drug‐loading capacity, and versatile structure. Despite the extensive research conducted, the transition of Fe‐MOFs into clinical products remains an unfulfilled objective. Recent review articles have generally explored Fe‐MOFs' therapeutic principles, classifications, and advances in biomedical applications, but few have adequately highlighted the unique advantages of Fe‐MOFs compared to other MOFs.^[^ ^15^, ^16^, ^17^, ^18^ ^]^ It is noteworthy that the review by Kudarha et al. summarized the mechanisms of Fe‐MOF–based ferroptosis in cancer therapy.^[^ ^19^ ^]^ However, it lacks a detailed discussion on the synthesis methods, structural features of Fe‐MOFs, and more possible bio‐applications such as antimicrobial therapies. Moreover, existing reviews frequently fail to incorporate the latest developments in environmentally friendly and cost‐effective synthesis methods. Additionally, there is a need for greater emphasis on the toxicity, degradability, and biocompatibility of Fe‐MOFs, as well as the challenges in translating laboratory findings into clinical applications. For instance, while many current reviews focus on the multifunctional character of Fe‐MOFs in the design of multimodal therapeutic systems, a significant drawback of certain hybrid systems is their occasional complexity, whether in terms of production or understanding of therapeutic mechanism. These challenges will be highlighted for the first time in this review. Overall, this paper will systematically discuss the toxicity, degradability, and unique properties of Fe‐MOFs as drug carriers and ferroptosis inducer, then will outline achievements for scalable, green synthesis, as well as the technologies for surface engineering and shaping of Fe‐MOFs. Then, it will introduce recent advances in the use of Fe‐MOFs for biosensing, anticancer, and antimicrobial research. Finally, it will discuss the potential and future directions of Fe‐MOFs as viable clinical options.

## 2 Fe‐MOFs in Biomedical Applications

### 2.1 Key Properties of MOFs for Biomedical Applications

#### 2.1.1 Toxicity

**Figure 2:**
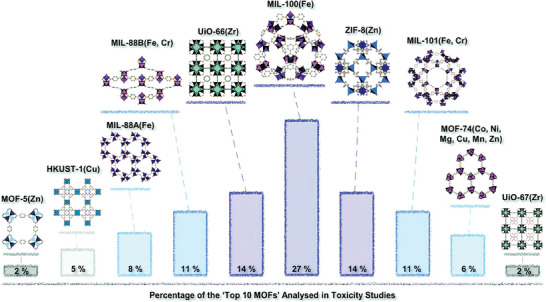
Overview of the percentages of the “Top 10 MOFs” analyzed in toxicity studies. Reproduced with permission.^[^ ^21^ ^]^ Copyright 2022, Royal Society of Chemistry.

When designing MOFs for biomedical applications, toxicity, and degradability are the primary factors to consider.^[^ ^20^ ^]^ A particle size typically below 200 nm is favored to facilitate capillary circulation. However, smaller dimensions also increase the potential for nanotoxicity, which can disrupt physiological functions, leading to tissue inflammation, alteration of redox balance, or cell death.^[^ ^21^ ^]^ The composition of nanoMOFs strongly influences their toxicity. MOFs consist of metal clusters and organic linkers. The toxicity of the metals depends on their specific type, oxidation state, and dosage. Ettlinger et al. have conducted a comprehensive review and roughly ranked MOFs containing different metals in terms of their cytotoxic effect.^[^ ^21^ ^]^ Their findings indicate that the least toxic MOFs contain metals such as Ca, Bi, and Eu. These are followed, in ascending order of toxicity, by MOFs containing Ti, Fe, Co, Al, and Cr. MOFs containing Zr, Mg, Gd, Ni, and Zn exhibit moderate toxicity, while those containing Cu and Mn are the most toxic. Among the “top ten MOFs” analyzed for toxicity (which include MIL‐100, UiO‐66, ZIF‐8, MIL‐88B, MIL‐101, MIL‐88A, MOF‐74, HKUST‐1, MOF‐5, and UiO‐67), Fe‐based MOFs are the most prevalent, indicating a significant research effort in the development of Fe‐MOFs for biomedical applications (**Figure** **2**).^[^ ^21^ ^]^ A commonly observed mechanism of toxicity caused by metals (e.g., Ag, Cu, Fe, and Cd) with redox activity is the generation of reactive oxygen species or reactive nitrogen species, such as hydroxyl radicals (·OH), superoxide anions (·O_2_^−^), hydrogen peroxide (H_2_O_2_), or nitric oxide (NO).^[^ ^22^, ^23^ ^]^ These reactive substances can damage cell membranes, DNA, and proteins, ultimately leading to cell death. Besides, some metal ions (e.g., Ga, Fe, Zn, and Cd) can disrupt the normal biological ion balance, interfere with intracellular physiological processes and thus induce toxicity.^[^ ^24^, ^25^ ^]^ In addition, some metal ions (e.g., Pb, Mn, Ag, and Co) can bind to intracellular proteins to form precipitates or insoluble complexes. These precipitates can accumulate in tissues or organs, impairing structural integrity and function and potentially triggering toxic responses.^[^ ^26^, ^27^, ^28^ ^]^

The toxicity of organic linkers is primarily due to their ability to cross membrane barriers, which can lead to inflammatory and other adverse effects. Usually carboxylates are not much toxic due to their deprotonation in body fluids conditions which drastically enhance their solubility and thus clearance; on the contrary azolates, particularly pyrazolates, are more toxic.^[^ ^29^ ^]^ In addition, the surface chemistry of MOFs has a marked effect on toxicity; positively charged surfaces are considered more hazardous to cells because the negative charge of the cellular lipid bilayer can facilitate their interaction and uptake.^[^ ^30^ ^]^ The influence of the shape or topology of MOF nanoparticles on toxicity remains under‐researched. Tamames–Tabar et al. investigated structurally similar Fe‐based MOFs suggests that topology may have only a minor influence on their toxicity.^[^ ^31^ ^]^ In addition, another study by some of us have reported the impact of defects in MIL‐100(Fe) as a determinant parameter of toxicity.^[^ ^32^ ^]^ The defect content consequently influences their chemical stability, inherent toxicity, drug loading efficiency, and drug release kinetics.^[^ ^32^ ^]^

In addition, any solvents or additives that may remain within the structure or pores of the MOF nanoparticles following the synthesis process must also be considered as a potential source of toxicity and one shall privilege green synthesis protocols.

#### 2.1.2 Degradability

Another crucial aspect to consider when designing MOFs for biomedical applications is their degradability in biological media. MOFs for in vivo therapeutics are mostly nanoscale, but a reduction of the particle size can also negatively impact the stability of MOF nanoparticles in biological media. Smaller particles expose a greater surface area to their microenvironment, allowing for faster diffusion into the particles, which often results in more rapid degradation of the MOF nanoparticles.

The stability of MOFs can be predicted using the hard and soft acids and bases (HSAB) principle.^[^ ^33^, ^34^ ^]^The HSAB principle indicates that stable MOFs are frequently constructed with carboxylate‐based ligands (considered as hard Lewis bases) and high‐valence metal ions (considered as hard Lewis acids). Conversely, azolate‐based ligands (considered as soft Lewis bases) and low‐valence transition metal ions (considered as soft Lewis acids) can also form relatively strong coordination bonds. Consequently, numerous high‐valence metal ions, including Fe^3+^, Cr^3+^, and Zr^4+^, have been utilized in conjunction with carboxylate ligands to generate water‐stable MOFs. While high‐valent cations influence the a priori stability of the resulting MOFs, it is important to consider other factors that may affect their degradability and stability. The factors include the nature and nuclearity of the metal clusters, the strength of the coordination bonds with ligands, the nature and number of chelating functions, the presence of open metal sites and/or defects, and the MOF's hydrophilic/hydrophobic balance and pore size.

Furthermore, the physicochemical properties (i.e., charge and surface properties) of MOFs also affect their colloidal and chemical stability in biological fluids.^[^ ^35^, ^36^ ^]^ The surface charge of MOFs can also influence both internal and external electrostatic interactions with biological macromolecules, affecting their dispersion and potential aggregation in body fluids. Surface chemistry, particularly the presence of functional groups, can impact hydrophilic or hydrophobic characteristics to MOFs. Those with hydrophilic surfaces may exhibit better stability in aqueous biological fluids due to reduced aggregation tendencies. In contrast, hydrophobic MOFs might interact more with serum proteins, potentially leading to rapid clearance from the bloodstream and decreased stability.

Studying the toxicity and degradability of MOF nanoparticles is highly relevant. Typically, the faster the degradation, the higher the release of metals and organic linkers, which may potentially increase toxicity. Therefore, it is recommended that the final MOF nanoparticles be composed solely of low‐toxicity units to minimize overall toxicity. It is important to note that MOF nanoparticles may not decompose into their original components. Alternatively, they may undergo further crystallization or amorphization, or the formation of other degradation products or metabolites, such as metal oxides or phosphates that are poorly soluble which might prevent from immediate toxicity.^[^ ^37^ ^]^

#### 2.1.3 Drug Delivery Systems

**Figure 3:**
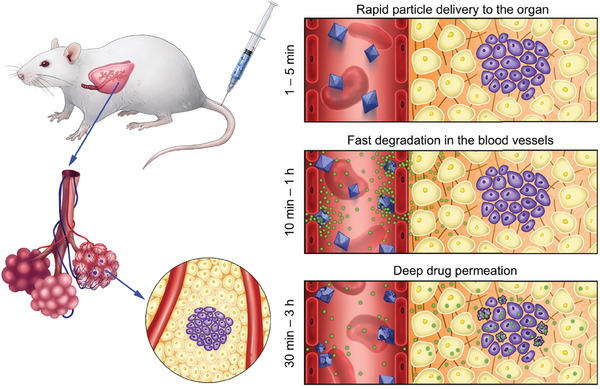
Left panel: Drug formulation is systematically administered to a mouse and sequestered in capillaries of metastatic lung tissue (tumor cells shown in purple). Right panel: MOFs rapidly enter pulmonary capillaries, anchor to the endothelium, and begin to release drugs (shown in green) while they undergo structural decomposition. The released drug crosses the vessel walls, reaches the metastatic node by gradient‐driven diffusion and damages cancer cells. Reproduced with permission.^[^ ^39^ ^]^ Copyright 2022, Nature.

MOFs have emerged as a key class of materials in the development of advanced DDS. Their intrinsic porosity, coupled with the ease of functionalization, paves the way for the encapsulation of a wide range of bioactive agents. In anticancer research, MOF‐based DDS are currently delivered to tumors, primarily via the enhanced permeability and retention (EPR) effect. This effect is based on the distinctive vascular architecture of tumors, which includes poorly aligned and irregular tumor vessels with wide fenestrations. These abnormalities allow nanoparticles, which are typically too large to pass through the tight junctions of normal blood vessels, to more easily enter tumor tissue. Once inside the tumor interstitium, the poor lymphatic drainage systems of tumors prevent these particles from being efficiently cleared, allowing them to accumulate more in tumor tissue than in normal tissue. However, recent reports have showed that active transport and retention (ATR) mechanisms are also involved in the tumor uptake and retention of nanoparticles, as observed in gold and ferritin nanoparticles.^[^ ^38^ ^]^ The ATR effect of MOF nanoparticles has yet to be discovered, necessitating further studies to elucidate the balance between passive and active extravasation pathways for MOFs. In the context of cancer treatment using the EPR effect, MOFs can be designed to encapsulate therapeutic agents and protect them from premature degradation while circulating in the bloodstream. Once these MOF‐based nanocarriers accumulate in tumor tissue due to the EPR effect, the drugs can be released in a controlled manner, either through degradation of the MOF structure or through stimuli‐responsive mechanisms that respond to the unique environment within tumors (such as lower pH or higher levels of certain enzymes, H_2_O_2_, or glutathione). However, reliance on the EPR effect should be used with caution, as its efficacy may not be as significant as previously thought. Tumors often develop dense extracellular matrices that can physically impede nanoparticle penetration despite the presence of leaky vessels. In addition, the size, shape, and surface properties of MOFs can affect their ability to extravasate and their interactions with the tumor microenvironment (TME), potentially limiting the uniformity and efficiency of drug delivery. In addition to physiological barriers, the rapid clearance of nanoparticles by the mononuclear phagocyte system reduces the amount of drug that can accumulate in tumor tissue, thereby diminishing the potential benefits of the EPR effect. Based on the knowledge of the EPR effect and a fragile mesoporous iron MOF, MIL‐101(Fe), Zelepukin et al. proposed a new anticancer drug delivery concept termed flash release in endothelium (FlaRE, **Figure** **3**), and the efficacy of the FlaRE approach has been experimentally demonstrated in the treatment of metastatic tumors in the lung.^[^ ^39^ ^]^ FlaRE utilizes rapid drug release kinetics, with MIL‐101(Fe) degrading in as fast as 44 s. This immediate degradation allows for rapid drug release into blood vessels, creating a high concentration gradient that promotes diffusion across capillary walls into the surrounding interstitial space. However, the approach could result in unpredictable drug distribution if nanoparticles fail to adhere to target sites, potentially affecting non‐tumor tissues. Moreover, rapid release could limit the therapeutic window, requiring precise control, and timing to maintain effective drug concentrations. Although theoretically promising, FlaRE is still in the proof‐of‐concept stage, requiring extensive clinical validation to ensure its safety and efficacy.

Besides antitumor treatment, MOFs have been successfully used to load phototherapeutic agents or antibiotics, providing a promising avenue for sustained antimicrobial therapy.^[^ ^40^ ^]^ This can be valuable in addressing the ongoing challenge of bacterial resistance by reducing the use or increasing the efficacy of antibiotics. In addition, MOFs have shown great potential for encapsulating bioimaging agents such as fluorescent molecules and radiotracers, setting the stage for innovative approaches to bioimaging‐assisted treatment.^[^ ^41^ ^]^

MOFs, with their highly customizable features such as sizes, pore structures, shapes, and surface properties, offer significant potential for tailoring DDS to meet specific therapeutic needs. These materials can be engineered to possess stimuli‐responsive degradation characteristics, enabling drug release in response to environmental triggers such as pH, temperature, or enzymatic activity prevalent in the target tissue or cellular environment. Combined with predictions of MOF toxicity, this enables the design of DDS that is safer and more compatible with human physiology. **Table** **1** summarizes the key factors influencing the primary properties of MOFs for biomedical applications, and their optimization strategy.

**Table 1:** Key factors influencing the primary properties of MOFs for biomedical applications.

Category	Key Factor	Optimization
Toxicity	Size	Smaller particles (<200 nm) facilitate capillary circulation but increase nanotoxicity. Balance size to minimize nanotoxicity while ensuring effective circulation.
	Metal Clusters	Select metals with lower cytotoxicity (e.g., Ca, Bi, Eu, Ti, and Fe) to reduce overall toxicity.
	Organic Linkers	Use linker with higher solubility in body fluids, lower cytotoxicity (e.g., carboxylates) to reduce overall toxicity.
	Defects	Controlling defect levels ensures chemical stability and minimizes toxicity.
	Surface Chemistry	Modify surface charge to reducing interactions with cell membranes could decrease toxicity.
Degradability	Size	Smaller particles degrade faster due to a higher surface‐to‐volume ratio, leading to a more rapid release of metal ions and organic linkers. Optimize the particle size to control degradation rates and ensure balanced therapeutic effects.
	Coordination Bonds	Strong coordination bonds between high‐valence metal ions (e.g., Fe^3+^, Cr^3+^, and Zr^4+^) and carboxylates improve stability.
	Metal Cluster Properties	Properties such as nuclearity and the presence of open metal sites affect chemical stability and degradability. Optimize metal cluster composition to control degradation.
	Surface Properties	Modification to hydrophilic surfaces could reduce aggregation and improve stability in biological fluids.
DDS	Size	MOF particle size affects circulation, tumor accumulation (via EPR effect), and drug loading capacity. Tailor MOF particle size to maximize effective delivery and optimize retention in tumor tissues.
	Stimuli‐responsive Properties	Incorporate stimuli‐responsive mechanisms (such as pH, temperature, and enzymes) for controlled drug release in targeted tissues.
	Degradation Rates	Controlled degradation ensures timed and precise release of therapeutic agents to match the therapeutic window for effective treatment.
	Pore Structures	Engineer pore structures to enhance drug loading and ensure controlled and sustained release.

### 2.2 Fe‐MOFs in Biomedical Applications

#### 2.2.1 Unique Properties and Advantages of Fe‐MOFs

Fe‐MOFs are preferred in biomedical research due to i) the inherent low toxicity of endogenous iron; ii) trivalent iron ions contribute to the inherent stability of the MOFs; iii) their green synthesis routes have been extensively developed in recent years. This has led to iron being the most utilized metal in the study of MOFs for biomedical applications (Figure 1). Figure 1c illustrates the various MOFs that are predominantly investigated for biomedical applications, including seven Fe‐MOFs, one Zn‐MOF (ZIF‐8), one Cu‐MOF (HKUST‐1), and two Zr‐MOFs (UiO‐66 and MOF‐808). In general, Fe‐MOFs display lower toxicity than Zr‐, Zn‐, and Cu‐MOFs, while exhibiting intermediate stability, in water, between the one of the highly stable Zr‐MOFs and the relatively less stable Zn‐ and Cu‐based MOFs. Furthermore, Fe‐MOFs have exhibited remarkable versatility in combining multiple therapeutic functions, such as magnetic resonance imaging (MRI) and catalysis, rendering them optimal candidates for multifunctional platforms in theranostics. Finally, one of the most important features of Fe‐MOFs in biomedicine, is the intrinsic ability of Fe‐MOFs to promote Fenton reaction; this is of a strong interest when developing anticancer or antimicrobial strategies. Note that Cu‐MOFs are also capable of this catalytic activity, but their higher toxicity is a strong limitation to their practical use.

**Table** **2** presents the fundamental information for biomedical applications, including their pore size/structure, particle size, and representative synthesis conditions. The pore size of MOFs is indeed a pivotal factor in the encapsulation of bioactive agents, and considering the particle size and green synthesis of Fe‐MOFs is important for preclinical studies.

**Table 2:** Fundamental information of common Fe‐MOFs for biomedical applications.

Fe‐MOFs	Organic linker	Metal cluster/core	Pore sizes [Å]	Brunauer–Emmett–Teller (BET) surface area [m^2^ g^−1^]	Particle size	Synthesis conditions	Ref.
MIL‐100(Fe)	trimesic acid	Fe_3_(µ_3_‐O)(COO)_6_	25, 29	1900	430 nm	kg scale room temperature synthesis (in water)	[42]
MIL‐100(Fe)	trimesic acid	Fe_3_(µ_3_‐O)(COO)_6_	25, 29	1700	>20 µm	hydrothermal	[43]
MIL‐100(Fe)	trimesic acid	Fe_3_(µ_3_‐O)(COO)_6_	25, 29	1500	<100 nm	microwave‐assisted hydrothermal	[32]
MIL‐101(Fe)	terephthalic acid	Fe_3_(µ_3_‐O)(COO)_6_	29, 34	3200	>1 µm	solvothermal (in N,N‐dimethylformamide (DMF))	[44, 45]
MIL‐101(Fe) ‐NH_2_	2‐amino terephthalic acid	Fe_3_(µ_3_‐O)(COO)_6_	29, 34	3260	200 nm	solvothermal (in DMF)	[44, 46]
MIL‐53(Fe)	terephthalic acid	FeO_4_(OH)_2_	8	12	>1 µm	solvothermal (in DMF)	[47, 48]
MIL‐68(Fe)	terephthalic acid	FeO_4_(OH)_2_	6, 16	400	0.1–1 µm	solvothermal (in DMF)	[49]
MIL‐88A(Fe)	fumaric acid	Fe_3_(µ_3_‐O)(COO)_6_	11		<100 nm	microwave‐assisted hydrothermal	[50]
MIL‐88A(Fe)	fumaric acid	Fe_3_(µ_3_‐O)(COO)_6_	11	26	0.2–1.5 µm	hydrothermal	[50, 51]
MIL‐88B(Fe)	terephthalic acid	Fe_3_(µ_3_‐O)(COO)_6_	11	209	>1 µm	microwave‐assisted solvothermal (in DMF)	[52]
MIL‐89(Fe)	*trans, trans*‐muconic acid	Fe_3_(µ_3_‐O)(COO)_6_	9–16		<150 nm	solvothermal (in EtOH)	[53, 54]
MIL‐127(Fe)	3,3′,5,5′‐azobenzenetetracarboxylate	Fe_3_(µ_3_‐O)(COO)_6_	6, 10	1360	250–310 nm	microwave‐assisted solvothermal (in propan‐2‐ol)	[55]
MIL‐127(Fe)	3,3′,5,5′‐azobenzenetetracarboxylate	Fe_3_(µ_3_‐O)(COO)_6_	6, 10	1350	>1 µm	Ambient pressure (in water/propan‐2‐ol)	[55]

Since 2010, some of us have methodically evaluated the cytotoxicity of different iron‐based carboxylate MOFs.^[^ ^31^, ^56^, ^57^, ^58^ ^]^ In 2013, they pioneered the preclinical study of the degradation and elimination mechanisms of three iron‐based carboxylate nanoMOFs (MIL‐88A, MIL‐100, and MIL‐88B_4CH_3_), demonstrating that these nanoparticles exhibited low acute toxicity when injected intravenously into rats.^[^ ^57^ ^]^ They were rapidly absorbed by the liver and spleen, degraded into their constituents and directly excreted without affecting iron homeostasis or liver function, indicating their promise for safe biomedical applications. Al‐Ansari et al. investigated the toxicity of MIL‐89(Fe) using a zebrafish embryo model, demonstrating that low concentrations (<30 µm) of nanoparticles had no significant impact on neuromuscular function, cardiac health, or hepatic lipid metabolism.^[^ ^54^ ^]^

**Figure 4:**
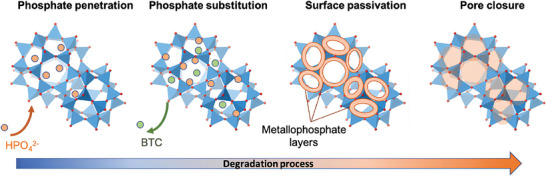
Schematic illustrations of successive steps occurring during the MIL‐100(Fe) degradation process in PBS. Reproduced with permission.^[^ ^62^ ^]^ Copyright 2024, ACS.

The stability of Fe‐MOFs is advantageous for biosensing applications based on in vitro studies. The stable coordination bond between Fe and O allows some Fe‐MOF–based sensors to function even at high temperatures.^[^ ^59^ ^]^ However, when considering the in vivo applications, these materials are confronted with challenges in maintaining their stability. The degradation mechanisms of Fe‐MOFs triggered by blood and their compositional changes are becoming increasingly elucidated. Blood contains over two thousand proteins, various ions, and molecules, including phosphates. Gref et al. first explain the degradation mechanisms of MIL‐100(Fe) in phosphate buffer solution (PBS).^[^ ^60^ ^]^ Upon exposure to phosphate‐containing media, Fe‐MOFs exhibited rapid degradation (**Figure** **4**), with the original ligands being progressively replaced by phosphates within a few hours.^[^ ^61^ ^]^ Christodoulou et al. incubated MIL‐100(Fe) nanoMOFs in serum and blood and utilized atomic precision imaging through scanning electron microscopy with high annular dark field in conjunction with X‐Ray energy‐dispersive spectrometry to analyze elemental changes before and after degradation.^[^ ^37^ ^]^ The results indicated that despite the formation of surface pores, the morphology of nanoMOFs remained essentially unchanged after 2 days in biological fluids (blood and serum). However, there was a significant alteration in the composition of nanoMOFs depending on the degrading medium. Larger proteins, larger than the MOFs' windows, were found to adsorb on the nanoMOFs' exterior, while ions from the suspension medium diffused through the nanoMOFs' pores. These studies demonstrate that nano Fe‐MOFs interact with phosphates and proteins in the blood and that these interactions are rapid. Such degradation is also strongly particle size dependent with large particles degrading much slower than small nanoparticles as the metal oxide/phosphate corona slowdowns the degradation of the Fe‐MOFs. Note that the Fe‐MOFs degrade faster at the blood pH (7.4) than the more acidic parts of the cells or for cutaneous applications (pH ≈ 5).

#### 2.2.2 Fe‐MOFs as MRI Contrast Agents

The paramagnetic nature of Fe^3+^ enables Fe‐MOFs to serve as T2‐weighted MRI contrast agents. Iron‐based MRI contrast agents can cause an increase in T2 relaxation rates, leading to negative image enhancement and thus dark contrast effects in MRI images. Multiple studies have validated Fe‐based carboxylate MOFs as strong T2‐weighted MRI contrast agents.^[^ ^56^, ^63^ ^]^In 2010, Horcajada et al. pioneered the use of iron‐based MOFs as MRI contrast agents.^[^ ^56^ ^]^ The results demonstrated that MIL‐100(Fe) and MIL‐88A(Fe) provided contrast in T2 MRI scans. Furthermore, a polyethylene glycol (PEG) coating may enhance the relaxivity of nanoparticles in two opposing ways, thereby improving imaging capabilities. This is achieved by increasing the size of individual nanoparticles and by reducing their aggregation. However, due to these Fe‐MOFs' inability to provide contrast in T1 MRI scans and their susceptibility to disruption by local metal deposition, calcification, or hemorrhage, their practicality is constrained.^[^ ^64^ ^]^ Recently, some of us developed novel nanocomposites, ultra‐small superparamagnetic iron oxide@MIL‐100(Fe), which demonstrated superparamagnetic properties.^[^ ^65^ ^]^ This has the potential to be an effective in vivo T2 MRI contrast agent.

#### 2.2.3 Ferroptosis as an Anticancer Strategy

Ferroptosis is an iron‐dependent form of programmed cell death driven by iron overload and the accumulation of lethal lipid peroxidation.^[^ ^66^, ^67^, ^68^ ^]^ The term ferroptosis was first introduced in 2012 by Dixon et al. to describe a form of cell death induced by the small molecule erastin, which inhibits the import of cystine.^[^ ^67^ ^]^ Unlike other forms of cell death, ferroptosis is dependent on intracellular iron and triggers distinct biological pathways, representing a significant discovery for cancer therapy. Several ferroptosis inducers, such as the GPX4 inhibitors RSL3 and erastin, have been identified and can effectively regulate ferroptosis. Due to its ability to efficiently induce cellular oxidative damage, ferroptosis has been widely used in cancer treatment.^[^ ^69^, ^70^, ^71^ ^]^

**Figure 5:**
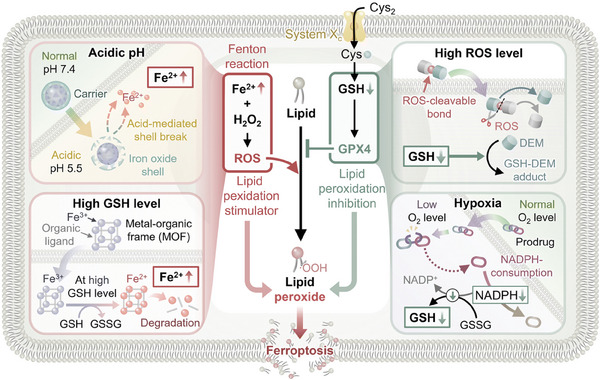
Schematic illustrations of various tumor microenvironment characteristics that can be used for endogenous stimuli‐responsive ferroptosis cancer therapy. Reproduced with permission.^[^ ^72^ ^]^ Copyright 2023, Royal Society of Chemistry.

A hallmark of the tumor microenvironment includes acidic pH, hypoxia, and elevated levels of glutathione (GSH) and ROS. These characteristics can be exploited through interactions with externally applied nanocarriers, thereby facilitating stimuli‐responsive delivery of cancer therapeutics. Enhancing iron levels or reducing glutathione content within cancer cells promotes ferroptosis, advancing the efficacy of targeted treatments (**Figure** **5**).^[^ ^72^ ^]^

GSH is a major endogenous antioxidant in mammalian tissues that directly neutralizes free radicals and ROS while maintaining the reduced form of exogenous antioxidants such as vitamins C and E. In the presence of GSH, Fe‐MOFs undergo a faster responsive degradation. As shown in Figure 5, Fe^3+^ can be reduced to Fe^2+^, which not only consumes GSH but also directly participates in the Fenton reaction, generating highly toxic hydroxyl radicals that exacerbate oxidative stress in the tumor microenvironment, damage cellular components such as lipid peroxidation, and thereby induce cell death, including ferroptosis.^[^ ^73^, ^74^ ^]^ Additionally, Fe^3+^ ions, which possess vacant orbitals that can facilitate the formation of coordination catalytic active centers, act as potent catalase catalysts. This property enables Fe^3+^ to efficiently convert intratumoral H_2_O_2_ into oxygen and water, alleviating tumor hypoxia and enhancing tumor responsiveness to oxygen‐dependent therapies such as photodynamic therapy (PDT) and sonodynamic therapy (SDT).^[^ ^75^, ^76^ ^]^

However, free Fe^3+^/Fe^2+^ carries the risk of being released from its delivery system and potentially harming normal tissues. As carriers for Fe ions, Fe‐MOFs ensure the ions remain within their framework, preventing dislodgement and reducing the potential for side effects, thereby enhancing the safety and efficacy of treatments. Moreover, the porous nature of Fe‐MOFs permits high loadings of bioactive agents, offering new hope for cancer treatment by facilitating synergistic effects between ferroptosis and other therapeutic strategies, including PDT, SDT, photothermal therapy (PTT), chemotherapy, and immunotherapy, and in vivo imaging.^[^ ^25^, ^75^, ^77^, ^78^, ^79^ ^]^

#### 2.2.4 Ferroptosis as an Antimicrobial Strategy

The dual role of Fe as both a nutrient and a potential toxin is pronounced in the microbial world. Although Fe is an essential micronutrient required for the growth of almost all bacteria, both its deficiency and excess can impede bacterial proliferation. Manipulation of Fe metabolism to induce bacterial death is a promising approach. Fe‐chelators function by binding free Fe ions and exhibit effective bactericidal activity against microbes such as *Staphylococcus aureus* (*S. aureus*) and *Candida albicans* (*C. albicans*) through the depletion of Fe ions.^[^ ^80^, ^81^, ^82^ ^]^ However, the utilization of Fe‐chelators may result in detrimental effects on host cells in circulation or within deep tissues. High concentrations of Fe ions can induce ferroptosis‐like cell death in most Gram‐negative bacteria such as *Escherichia coli* (*E. coli*) and *Pseudomonas aeruginosa* (*P. aeruginosa*), which contain GSH.^[^ ^83^, ^84^ ^]^ However, the mechanisms of iron homeostasis and oxidative stress responses in bacteria lacking GSH may differ from those in mammalian cells.^[^ ^85^ ^]^ Particularly, these bacteria might use alternative sulfur‐containing molecules or different redox strategies. *S. aureus* uses bacillithiol as an antioxidant molecule.^[^ ^86^, ^87^ ^]^ *Mycobacterium tuberculosis* (*M. tuberculosis*), a member of the *Mycobacteria* genus, employs mycothiol as its primary low‐molecular–weight thiol to maintain intracellular thiol‐disulfide homeostasis in the presence of oxidative stress.^[^ ^88^ ^]^ The diverse redox systems within bacteria contribute to their varying sensitivity to ROS‐related therapies such as chemodynamic therapy (CDT) and PDT. In particular, when multiple strategies are employed simultaneously, it is important to conduct mechanistic research focusing on varying redox to prevent the emergence of bacterial resistance. In the context of bacterial infections within organisms, macrophages can utilize the iron transport protein ferroportin system to transport Fe^2+^ into intracellular bacterial compartments. The accumulation of Fe^2+^ in these compartments can result in oxidative damage and ferroptosis in the engulfed bacteria.^[^ ^89^ ^]^ Given that the concentration of H_2_O_2_ within bacterial cells may be lower than in tumor cells, iron‐based nanomaterials that rely on the Fenton reaction to produce ROS may not generate sufficient ROS to be therapeutically effective against bacteria. Consequently, supplementary strategies are needed to regulate iron release and amplify ROS production. Inspired by the success of iron‐based antitumor therapies, an increasing number of synergistic strategies are being developed to achieve targeted and efficient antibacterial effects, while minimizing harm to the host's normal cells.^[^ ^90^ ^]^

#### 2.2.5 Matériaux de I'Institut Lavoisier Series Fe‐MOFs

**Figure 6:**
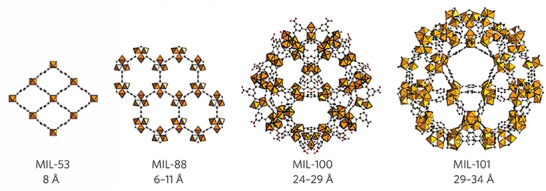
Representative MIL series Fe‐MOFs with pore size. Reproduced with permission.^[^ ^56^ ^]^ Copyright 2009, Nature.

Among the library of Fe‐MOFs, the Matériaux de I'Institut Lavoisier (MIL) series iron (III) carboxylate structures are the most promising ones for biomedical applications (**Figure** **6**), with some, such as MIL‐53(Fe),^[^ ^91^ ^]^ MIL‐88(Fe) series,^[^ ^92^ ^]^ MIL‐100(Fe),^[^ ^93^ ^]^ and MIL‐101(Fe),^[^ ^94^ ^]^ that have been successfully tested in vivo.^[^ ^95^, ^96^, ^97^, ^98^, ^99^ ^]^ In particular, the mesoporous iron trimesate MIL‐100(Fe) has attracted considerable research interest as a drug carrier, due to its substantial specific surface area (BET surface area around 2000 m^2^ g^−1^), large pores (with cages of 25 and 29 Å), and good stability within biological environments. The MIL‐88 series can be subclassified into MIL‐88A/B/C/D based on the types of dicarboxylic acids utilized in their synthesis.^[^ ^92^ ^]^ Both MIL‐53 and MIL‐88 exhibit a large structural flexibility, endowing the material with controllable pore sizes and internal cavity environments, potentially enhancing the entry and controlled release of guest molecules. Functionalization during synthesis with carboxylic acids bearing specific functional groups allows for the incorporation of these groups into the MOF structure, as seen with BDC‐NH_2_ (BDC: benzene‐1,4‐dicarboxylic acid) synthesized MIL‐88B(Fe)‐NH_2_ and MIL‐101(Fe)‐NH_2_, which have been utilized extensively in recent bio‐related researches.^[^ ^44^, ^96^, ^100^ ^]^ This modification can alter the surface chemistry, hydrophilic/hydrophobic balance, and size morphology of the MOFs. For instance, Mo et al. synthesized MIL‐101(Fe) nanoparticles using 1,4 BDC as a ligand, initially resulting in non‐uniform particle sizes larger than 1 µm, which is unsuitable for in vivo applications.^[^ ^44^ ^]^ By replacing 1,4 BDC with 2‐amino‐1,4‐BDC (BDC‐NH_2_) and optimizing experimental conditions such as temperature and reactants ratios, they obtained nanoparticles with a uniform size of ≈200 nm.^[^ ^44^ ^]^ And it can be suitable for post‐modification through amide coupling reactions. For instance, Song et al. utilized MIL‐88B(Fe)‐NH_2_ to immobilize glucose oxidase (GOx) via amide coupling.^[^ ^101^ ^]^ Furthermore, the robust coordination capacity of Fe with carboxylate ligands enables the doping of other metals into MIL series MOFs, resulting in the formation of mixed‐metal–based MOF composites. This approach leverages the actions of various metals, enhancing the potential for combined or synergistic efficacy.^[^ ^102^, ^103^, ^104^ ^]^ For instance, Li et al. developed a novel nanozyme‐based probe by incorporating Pd and Pt into MIL‐100(Fe), which was successfully applied for the detection of *S. aureus*.^[^ ^102^ ^]^ However, the incorporation of noble metals raises production costs, potentially hindering commercialization. In another study, Akbar et al. utilized mixed metals (Fe and Co) to synthesize MIL‐88B.^[^ ^104^ ^]^ The presence of mixed valence metal ions enhanced peroxidase‐like activity, enabling it to catalyze the decomposition of H_2_O_2_ to generate hydroxyl radicals for CDT. This approach significantly enhanced the anticancer activity against HepG2 cells. However, introducing multi‐metallic components necessitates carefully considering changes in the degradation rate of the composites and their potential toxicity to normal cells, ensuring they are effective and safe in biomedical applications.

## 3 Synthesis, Characterization, Surface Engineering, and Shaping of Fe‐Based MOFs

### 3.1 Synthesis Routes

#### 3.1.1 Solvothermal and Hydrothermal Routes

Solvo‐ or hydro‐thermal syntheses are typical methods for the fabrication of MOFs, yielding high‐quality single crystals and revealing novel MOF structures. These methods operate under self‐generated pressure, contingent on temperatures exceeding the solvent's boiling point. Such techniques capitalize on conditions conducive to dissolving ligands with low solubility and stabilizing metastable or kinetic phases over thermodynamic ones. The solvent repertoire for these processes is extensive, encompassing *N*,*N*‐diethylformamide, *N*,*N*‐dimethylacetamide (DMA), DMF, dimethyl sulfoxide, alcohols, or water.

The synthesis of MIL‐100(Fe) can be used as an illustrative example. In 2012, Seo et al. demonstrated the large‐scale synthesis of MIL‐100(Fe) without the use of HF.^[^ ^105^ ^]^ Later research has focused on the synthesis of MIL‐100(Fe) at room temperature (RT) through simple and scalable green methods without the use of organic solvents.^[^ ^106^, ^107^, ^108^, ^109^, ^110^ ^]^ However, the use of organic or inorganic bases as additives to facilitate the dissolution of reagents and accelerate the reaction is common. These additives, however, have the disadvantage of hindering the uniformity of nanoparticle dimensions and frequently increasing the average particle size. Subsequently, Panchal et al. developed a synthesis of MIL‐100(Fe) in water without the use of additives for solubilization, thereby paving the way for the green production of Fe‐MOFs.^[^ ^111^ ^]^ Similarly, MIL‐127(Fe), a microporous Fe tetracarboxylate MOF, can also be synthesized under green conditions, without toxic solvents.^[^ ^55^ ^]^ Currently, MIL‐53(Fe) synthesis still relies on the use of solvents such as DMF and MeOH, which are recognized for their efficacy in the process but raise toxicity concerns, although some of its functionalized versions can be produced in green solvents.^[^ ^47^, ^112^, ^113^ ^]^

Based on the principles of green chemistry, the production of Fe‐MOFs should discard as much as possible the use and generation of hazardous substances during the chemical process, thereby promoting environmentally friendly synthesis. For biomedical applications, the synthesis methods should avoid toxic solvents and/or auxiliary chemicals. For example, DMF and HF should be avoided. While these solvents or additives have proven highly effective in the crystallization of MOFs, their toxicity and subsequent disposal issues make them unsuitable for real applications. The associated environmental and economic costs further exacerbate their limitations. As a result, greener alternatives such as water or alcohols are increasingly being explored as viable solvent systems. By integrating these greener solvents into synthetic pathways, it becomes possible to maintain production efficiency while significantly reducing environmental impact, in line with the broader goals of sustainable industrial‐scale production of Fe‐MOFs.

#### 3.1.2 Microwave‐Assisted Route

Microwave heating, which uses electromagnetic waves to interact with polar molecules, provides a fast and energy‐efficient method for synthesizing Fe‐MOFs such as MIL‐100(Fe), MIL‐101(Fe), and MIL‐101(Fe)‐NH_2_.^[^ ^94^, ^114^ ^]^ This technique can significantly reduce reaction times from days to minutes and enable a control of the particle size and polydispersity. In addition, microwave‐assisted hydro/solvothermal synthesis is considered as one of the important strategies in the field of green chemistry due to its high energy efficiency and reduced solvent use. This technique not only shortens the reaction time, but also significantly fasten the solubilization of the reactants, thus minimizing the use of solvents and reduce environmental pollution. However, one of the drawbacks of the microwave‐assisted method is the presence of defects, and future studies should focus on optimizing the synthesis conditions to improve the purity and stability of the products.^[^ ^32^ ^]^

#### 3.1.3 Sonochemical Route

Ultrasound interacts with liquid solutions to generate small bubbles that act as hot spots, which subsequently grow and collapse, leading to localized increases in pressure and temperature promoting the crystallization of solids. This phenomenon enhances the effective collisions between particles, thereby facilitating the rapid and uniform formation of small‐sized crystals. For instance, the synthesis of MIL‐88A(Fe) via the sonochemical route requires only 10 min, while the solvothermal route typically takes ≈24 h.^[^ ^115^ ^]^ Moreover, the morphology, surface area, and porosity of MIL‐88A(Fe) are significantly influenced by the ultrasonic conditions and reaction time employed in the sonochemical reactor.^[^ ^115^ ^]^ This method is advantageous for producing monodispersed nanoparticles which is suitable for biomedical applications. However, due to the complexity of ultrasonic equipment and the often limited yields, scaling up this technique for industrial production remains challenging.^[^ ^116^ ^]^

#### 3.1.4 Mechanochemical Synthesis

Mechanochemical grinding represents a promising alternative to traditional solvent‐intensive methods. In this technique, solid reagents are typically ground under conditions either devoid of solvents or with minimal solvent use at room temperature. The array of devices utilized in mechanochemical synthesis is diverse, including ball mills, kitchen grinders, and twin‐screw extruders. For example, Samal et al. employed a kitchen grinder for the multi gram‐scale synthesis of MIL‐100(Fe) and investigated its pore‐size‐dependent adsorption properties.^[^ ^117^ ^]^

One of the significant advantages of (thermo)mechanochemical synthesis is its short reaction times and high conversion rates while avoiding the use of solvents, although it requires a very high yield to avoid the use of solvents as an extra‐step to wash and purify the MOF. This method leads usually to a reduction of the environmental impact associated with traditional solvent synthesis routes. This has led to a faster and simpler route to Fe‐MOFs production, which enhances the sustainability of this technique for large‐scale applications.^[^ ^118^ ^]^ However, this method suffers from several limitations: i) it cannot be applied to any MOF system and ii) it is difficult to control the particle size; this is mainly due to the limited understanding of the formation mechanisms. In addition, an excessive use of the mechanical energy can lead to the amorphization of MOFs.^[^ ^119^ ^]^ A more comprehensive and systematic understanding of the mechanochemical mechanisms is thus required to translate this technique into a well‐understood, quantitative field of chemistry.^[^ ^118^ ^]^

#### 3.1.5 Scale‐Up

Among many available synthesis routes, those conducted at low temperatures and atmospheric pressure are more suitable for addressing the dual challenges of economic and environmental sustainability. Also, it is important to consider the replacement of toxic solvents with greener alternatives in light of considerations of human health, environmental impact, operational feasibility, and stringent environmental regulations, as this has become a prerequisite for scaling up production. Furthermore, one shall rely on inexpensive, non‐toxic, and predominantly commercialized linkers from the early stages. Typical examples include terephthalic acid, trimesic acid, fumaric acid, and isophthalic acid, which are widely used in the synthesis of Fe‐MOFs. For instance, MIL‐100(Fe), synthesized from trimesic acid, exhibits promising performance across various applications, suggesting substantial commercial potential. Recently, some of us have achieved the production of MIL‐100(Fe) at kg scale, paving the way for its larger scale production at industrial scale, aiming at enhancing efficiency and reducing costs.^[^ ^42^ ^]^ This study demonstrates the feasibility of scaling up MIL‐100(Fe) synthesis using both nitrate and sulfate‐based routes under ambient pressure conditions. The nitrate route achieves a high specific throughput yield (STY) of 10 kg m^−3^ per day, with a production cost estimated at $58 per kg. The sulfate route shows even higher STY, nearing 120 kg m^−3^ per day, potentially lowering the production cost to below $30 per kg. Techno‐economic analyses indicate that the choice of synthesis route has a significant impact on the capital investment required. Additionally, bio‐sourced or biomass‐derived linkers, due to their minimal environmental impact and sustainability, are garnering attention. Fumaric acid, an endogenous ligand produced biologically, is utilized in synthesizing the microporous, flexible iron MOF, MIL‐88A(Fe). With fumaric acid‐derived drugs already approved by the Food and Drug Administration (FDA), MIL‐88A(Fe) shows significant potential for biomedical applications. This MOF can easily be scaled‐up at different particle sizes in water under atmospheric pressure.^[^ ^50^ ^]^

Spray‐drying is a promising continuous‐flow synthesis process with a high STY and solvent recovery capability, eliminating the need for further shaping steps.^[^ ^116^ ^]^ The scale‐up of spray‐drying methods has already been demonstrated for the industrial production of HKUST‐1 and ZIF‐8.^[^ ^120^ ^]^ In the case of Fe‐MOFs, the spray‐drying synthesis route has been reported to prepare MIL‐88A(Fe), MIL‐88B(Fe), and MIL‐100(Fe).^[^ ^121^, ^122^ ^]^ However, the resulting MOFs may exhibit lower crystallinity or porosity, likely due to the accelerated crystal growth in the spray‐drying process. This can be attributed to kinetically driven crystal growth, which contrasts with the slower, thermodynamically controlled growth typically observed in solvothermal synthesis. This is also not straightforward to control the particle size. Thus, further research is needed not only to better understand the formation mechanisms but also to extend this method to MOFs with slower crystallization kinetics. Alternatively, one can use spray‐drying for the preparation of micron‐size agglomerates of Fe‐MOF nanoparticles in a view of biomedical applications.^[^ ^123^ ^]^

Startups and industries focusing on the large‐scale production of Fe‐MOFs are beginning to commercialize their products. For example, Framergy produces AYRSORB F250 (based on MIL‐127(Fe)) and AYRSORB F100 (MIL‐100(Fe)) under atmospheric pressure, while Promethean Particles manufactures Fe‐BTC (BTC: benzene‐1,3,5‐tricarboxylic acid) and MIL‐100(Fe) via hydrothermal routes.^[^ ^116^ ^]^ Although these commercial products are primarily aimed at applications in water capture, catalysis, or gas purification, they also hold promise for the bulk production of Fe‐MOFs–based biomedical products.

#### 3.1.6 Washing and Activation

The washing and activation processes of MOFs utilized in DDS are as crucial as their synthesis protocols. Unreacted linkers or high‐boiling‐point solvents, are often retained within the pores or on the external surfaces of the particles, which decreases the drug‐loading capacity of MOFs. To mitigate this issue, washing or purification procedures are employed. The most straightforward approach is to wash with high‐solubility solvents; however, the majority of these, such as DMF and DMA, are toxic and unsuitable for biomedical use. Consequently, safer alternatives, such as water or ethanol, are preferred, despite their limited solvating power necessitating multiple washing cycles. Residual linker or solvent removal is typically tested using Fourier transform infrared spectroscopy (FT‐IR), thermal gravimetric analysis, and nitrogen sorption measurements. An alternative method for solvent removal is lyophilization, a low‐temperature process involving freezing the material, and reducing pressure to sublimate the solvent, thereby achieving high porosity.^[^ ^124^ ^]^ However, due to its high energy demands and lengthy operational periods, lyophilization is not ideal for the economic industrial‐scale production of MOFs.^[^ ^116^ ^]^

### 3.2 Characterization Techniques

Powder X‐ray Diffraction (PXRD) is one of the most employed techniques for structural characterization of MOFs. By analyzing diffraction patterns, the crystalline structure of Fe‐MOFs can be identified, and the phase purity of samples verified. This is crucial for ensuring the reproducibility and stability of Fe‐MOF synthesis. Notably, for flexible Fe‐MOFs, their diffraction patterns may change due to variations in pore size driven by activation temperatures, encapsulation of guest molecules, or solvent interactions, which makes the structural analysis more complex.

Morphological analysis, such as scanning electron microscopy and transmission electron microscopy, is pivotal in revealing the morphology and particle size of Fe‐MOFs.

Dynamic light scattering is extensively used to assess the hydrodynamic diameter and particle size distribution of Fe‐MOF nanoparticles in solution, predicting their potential for aggregation in target solutions. Zeta potential measurements provide quantitative insights about the surface charge of Fe‐MOFs, which directly influences their colloidal stability. A higher magnitude of zeta potential, whether positive or negative, typically indicates good colloidal stability due to electrostatic repulsion between particles, reducing the likelihood of aggregation. The surface charge also impacts the interaction of Fe‐MOFs with cell membranes, influencing cellular uptake.

Gas sorption measurements, particularly nitrogen sorption combined with the BET method and Barrett–Joyner–Halenda analysis, are employed to evaluate the surface area, pore size distribution, and pore volume of Fe‐MOFs before and after loading guest molecules. However, structural transitions in flexible Fe‐MOFs from a narrow‐pore to a closed‐pore configuration can restrict access to internal surface areas, thereby affecting the outcomes of BET analysis, requiring additional analysis such as CO_2_ adsorption isotherms.^[^ ^91^ ^]^

Surface and chemical characterization techniques, including FT‐IR and X‐ray photoelectron spectroscopy, provide insights into the surface functional groups and chemical composition of Fe‐MOFs. These techniques are vital for tailoring Fe‐MOFs for targeted drug delivery systems by ensuring the presence of functional groups conducive to bioconjugation.

The biocompatibility of Fe‐MOFs can be preliminarily assessed through cytotoxicity assays, such as MTT, CCK‐8, and live/dead assays. These studies are imperative to ensure that Fe‐MOFs can be safely utilized in biomedical applications without eliciting adverse biological responses, even if this does not guarantee the in vivo toxicity.

### 3.3 Surface Engineering

Surface engineering of nanomaterials can enhance the stability, biocompatibility, and targeting capabilities of materials. Technological advancement in this field is crucial for the development of biomedical applications such as biosensing, anticancer therapy, and antimicrobial therapy. By modifying the surface of Fe‐MOFs, researchers can tailor their interactions with biological environments and also add extra biological functionalities to them thereby achieving increased efficacy and safety in their intended applications. **Table** **3** presents a summary of recent advances in surface engineering of Fe‐MOFs.

**Table 3:** Recent advances in surface engineering of Fe‐MOFs.

Fe‐MOFs	Functional molecules	Application	In vitro/In vivo model	Ref.
MIL‐100(Fe)	chitosan	Anticancer	breast cancer cells, including SKBR3, MCF‐7, MDA‐MB‐231, and BT549	[125]
MIL‐100(Fe)	chitosan	DDS	Caco‐2	[126]
MIL‐101(Fe)‐NH_2_	chitosan	Anticancer	HepG2 cells	[127]
MIL‐88B(Fe)‐NH_2_	thiamine pyrophosphate (TPP), chitosan oligosaccharides (COS)	Anticancer	CT26 tumor‐bearing mice	[96]
FcMOF	lactose derivative	Anticancer, Bioimaging	mice by injection of H22 cancer cells into the hind leg	[128]
Fe‐Tetrakis (4‐carboxyphenyl) porphyrin (TCPP)	lactobionic acid	Anticancer, Bioimaging	C5WN1 tumor‐bearing mice	[129]
Fe‐BDC‐NH_2_	pectin	Antimicrobial	*R. solani*	[130]
MIL‐100(Fe)	β‐cyclodextrin(CD), graphene oxide (GO)	Anticancer	A549 cells	[131]
MIL‐100(Fe)	malate‐crosslinked β‐CD (CD‐MO)	DDS		[132]
SMnxFeyH MOFs	hyaluronic acid (HA)	Anticancer	B16F10 tumor‐bearing mice	[133]
Fe‐TK MOF	HA	Anticancer	mice by subcutaneous injection of 4T1 cells	[134]
Fe‐BDC‐NH_2_	HA	Anticancer, Bioimaging	4T1 cell tumor‐bearing mice	[135]
Hollow Fe‐MOF	HA	Anticancer	H22 tumor‐bearing mice	[136]
FeTBP	HA	Anticancer	MB49 tumor‐bearing mice	[137]
MIL‐101(Fe)	polydopamine (PDA)	Anticancer	mice by axillary injection of H22 cancer cells	[138]
MIL‐53(Fe)	polyvinylpyrrolidone (PVP)	Antimicrobial	*S. aureus*‐infected mice	[139]
MIL‐53(Fe)	poly (2‐methacryloyloxyethyl phosphorylcholine) (PMPC)	Anticancer	mice by axillary injection of CT‐26 cancer cells	[140]
MIL‐101(Fe)‐NH_2_	poly(acrylic acid) (PAA)	Biosensing		[141]
MIL‐100(Fe)	PAA	Bone repairing	MG‐63 cells	[142]
MIL‐101(Fe)‐NH_2_	Pluronic F‐127	Anticancer	4T1 tumor‐bearing mice	[44]
TA‐Fe ^3+^ MOF	bovine serum albumin (BSA)‐folic acid(FA)	Anticancer	mice by injection of B16F10 cells into the right axilla	[143]
Fe/ZIF‐8	FA	Anticancer, Bioimaging	4T1 tumor‐bearing mice	[144]
MIL‐100(Fe)	PEG		murine macrophage cell line	[145]
MIL‐100(Fe)	PEG5000, Stp10‐C	Bioimaging	N2A cells	[146]
MIL‐101(Fe)	PEG‐FA	Anticancer, Bioimaging	U87MG tumor‐bearing mice	[147]
MUV‐2	8‐Br octanoic acid and FA	Anticancer	HeLa cells	[148]
Fe‐BDC‐NH_2_	phenylboronic acid	Biosensing		[149]
MIL‐101(Fe)	Cu‐tannic acid(TA)	Anticancer	HepG2 cells	[150]
Fe‐BDC‐NH_2_	lipid bilayer coating, a tumor‐homing peptide (RGD)	Anticancer, Bioimaging	4T1 cell tumor‐bearing mice	[151]
Fe‐porphyrin MOF	neutrophil membrane (NM)	Anticancer, Bioimaging	4T1 cell tumor‐bearing mice	[152]
MIL‐100(Fe)	human cell membranes	Anticancer	MDA‐MB‐468 cells	[153]
MIL‐88B(Fe)‐NH_2_	macrophage membrane	Anticancer, Bioimaging	CT‐26 tumor‐bearing mice	[154]
Fe‐2‐MIM	A549 cell membrane	Anticancer, Bioimaging	A549 tumor‐bearing mice	[155]
MIL‐53(Fe)	red blood cell (RBC) membrane	Anticancer	mice by injection of MCF‐7/ADR cells	[95]
Co‐Fc MOF	oral cancer cell membranes	Anticancer, Bioimaging	mice by subcutaneous injection of CAL‐27 cells	[156]
Fe‐TCPP	homologous cancer cell membrane (CCM)	Anticancer, Bioimaging	4T1 cell tumor‐bearing mice	[157]
MIL‐53(Fe)	CuO, ZnO	Antimicrobial	*E. coli* and *S. aureus*	[158]

#### 3.3.1 Surface Engineering for Enhanced Stability and Efficacy

MOFs exhibit remarkable physical and chemical properties for biomedical applications, but they often encounter stability issues, poor dispersion, and problematic systemic distribution in the body.^[^ ^159^ ^]^ Particularly, MOFs tend to aggregate rapidly in biological media, forming clusters that can easily induce vascular occlusion. As previously discussed, most Fe‐MOFs are unstable in PBS due to the phosphate groups acting as Lewis bases, competitively binding with the central iron ions. To enhance stability, the frameworks can be encapsulated with biocompatible materials including carbohydrates,^[^ ^127^, ^129^, ^133^ ^]^ biocompatible polymers,^[^ ^139^, ^140^, ^142^ ^]^ organic acids,^[^ ^150^ ^]^ or lipid shells.^[^ ^151^ ^]^

**Figure 7:**
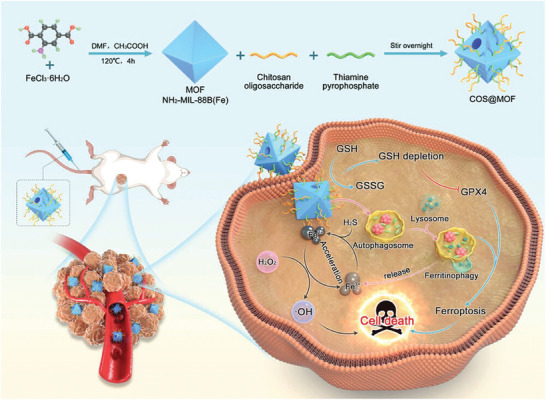
Schematic diagram of surface‐engineered COS@MOF with amplifying autophagy to enhance ferroptosis for nanocatalytic tumor therapy. Reproduced with permission.^[^ ^96^ ^]^ Copyright 2023, Wiley.

In 2015, Gref et al. developed nanoparticles of MIL‐100(Fe) coated with cyclodextrin phosphate (CD‐P).^[^ ^160^ ^]^ CDs are water‐soluble cyclic oligosaccharides, classified as α‐, β‐, and γ‐CD based on the number of glucose units (6, 7, or 8, respectively).^[^ ^161^ ^]^ The CD‐based polymer coatings enhance the stability of nanoMOFs during storage and facilitate the encapsulation of the therapeutic agents into the CD coating. The bulky CD‐P molecules are too large to penetrate the hexagonal windows of the MOFs, but they can anchor securely to the available iron sites on the exterior surfaces through synergistic coordination. The presence of the coating does not compromise the drug‐loading capacity of the nanoMOFs. Recently, Gref et al. have further functionalized MIL‐100(Fe) with oligomers made from CD‐MO to encapsulate adenosine monophosphate (AMP).^[^ ^132^ ^]^ This surface engineering strategy slows down the degradation of the bare MIL‐100(Fe) nanoMOFs but promote the release of AMP, as evidenced by testing of the concentration of free organic linker and AMP during degradation using HPLC. Typically, when the surface engineering strategy slows down the degradation of the bare MOF, it is expected also to reduce the release rate of the encapsulated drug. However, in this example, surface modification materials (CD‐MO) accelerated drug release possibly by dissolving, swelling, or increasing the permeability to water molecules. Moreover, as demonstrated in this example, the surface engineering strategy might not systematically succeed in avoiding the burst release profile of the drug. Sontakke et al.^[^ ^131^ ^]^ functionalized MIL‐100(Fe) with β‐CD and GO to enhance the drug delivery capabilities of the pristine MIL‐100(Fe). The resulting β‐CD–functionalized MIL‐100(Fe) nanocomposites exhibit enhanced solubility and biocompatibility and hold promise for the development of stimuli‐responsive drug delivery systems. Moreover, the encapsulation of GO, which is known for its extensive surface area and functional groups, substantially increases the drug‐loading capacity of the nanocarriers. This multifaceted functionalization strategy significantly improves the efficiency and functionality of MIL‐100(Fe) as a drug delivery platform, offering new avenues for targeted and controlled release applications; however, one could wonder about the fate of GO once inside the body as GO is not degradable and is too large to be excreted by urines, raising potential long term toxicity issues. In 2017, Horcajada et al. proposed the biofriendly preparation of a novel oral nanocarrier, utilizing MIL‐100(Fe) coated with the bioadhesive polysaccharide chitosan (CS).^[^ ^126^ ^]^ This coating preserves the high porosity and crystalline structure of the nanoparticles, without inducing toxicity issues. Furthermore, the CS coating endows the nanoparticles with significant advantages, including: i) enhanced chemical stability in simulated oral fluids; ii) additional biological functionality; iii) superior ability to bypass intestinal barriers; and iv) reduced recognition by the immune system.^[^ ^126^ ^]^ These features make these CS‐coated nanoparticles highly promising for the oral delivery of active pharmaceutical ingredients. In a recent study, Quijia et al. demonstrated that coating MIL‐100(Fe) nanoparticles with chitosan not only enhances their stability but also facilitates cellular uptake and enables the sustained release of encapsulated piperine.^[^ ^125^ ^]^ This release exhibited pH‐dependent character, with faster release at lower pH values, as solubility of chitosan is higher at a lower pH due to the ionization of d‐glucosamine residues, resulting in increased polymer swelling and faster drug release. The chitosan surface modification enhanced the nanoparticles' interactions with cellular environments and extended the release duration of the piperine, resulting in only 17% release over 7 days at pH 5. While this sustained release is beneficial for prolonged therapeutic effects, it is also important to consider the risk of an incomplete drug release. Based on a derivative of CS, Du et al. developed an innovative therapeutic strategy by utilizing an etched and surface‐modified Fe‐based MOF, named COS@MOF.^[^ ^96^ ^]^ In **Figure** **7**, MIL‐88B(Fe)‐NH_2_ was specifically modified with COS, which act as autophagy agonists and are etched using TPP. This etching process creates an open cavity structure that not only improves the adsorption of reactive molecules but also increases the Fe(II)/Fe(III) ratio, enhancing the MOF's catalytic activity and ROS generation. Both in vitro and in vivo studies confirm that COS@MOF disrupts iron homeostasis and amplifies autophagy, leading to increased ROS production and induced ferroptosis. To develop biosensing platforms with more precise and reliable detection capabilities, Supianto et al. conducted a surface modification of MIL‐101(Fe) with PAA, which enhanced the stability of the colloids and provided protection against interference from proteins and small molecules.^[^ ^141^ ^]^ This modification significantly improved the sensitivity of the assay compared to traditional gold nanoparticle(AuNP)‐based lateral flow assays (LFAs). As a result, they achieved a remarkable limit of detection (LOD) of 0.012 nm in Tris‐HCl buffer and 0.009 nm in desalted urine, while the LOD of the traditional AuNP‐based LFA is 0.598 nm in desalted urine.

In the field of cancer therapy, Zhang et al. developed a novel strategy that employs a biodegradable zwitterionic polymer, PMPC, to coat defective MIL‐53(Fe), which was loaded with the ferroptosis inducer atorvastatin (ATV).^[^ ^140^ ^]^ This polymer‐based surface modification is designed to enhance tumor accumulation and extend circulation time by delivering Fe ions and ATV. Upon internalization by cancer cells, the nanoplatform underwent degradation in response to the GSH present at the tumor site. This resulted in the depletion of GSH and the concurrent release of ATV and Fe^2+^, disrupted mevalonate (MVA) biosynthesis and further reduces GSH levels, which subsequently suppressed GPX4 expression. Concurrently, the released Fe^2+^initiated the Fenton reaction, resulting in the generation of high levels of ROS. These interrelated processes culminated in lethal lipid peroxidation (LPO), effectively inducing ferroptosis. Through in vivo tests, the nanoplatform demonstrated significant tumor growth inhibition, with an 86% tumor‐inhibitory ratio, and exhibited good biocompatibility. To develop antimicrobial systems, Edlo et al. demonstrated that functionalizing MIL‐53(Fe) with metal oxides (CuO and ZnO) significantly enhanced its efficacy in combating microbial resistance.^[^ ^158^ ^]^ In their study, CuO@MIL‐53(Fe) and ZnO@MIL‐53(Fe) nanocomposites were synthesized through the partial thermal decomposition of their respective metal acetates within a MIL‐53(Fe) matrix. The surface functionalization and integration of these metal oxides not only preserved the structural stability of MIL‐53(Fe) but also achieved the release of multiple metal ions. This release acted synergistically to increase the antibacterial activity against both Gram‐positive and Gram‐negative bacteria, underscoring their potential as effective antimicrobial agents.

#### 3.3.2 Surface Engineering for Improved Targeting Ability

While MOFs can encapsulate large quantities of therapeutic agents, they frequently fail to deliver these drugs in a targeted manner, resulting in the undesirable accumulation of the agents in non‐target tissues. To overcome this limitation, researchers have explored strategies to enhance the targeting capabilities of MOFs by functionalizing their surfaces with specific targeting ligands, including FA,^[^ ^143^, ^144^, ^147^ ^]^ HA,^[^ ^134^, ^135^, ^136^, ^137^ ^]^ and lipid or cell membrane coatings.^[^ ^152^, ^153^, ^155^ ^]^ FA has been a pioneering targeting agent utilized in MOFs. The folate receptor (FR) α, a glycosylphosphatidylinositol‐anchored protein located on the cell surface, is frequently overexpressed in various types of cancer cells.^[^ ^162^ ^]^ This facilitates the selective delivery of folate‐conjugated MOFs to FR‐positive cancer cells via receptor‐mediated endocytosis, enhancing the efficacy and selectivity of the therapy and reducing toxicity to healthy tissues. Lázaro et al. developed a strategic approach to the surface modification of the Fe‐TTFTB MOF, termed NanoMUV‐2, utilizing 8‐bromooctanoic acid to impart hydrophobic characteristics and folic acid to confer hydrophilic and cancer‐targeting properties.^[^ ^148^ ^]^ These modifications effectively tailored the drug release kinetics and modulate cellular uptake routes. The incorporation of a folic acid coating significantly enhanced the delivery of the drug to HeLa cells, thereby optimizing the internalization of the drug through FR‐mediated pathways. Wang et al. employed a lactose derivative (Lac‐NH_2_), which they modified onto the surface of a ferrocene‐based MOF (FcMOF), denoted as Lac‐FcMOF, to enhance its dispersibility in water and endow it with targeted capabilities toward HepG2 cells.^[^ ^128^ ^]^ This modification not only enhanced the hydrophilicity of the MOF but also facilitated targeted delivery to liver cancer cells, exploiting the specific affinity of lactose for the asialoglycoprotein receptor expressed on HepG2 cells. Yang et al. employed pectin‐coated Fe‐BDC‐NH_2_ nanoparticles to encapsulate the pesticide thifluzamide (TF), to enhance the antimicrobial efficacy of TF in rice plants.^[^ ^130^ ^]^ The surface modification with pectin rendered the nanoparticles responsive to pectinase, an enzyme produced during the invasion by the pathogenic fungus *Rhizoctonia solani*. This modification also improved the wetting and adhesion properties of the nanoparticles on rice leaf surfaces, significantly enhancing the foliar retention of the pesticide. Moreover, the nanoparticles demonstrated remarkable bidirectional transport within the rice plants, enabling targeted delivery of the pesticide molecules. The TF@Fe‐MOF‐PT nanoparticles outperformed the traditional TF suspension in both in vitro bactericidal activity and greenhouse control efficacy. They exhibited the ability to disrupt mycelial mitochondria and reduce pathogenicity, while also promoting seedling growth by mitigating the phytotoxicity often associated with conventional pesticide formulations. This suggests a more efficient and sustainable method for pest and disease control in agriculture, highlighting its potential to improve crop protection strategies. Tang et al. modified the surface of a sensor with phenylboric acid (PBA) on the Fe‐BDC‐NH_2_, which played a crucial role in the enhanced detection of HepG2 cells.^[^ ^149^ ^]^ The PBA modification exhibited a specific affinity for sialic acid residues on the cell membrane, thereby facilitating targeted and efficient cell labelling. This PBA‐based surface engineering significantly enhanced the sensor's specificity and selectivity, enabling precise targeting and sensitive detection of cancer cells.

Traditional cell therapies, such as those utilizing stem cells or immune cells, exhibit challenges including potential immune rejection, ethical issues, and manufacturing complexities.^[^ ^152^ ^]^ The strategy of engineering nanomaterial surfaces with cell membranes emerges as a promising cell‐free biomimetic approach that offers hope in circumventing these limitations. In 2016, Cheng et al. pioneered the development of MOFs@cell membrane‐based biomimetic nanoplatforms.^[^ ^163^ ^]^ This cell membrane surface engineering strategy presents several advantages in developing Fe‐MOF based nanoplatform: i) Targeting: leveraging natural targeting and recognition abilities provided by cell membrane components, Fe‐MOFs coated with cell membranes can more effectively target specific tissues or tumors. This mimics the natural homing behaviors of cells to specific body sites. ii) Biocompatibility: This modification enhances the compatibility of Fe‐MOFs with biological environments, reducing immune responses, lowering potential toxicity, and increasing their effectiveness as carriers for drugs or imaging agents. iii) Protection: The cell membrane shields the active pharmaceutical ingredients within the Fe‐MOFs from burst degradation or clearance from the body, thus enabling more controlled and sustained release. Although the initial results are promising, the field remains in its infancy, primarily focusing on the treatment of breast cancer,^[^ ^95^, ^152^, ^153^, ^157^ ^]^ lung cancer,^[^ ^155^ ^]^ colorectal cancer,^[^ ^154^ ^]^ and oral cancer.^[^ ^156^ ^]^

**Figure 8:**
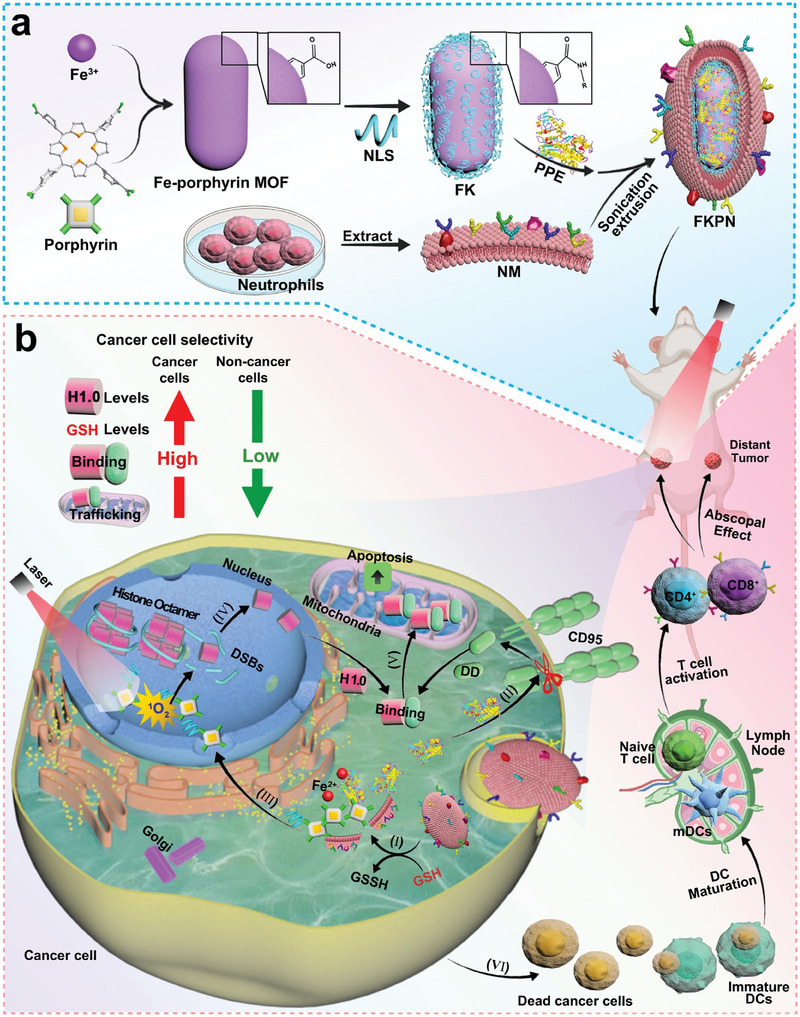
a) Schematic illustration of preparation of biomimetic nanodevice FKPN. b) Biological mechanism of FKPN for selective cancer cell killing and immune system activation through PPE and tumor‐specific nuclear ^1^O_2_ generator. NLS: nuclear localization signal; FK: Fe‐porphyrin MOF‐NLS; PPE: porcine pancreatic elastase; NM: neutrophil membrane; H1.0: histone H1 isoforms; DD: death domain; GSH: glutathione; GSSH: oxidized glutathione; DSBs: DNA double‐strand breaks; DCs: dendritic cells; mDCs: mature dendritic cells. Reproduced with permission.^[^ ^152^ ^]^ Copyright 2023, Nature.

In the field of breast cancer treatment, Rao et al. introduced a lipid bilayer coating to functionalize Fe‐BDC‐NH_2_, enhancing its stability and circulation time in the tumor environment.^[^ ^151^ ^]^ Additionally, the targeting ability toward breast cancer cells was optimized by incorporating a tumor‐homing peptide (RGD). While the modification steps for lipid bilayer and RGD are straightforward, the process of surface engineering introduces toxic reagents such as chloroform, potentially leading to cytotoxicity. Additionally, prolonged sonication is required, which may result in premature drug release. In another study, Cui et al. prepared FKPN, a biomimetic nanodevice employing a NM coated Fe‐porphyrin MOF for targeted tumor therapy (**Figure** **8**).^[^ ^152^ ^]^ This NM coating improved the tumor‐targeting capabilities of the nanodevice, which released its active components in response to elevated glutathione levels within cancer cells. Under laser irradiation, the released nuclear localization signal (NLS) peptide‐tagged porphyrin generated ROS, thereby enhancing the apoptosis mechanism with minimal host toxicity. In vivo studies showed that FKPN not only reduced primary tumors effectively but also stimulated systemic antitumor immunity, highlighting its potential as a novel, precision‐targeted, and immunologically active therapeutic agent for solid tumors. Peng et al. presented an innovative approach to camouflage MIL‐53(Fe) with RBCmembranes, creating a multi‐drug delivery platform targeting multidrug‐resistant cancer through combined ferroptosis–apoptosis mechanisms.^[^ ^95^ ^]^ This use of RBC membranes extended the circulation time of the nanoparticles, facilitating targeted delivery of ferroptosis inducers and chemotherapeutic drugs. The platform effectively depleted GSH and promoted ROS generation, leading to significant anticancer effects by promoting ferroptosis and disrupting cell membrane fluidity and permeability. Recently, Horcajada et al. coated MIL‐100(Fe) nanoparticles with cell membranes from human triple‐negative breast cancer (TNBC) cells to create a targeted drug delivery system.^[^ ^153^ ^]^ These cell membrane‐coated MIL‐100(Fe) nanoparticles exhibited improved colloidal stability, increased cellular uptake, and enhanced cytotoxicity against TNBC cells, demonstrating potential as an effective targeted antitumor therapy.

**Figure 9:**
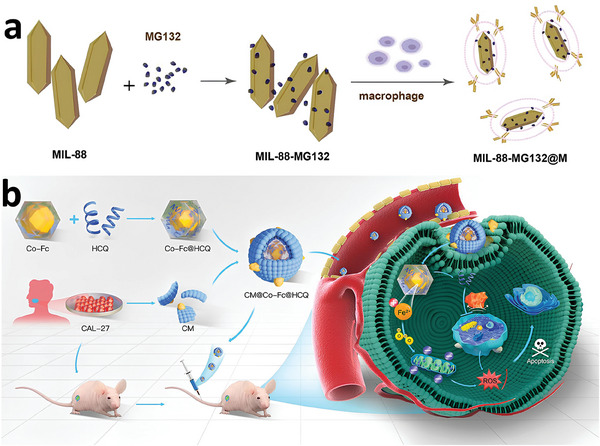
a) The surface engineering routine of MIL‐88‐MG132@M nanoparticles. Reproduced with permission.^[^ ^154^ ^]^ Copyright 2023, Wiley. b) Conceptual schematic diagram of the preparation process and intracellular mechanism of Co‐Fc nanoparticles. HCQ: hydroxychloroquine; CM: oral cancer cell membranes. Reproduced with permission.^[^ ^156^ ^]^ Copyright 2023, Wiley.

For colorectal cancer therapy, Bu et al. coated MIL‐88B(Fe)‐NH_2_ with macrophage membranes, the procedure is shown in **Figure** **9**a.^[^ ^154^ ^]^ The addition of a macrophage membrane coating not only targeted inflammatory tumor microenvironments, enhancing drug delivery and retention but also potentiated the immune response, thereby magnifying the therapeutic effects of the epigenetics‐based chemotherapy (Figure 9a). For oral cancer therapy, Chen et al. introduced a novel therapeutic strategy for oral squamous cell carcinoma (OSCC) using cell membrane@Co‐Fc@HCQ nanoparticles.^[^ ^156^ ^]^ In their study (Figure 9b), a cobalt‐ferrocene MOF (Co‐Fc) was loaded with hydroxychloroquine (HCQ) and coated with oral cancer cell membrane. The cell membrane coating enhanced homologous targeting and immune evasion, allowing for effective delivery of Co‐Fc, which generates ROS through the Fenton reaction to induce tumor cell death. Furthermore, the inclusion of HCQ inhibits autophagy in cancer cells, preventing the clearance of ROS and intensifying the cytotoxic effects, thus significantly improving the efficacy and biosafety of OSCC treatment through targeted, localized chemodynamic action.

### 3.4 Shaping

The direct application of MOFs, whether in powder form or in solution, presents significant challenges in biological applications due to issues such as aggregation, difficulty in recovery, and insufficient biocompatibility. The shaping process of MOFs enables their adaptation to a broader range of application scenarios and various administration methods. The fabrication of Fe‐MOFs into films has expanded their utility in wastewater treatment,^[^ ^164^, ^165^ ^]^ biosensing,^[^ ^166^ ^]^ and wound dressings.^[^ ^167^ ^]^ **Table** **4** presents a summary of recent advances in the shaping strategy of Fe‐MOFs targeting diverse applications.

**Table 4:** Recent advances in shaping of Fe‐MOFs.

Fe‐MOFs	Shaping substrates	Shaping objects	Loading capacity [wt%]	Application	Ref.
Fe‐BDC‐NH_2_	polyvinylidene fluoride (PVDF) powder, PVP	Mixed matrix membrane	7	Wastewater treatment	[165]
Lactose‐1‐aminonaphthalene‐Fe MOF	polycaprolactone (PCL), poly(amic acid)	Electrospun nanofibers		Immunosensor	[166]
Fe‐BDC	polyacrylonitrile (PAN)	Electrospun nanofibers	20	Tissue engineering	[167]
MIL‐101(Fe)	PAN@carbon	Electrospun nanofibers	40	Wastewater treatment	[164]
Fe‐BDC‐NH_2_	polycaprolactone (PCL), gelatin, glucose	Electrospun nanofibers		Wound dressing	[168]
MIL‐101(Fe)	carboxymethylated filter paper, cotton, and gauze	Flexible fiber composite materials	9	Water disinfection	[169]
MIL‐100(Fe)	cellulose	Paper	>70	Capture of polar volatile organic compounds	[170]
MIL‐101(Fe)‐NH_2_	polyvinyl alcohol	Paper chip		Detection of tetracycline (TC) pollution	[171]
MIL‐88B(Fe)‐NH_2_	PCL	Melt‐electrowritten scaffolds	20	Tissue engineering	[172]
Fe/Cu MOF	polyacrylamide (PAM)	Hydrogel		Wound dressing	[173]
Cu‐TCPP(Fe)	PVP	Microneedles (MN)	25	Transdermal delivery antitumor system	[174]

#### 3.4.1 Fibrous Membranes

Electrospinning is a straightforward and effective method for producing continuous fibrous membranes. Hundreds of polymers have been successfully electrospun into fibrous films that are characterized by simplicity of fabrication, high porosity, adjustability, and ease of recovery.^[^ ^175^ ^]^ These features render electrospun membranes ideal for industrial‐scale MOF loading. In the field of biomedicine, the high porosity of electrospun fiber networks facilitates material and gas exchange requirements during the wound healing process.^[^ ^168^ ^]^ Moreover, fibrous networks can mimic the structure of the extracellular matrix (ECM). This structural similarity to the ECM not only supports critical biological functions such as cellular attachment and proliferation but also optimizes the fibrous network's effectiveness in facilitating rapid blood clotting and wound healing.^[^ ^176^ ^]^ These benefits significantly enhance the functionality of MOF‐based materials in the wound care field. This strategic transformation of MOFs into functional films through electrospinning offers a promising avenue for enhancing their practical application in both environmental and biomedical fields, thus overcoming the inherent limitations of their conventional forms. Despite its advantages, electrospinning faces challenges in achieving high MOF loading. MOF particles becoming completely embedded within the polymer matrix is a potential risk, as it may hinder effective drug loading and release. Additionally, high‐loading liquid concentrations or poor dispersion can lead to aggregation of MOF nanoparticles. Moreover, reducing the use of toxic solvents is important.

**Figure 10:**
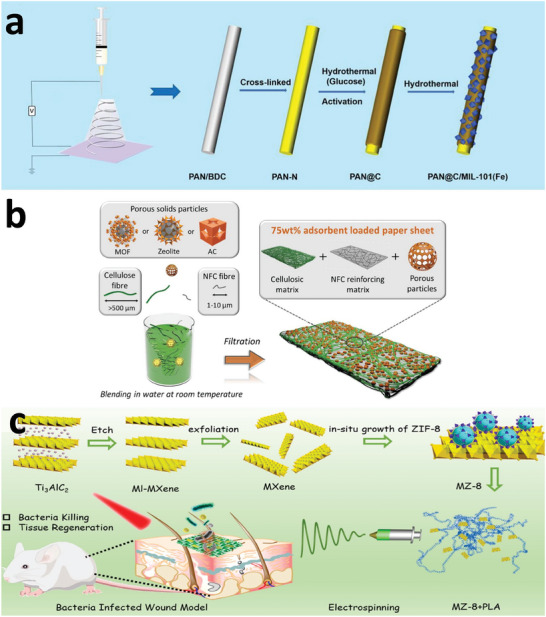
a) Schematic diagram of the preparation process of PAN@carbon/MIL‐101(Fe). Reproduced with permission.^[^ ^164^ ^]^ Copyright 2023, ScienceDirect. b) Schematic diagram of the green preparation process of the sheet containing different porous solids (Activated Carbon (AC), MOF, Zeolite) combined with an NFC‐reinforced cellulosic matrix. Reproduced with permission.^[^ ^170^ ^]^ Copyright 2023, Wiley. c) Schematic diagram of the preparation of the MZ‐8/PLA electrospun membrane. Reproduced with permission.^[^ ^177^ ^]^ Copyright 2021, ScienceDirect.

The fabrication of MOF‐based fibrous membranes can be achieved through three primary methods. The first method involves the in situ growth of MOFs on fibers that are synthetically produced via electrospinning or derived from natural sources. Teng et al. fabricated PAN@carbon@MIL‐101(Fe) composite nanofibers through electrospinning.^[^ ^164^ ^]^ The process is shown in **Figure** **10**a. The carbon‐coated PAN electrospun nanofibers were immersed in a DMF solution containing BDC and FeCl_3_, undergoing a solvothermal treatment. This process resulted in the uniform growth of MIL‐101(Fe) nanocrystals on the surfaces of the PAN@carbon nanofiber membranes. Notably, the content of MIL‐101(Fe) on the PAN@carbon/MIL‐101(Fe) reached up to 39.6 wt%. The described method offers significant advantages by enabling the uniform growth of MOFs on fibrous substrates with a high loading capacity. However, the process of synthesizing and activating the MOFs may compromise the structural integrity of the polymer membranes. This could render the technique unsuitable for certain polymers with poor chemical stability, such as gelatin. Furthermore, the residue of DMF is not easily removable, which may have potential toxicity and risk of blocking the pores of the MOFs, which could impede their functional performance. In another study, Tignol et al. reported a room‐temperature, water‐based, and straightforward process for fabricating high‐loaded (>70 wt%) porous material fibers (Figure 10b).^[^ ^170^ ^]^ This process ensures both adequate sorption and good mechanical properties of fibers. The cellulose sources utilized in this process were softwood bleached kraft pulp fibers and nano‐fibrillated cellulose (NFC). These sources, respectively, provide flexibility and tensile strength to the fibers. This method was not only applicable to MIL‐100(Fe) but also to MIL‐53(Al)‐CF_3_, zeolite NaY, and activated carbon. This environmentally friendly and scalable approach offers potential for the industrial application of high‐load porous solid sheets, including in the field of medical devices.

The second method of fabricating porous material fibers involves mixing pre‐synthesized MOFs into a solution prepared for electrospinning. This approach, similar to surface engineering, enhances the stability of MOFs. However, due to limitations in MOF dispersibility, it may be challenging to achieve high loading capacities, and the crystallinity of less stable MOFs might be compromised in the electrospinning solvent. Zhang et al. prepared a titanium carbide (MXene)/ZIF‐8/polylactic acid (PLA) composite membrane, denoted MZ‐8/PLA, through electrospinning (Figure 10c).^[^ ^177^ ^]^ The preparation of the MXene/ZIF‐8/PLA nanofibrous membrane involved several steps including the use of toxic solvents. First, PLA was solved in dichloromethane (DCM). Then MXene/ZIF‐8 (MZ‐8) was added to a solvent mixture of DCM and ethanol, stabilized with polyvinylpyrrolidone (PVP) to ensure homogeneous dispersion. The resulting mixture was then subjected to an electrospinning process to integrate MZ‐8 into the PLA matrix. Under 808 nm laser irradiation, the antibacterial rates of MZ‐8/PLA film reached up to 99.9% and 99.8% against *E. coli* and Methicillin‐resistant *Staphylococcus aureus* (MRSA), respectively. However, there was a notable absence of discussion regarding the loading capacity of MZ‐8 within the membrane. PXRD analysis confirmed the crystallinity of MZ‐8 was decreased. Compared with ZIF‐8, Fe(III)‐based MOFs with enhanced stability may be more suitable for this approach.

The third method entails the direct adsorption or deposition of MOFs onto the surfaces of fibrous films.^[^ ^168^ ^]^ This approach is relatively simple in operation but heavily relies on the adsorption capabilities between the MOFs and the films, which influences the stability. Sun et al. introduced a novel approach utilizing MIL‐101(Fe)‐NH_2_ for the in situ visual detection of TC pollution in water.^[^ ^171^ ^]^ The MIL‐101(Fe)‐NH_2_ derivatives demonstrated outstanding catalytic properties, particularly after calcination at 350 °C, which enhanced the availability of active sites for interacting with TC. Initially, they employed an inkjet printer to fabricate colorimetric paper strips, subsequently dispersing MIL‐101(Fe)‐NH_2_ in a polyvinyl alcohol solution. The mixture was uniformly applied to the hydrophilic areas of the paper to prepare the composites. The resulting paper chip, optimized for TC detection, exhibited a low detection limit of 17.11 nmol L^−1^ and demonstrated high recovery rates (90.6–111.4%) in various water systems, including reclaimed water and aquaculture wastewater. However, the MOFs encapsulated on the paper chip were not quantified in their study. Based on a similar preparation method, Fu et al. introduced a Fe‐MIL‐101@flexible fiber composite, synthesized through in situ cross‐linking on carboxymethyl cellulose substrates, for low‐energy, point‐of‐use water disinfection. The composite demonstrated exceptional stability, maintaining its structural and mechanical integrity even after usage, and exhibited high catalytic activity producing ROS, achieving ≈99% bactericidal efficacy.^[^ ^169^ ^]^ In another study, Zhang et al. developed a novel bactericidal wound dressing made from an electrospun PCL/gelatin/glucose composite fiber mesh incorporated with Fe‐MOF@GOx, designed to autocatalytically generate hydroxyl radicals from glucose to eradicate bacteria.^[^ ^168^ ^]^ The high porosity and mechanical strength of the electrospun fiber mesh enhanced its utility as a wound dressing by facilitating material and gas exchange, promoting cell proliferation, and mimicking the ECM. Their dressing exhibited effective antibacterial activity against MRSA and *E. coli*, improved wound healing in an infected skin wound model in mice, and demonstrated biosafety comparable to commercial silver‐containing dressings. However, the safety of silver‐containing dressings has been questioned due to the risk of Ag ion initial burst release, raising concerns about their long‐term use in clinical settings.

In addition to electrospinning, Mela et al. employed melt electrowriting (MEW) to fabricate fibers with uniform diameters and smooth surfaces, rendering them suitable for use as scaffolds in tissue engineering applications.^[^ ^172^ ^]^ In their recent study, they prepared multifunctional tissue engineering scaffolds by integrating MEW with Fe‐based, silver‐decorated MOFs.^[^ ^172^ ^]^ The incorporation of silver endowed the scaffolds with antibacterial properties, which were essential for preventing infection post‐implantation. Concurrently, the iron component enhanced the visibility of the scaffolds on MRI, which was essential for the non‐invasive monitoring of implants within the body. This shaping strategy not only enriched the functionality of the scaffolds but also broadened their clinical applicability in tissue engineering. It exemplified the potential of MOFs to introduce additional, targeted functionalities into complex scaffold structures, thereby addressing specific clinical needs. The integration of advanced materials fabrication techniques with novel functional materials, such as MOFs, could have a significant impact on the development of next‐generation biomaterials.

#### 3.4.2 Hydrogels

Hydrogel‐based drug delivery platforms offer the sustained maintenance of therapeutic drug concentrations over extended periods, while also reducing the toxicity and side effects of drugs on the body. The development of hydrogels for use in wound dressings or injections can decrease the frequency of administration in clinical settings. Notably, hydrogels responsive to temperature, light, specific enzymes, or pH demonstrate significant potential for targeted stimuli‐responsive therapeutic applications. The high water content, tissue mimicry, and biocompatibility of hydrogels facilitate the in vivo delivery of MOFs and enhance the targeting, chemical, and colloidal stability of MOFs.^[^ ^178^, ^179^ ^]^ Furthermore, hydrogels incorporating MOFs typically exhibit improved mechanical properties and enhanced drug‐loading capacities compared to bare hydrogels. The adaptability of MOFs permits their structures to be customized according to the shape, size, and chemical functionality of drug molecules, effectively complementing existing liposome‐based hydrogel drug delivery systems with less tunability.^[^ ^179^, ^180^ ^]^

Li et al. reported a novel MOF‐thermogel platform that used the drug encapsulation capabilities of both thermogels and MOF components to achieve differentiated dual drug release.^[^ ^179^ ^]^ They utilized Pluronic F127 and a multiblock polyurethane copolymer (termed EPC) as the thermogel polymers to shape MOFs including UiO‐66, MOF‐808, MIL‐100(Fe), and MIL‐53(Al), to assess the impact of these hydrogel@MOF composites on drug release dynamics. They found that in the presence of hydrophilic drugs or drugs with poor hydrophobic–hydrophilic matching with MOFs, the release kinetics were predominantly governed by the hydrogels. This was attributed to the greater affinity of these drugs to hydrogels. Conversely, in the presence of hydrophobic drugs and MOFs with hydrophobic pores, the release kinetics were dominated by the MOFs, due to the greater affinity of hydrophobic drugs to MOFs’ hydrophobic pores. They also found that the UiO‐66 composites offer significant potential for designing differentiated drug release platforms aimed at synergistic multi‐drug therapies. However, the preparation of EPC hydrogels was not feasible with MIL‐100(Fe), likely because the unsaturated Fe sites in MIL‐100(Fe) acted as effective Lewis acid catalysts for the hydrolysis of ester bonds in EPC. This indicates that during the fabrication of Fe‐MOFs into hydrogels, attention must be paid to the chemical structure of the polymers to prevent hydrolytic degradation of the hydrogel materials. In another study, Tian et al. introduced a novel Fe‐Cu MOF/GOx‐PAM hydrogel wound dressing that synergistically enhanced wound healing through improved antibacterial activity and inflammation modulation.^[^ ^173^ ^]^ The hydrogel exhibited superior mechanical adaptability, achieving a stretchability of 230% and a high compressive strength of 802 N, which ensured that the dressing maintained its integrity and functionality under physical stress. Furthermore, the swelling properties of the PAM gel, which were crucial for maintaining a moist wound environment conducive to healing, were preserved in the composites. Moreover, the incorporation of Fe/Cu MOF within the gel significantly enhanced catalytic efficiency, approximately fivefold higher than that of its monometallic counterparts. This enhancement facilitated the rapid production of gluconic acid and H_2_O_2_ from glucose via GOx, augmenting the peroxidase‐like activity of the MOF for an effective antibacterial effect.

#### 3.4.3 Microneedles

The advent of MN technology has revolutionized local therapy by offering a pain‐free, minimally invasive alternative to traditional methods of drug delivery. This innovative approach allows for the precise control of dosage and targeted administration directly into the dermal layer, enhancing both the efficacy and acceptability of treatments.^[^ ^181^ ^]^ A variety of soluble MN systems, composed of biocompatible and biodegradable materials, have been developed for the delivery of therapeutic substances. These systems, which can carry nanoparticles, chemotherapeutic drugs, antimicrobials, genes, immunological agents, photothermal, and photosensitizing agents, demonstrate their unique advantages in applications such as wound care, scar revision, and the treatment of superficial tumors. However, the bioactivities of certain biological agents, such as antimicrobial peptides and vascular endothelial growth factor (VEGF), are readily deactivated so the active ingredients loaded are often limited in variety.^[^ ^182^ ^]^ The diverse composition and drug‐loading capabilities of MOFs offer renewed potential for the development of MNs. These hybrid nanocarriers can stabilize sensitive biological agents and enable the incorporation of a broader range of therapeutic molecules, thus enhancing the therapeutic efficacy and scope of MN‐based treatments. This could dramatically improve the performance of MNs in clinical applications, particularly in the efficient and targeted delivery of bioactive compounds for wound healing and other therapeutic areas. In a recent example, Chen et al. developed a novel therapeutic strategy for treating malignant melanoma, utilizing MN‐assisted transdermal delivery of 2D bimetallic Cu/Fe‐MOF nanosheets (Cu‐TCPP(Fe)@GOx) designed to catalyze cascade reactions within the tumor microenvironment.^[^ ^174^ ^]^ These MN, crafted from dissolvable PVP, ensured the precise and controlled delivery of the catalytic nanosheets directly into melanoma sites, effectively bypassing skin barriers and minimizing systemic side effects. When MNs started to deliver, the Cu‐TCPP(Fe)@GOx nanosheets exploited the acidic conditions prevalent in the tumor environment to convert glucose into H_2_O_2_ and toxic hydroxyl radicals through the Fenton reaction, effectively starving the tumor and inducing tumor cell death.

## 4 Biomedical Applications of Fe‐MOFs–Based Composites

### 4.1 Biosensing

Fe‐MOFs are gaining potential in developing biosensors, where they are typically used as nanoenzymes, signal amplifiers, or carriers of bioactive agents. These applications of Fe‐MOFs improve the stability, selectivity, and sensitivity of sensors by exploiting the multifunctionality and tunability of Fe‐MOFs. In recent years, Fe‐MOFs have been developed for use in optical sensors, such as colorimetric^[^ ^171^, ^183^, ^184^, ^185^, ^186^ ^]^ and fluorescent sensors,^[^ ^187^, ^188^, ^189^, ^190^ ^]^ as well as electrochemical sensors.^[^ ^149^, ^191^, ^192^, ^193^, ^194^ ^]^ These applications take advantage of the unique porous structure of Fe‐MOFs, which can facilitate the rapid diffusion of analytes, thus improving the responsiveness and detection limits of the sensor. Additionally, the variable oxidation states and coordination environments of iron within these frameworks significantly bolster their catalytic efficiency in biosensing applications. There are also innovative studies exploiting the unique magnetic properties of Fe‐MOFs to develop magnetic sensors.^[^ ^195^ ^]^ The sensor utilizes the magnetic responsiveness of the Fe‐MOFs derivative to achieve magnetic separation and self‐calibration to improve the sensitivity and accuracy of the sensor.^[^ ^195^ ^]^ Moreover, Fe‐MOFs serve as excellent substrates for designing hybrid or multimodal sensors.^[^ ^196^ ^]^ These advanced sensors combine different sensing modalities in a single platform, enhancing their ability to detect multiple analytes simultaneously or to validate sensing results through multiple pathways, thereby increasing reliability and accuracy. **Table** **5** provides a summary of recent advances in Fe‐MOFs–based composites in the field of biosensing, highlighting the strategies employed.

**Table 5:** Recent advances in Fe‐MOFs–based composites in biosensing.

Fe‐MOFs	Type	Bioactive agent	Detection Limit (target)	Ref.
MIL‐53(Fe)	chemiluminescence sensor	AuNPs	4.7 × 10^−15^ m (thrombin (THR))	[197]
Fe_7_Ni_3_ MOF	colorimetric sensor	xanthine oxidase (XOD)	1.39 µm (hypoxanthine)	[183]
Ce/Fe‐BDC	colorimetric sensor		1.3 µm (total antioxidant capacity)	[184]
Fe/Co‐BDC‐NH_2_	colorimetric sensor	catalyzed hairpin assembly (CHA)	6.44 pg mL^−1^ (aflatoxin B_1_)	[185]
MIL‐101(Fe)‐NH_2_	colorimetric sensor		17.11 nmol L^−1^ (TC)	[171]
PCN‐222(Fe)	colorimetric sensor	Tetramethylbenzidine (TMB)	0.4 µm (glucose)	[186]
Fe_3_O_4_@MIL‐100(Fe)@Pt/Pd	lateral flow assay sensor	HCR‐multi‐Apt, vancomycin	2 CFU mL^−1^ (*S. aureus*)	[102]
MIL‐101(Fe) derivative	fluorescent sensor	single‐stranded DNA	3.41 nm (acetamiprid)	[187]
Zn/Fe MOF@carbon‐based nanosheets	ratiometric fluorescence sensor		0.12 U L^−1^ (alkaline phosphatase) and 0.59 U L^−1^ (ascorbate oxidase)	[188]
Zn‐TCPP(Fe)	ratiometric fluorescence sensor		39.98 nm (sarcosine)	[189]
MIL‐53(Fe)	ratiometric fluorescence sensor	Tb^3+^, guanosine monophosphate (GMP)	0.18 µm (H_2_O_2_) and 0.73 U L^−1^ (acid phosphatase)	[190]
Pd/Cu‐TCPP(Fe)	electrochemical impedance biosensor	T1R1	0.86 pg mL^−1^ (umami substances)	[191]
Au/Fe‐MIL‐88	electrochemical sensor	Aptamer (Apt), bacterial imprinted film (BIF), methylene blue (MB)	1 CFU mL^−1^ (*S. aureus*)	[103]
Fe‐BDC‐NH_2_	electrochemical/colorimetric sensor		3 cells mL^−1^ (HepG2 cell)	[149]
MIL‐53(Fe)	electrochemiluminescence immunosensor	Ru(bpy)_3_^2+^/PEI	4.04 fg mL^−1^ (embryonic antigen)	[198]
Fe/Pt/Rh MOF	electrochemiluminescence immunosensor	primary antibodies (Ab1), secondary antibodies (Ab2)	6.8 pg mL^−1^ (heart‐type fatty acid‐binding protein)	[192]
Fe/Zn‐MOFs	electrochemiluminescence immunosensor	AuNPs, luminol	0.031 pg mL^−1^ (carcinoembryonic antigen)	[193]
Fe‐BDC‐NH_2_	ratiometric electrochemical immunosensor	AuNPs	0.0166 pg mL^−1^ (deoxynivalenol)	[194]
2D Co/Fe MOF	ratiometric electrochemical immunosensor	Ab2	0.34 pg mL^−1^ (alpha‐fetoprotein)	[199]
MIL‐101(Fe)‐NH_2_	photoelectrochemical immunosensor	Ab2	3 fg mL^−1^ (prostate‐specific antigen)	[200]
Fe_2_O_3_@FeS_2_ derived from MIL‐88A	magnetic photoelectrochemical sensor		2.1 pg mL^−1^ (amyloid‐β 42) and 7.9 pg mL^−1^ (microtubule‐associated protein, Tau)	[195]
Fe/Co‐MOF@Pt	fluorescent/colorimetric/surface‐enhanced Raman spectroscopy triple‐readout sensor		0.05 ng mL^−1^ (sarafloxacin)	[196]
MIL‐101(Fe)‐NH_2_	machine learning‐assisted colorimetric sensor		0.009 nm (transglutaminase 2)	[141]

Fe‐MOFs have emerged as catalysts for chemiluminescence (CL) reactions, opening up new possibilities in biomedical diagnostics. Cao et al. exploited this property to develop an innovative detection system incorporating Au/MIL‐53(Fe) and aptamer‐functionalized luminol within porous silica microspheres.^[^ ^197^ ^]^ This system was specifically designed to target THR with exceptional specificity and sensitivity. By utilizing the base complementary pairing mechanism for assembly, the Fe‐MOFs enhance the chemiluminescence reactions, achieving a lower detection limit of 4.7 × 10^−15^ m and a dynamic range of 1.5 × 10^−14^ to 3.5 × 10^−10^ m. This approach not only demonstrates the robust catalytic capabilities of Fe‐MOFs but also significantly improves the detection capabilities in complex biological samples such as serum.

Colorimetric sensors, which operate on the principle of detecting color changes in response to chemical interactions, benefit greatly from the integration of Fe‐MOFs. These latter act as nanozymes with peroxidase‐mimetic activities, catalyzing chromogenic reactions in the presence of peroxidase substrates, causing rapid and visible color changes, thereby increasing the sensitivity and versatility of colorimetric sensors. For example, Li et al. exploited these properties in a XOD/Fe_7_Ni_3_MOF enzymatic cascade system that responded linearly to hypoxanthine concentrations ranging from 3 to 70 µm.^[^ ^183^ ^]^ The system achieved a remarkably low detection limit of 1.39 µm. The established method has the potential to serve as a crucial foundation for the timely and efficient identification of freshness alterations.

Fluorescent sensors are widely recognized for their high sensitivity and specificity in detecting various analytes through the emission of light by fluorescent molecules when excited. These sensors work by absorbing light at one wavelength and emitting light at another, typically longer, wavelength. Among fluorescence sensors, ratiometric fluorescence sensors stand out for their ability to measure the ratio of fluorescence at two different wavelengths, providing a built‐in correction for environmental variations, such as fluctuations in light intensity or probe concentration, thereby improving the accuracy and reliability of the measurement. In recent research on Fe‐MOFs, Han et al. synthesized 2D iron‐doped carbon‐based nanosheets (Fe‐N800 CS) with catalase‐like activity.^[^ ^188^ ^]^ These nanosheets efficiently generated ROS that oxidized o‐phenylenediamine to 2,3‐diamino phenazine, a compound known for its stable and distinct fluorescence. Taking advantage of this property, they developed a ratiometric fluorescence biosensing platform capable of measuring the activities of enzymes such as alkaline phosphatase and ascorbate oxidase in human serum samples. This platform exemplifies the advantage of Fe‐MOFs in enhancing the reliability of fluorescence signals, making it highly suitable for complex biomedical diagnostics.

**Figure 11:**
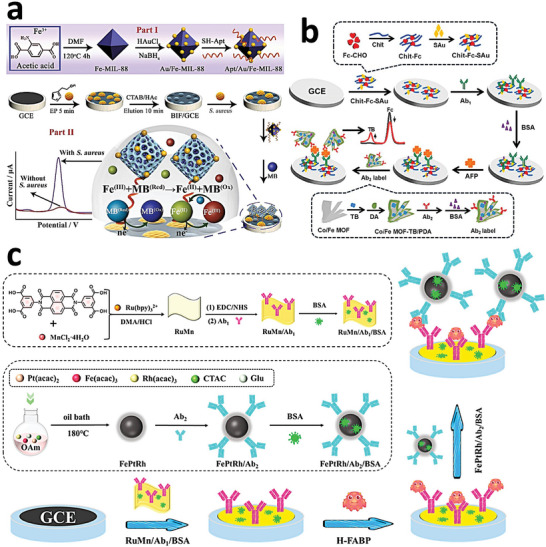
a) Schematic diagram of (Part I) the synthesis of Apt‐Au@Fe‐MIL‐88 bioconjugates and (Part II) their use in constructing a sandwich biosensor for *S. aureus* detection on a BIF‐modified electrode. Reproduced with permission.^[^ ^103^ ^]^ Copyright 2024, ScienceDirect. b) Schematic diagram of the construction of a ratiometric electrochemical immunosensor employing Co/Fe MOF‐TB/PDA and Chit‐Fc‐SAu for AFP detection: Chit‐Fc‐SAu was assembled onto the GCE and dried. Ab1 was applied and incubated to form covalent bonds with the gold surface. BSA was then used to block non‐specific binding sites. The electrode was subsequently incubated with AFP solution followed by incubation with Ab2. Reproduced with permission.^[^ ^199^ ^]^ Copyright 2024, ScienceDirect. c) Schematic diagram detailing the fabrication of (top) RuMn MOFs and (middle) FePtRh MOFs, along with the (bottom) preparation route of the ECL immunosensor. Reproduced with permission.^[^ ^192^ ^]^ Copyright 2024, ACS.

Electrochemical sensors are characterized by their ability to convert a chemical reaction into an electrical signal. This class of sensors is highly valued for its rapid response, high sensitivity, and low cost of ownership. They are particularly suited for continuous monitoring and on‐site analysis, which are critical for the food industry and clinical applications. Liu et al. developed an innovative electrochemical sandwich biosensor for rapid and sensitive detection of *S. aureus* in the food industry.^[^ ^103^ ^]^ This biosensor utilized a dual receptor system that combined a BIF and aptamers to enhance targeted bacterial capture and signal generation. Upon detection of *S. aureus* in a sample, the biosensor formed a sandwich structure between the BIF and Apt‐Au@MOF mediated by the captured bacteria. MB was then incubated with this structure, where it was loaded by Fe‐MOFs to enhance the characteristic peak current in a [Fe(CN)_6_]^3−/4−^ solution. This enhancement was due to the redox recycling of Fe(II) facilitated by MB, which significantly increased the anodic current of [Fe(CN)_6_]^3‐/4^ through a dual process: electro‐oxidation of Fe(II) to Fe(III) and chemical reduction of Fe(III) by MB (**Figure** **11**a). The intensity of this current was proportional to the amount of MB loaded and directly correlated with the concentration of *S. aureus* in the sample. This biosensor demonstrated exceptional analytical performance with a LOD of 1 CFU mL^−1^, a wide detection range of 10 to 10^8^ CFU mL^−1^, and high selectivity. Taking advantage of the MB‐loading capacity of Fe‐MOFs and its effective redox cycling, this method offers the potential for the detection of bacteria in complex samples, especially for food safety and public health. However, the biosensor's fabrication involves a complex multi‐step process that includes electropolymerization of the BIF, aptamer modification of MOFs, and careful assembly of the sandwich structure. These steps require precise control and can extend the preparation time, potentially limiting rapid deployment in field conditions. Additionally, the use of noble metals like gold in the synthesis of composites can drive up production costs, making the technology less accessible for routine application in resource‐limited settings. Recently, Kong et al. developed an electrochemical biosensor by integrating Pd‐modified 2D bimetallic MOFs with T1R1 recognition elements.^[^ ^191^ ^]^ Utilizing the peroxidase‐like activity, this biosensor significantly amplifies the impedance signals, enabling the sensitive detection of umami substances. When tested on real food samples, this sensor demonstrated its potential for advanced flavor assessment and grading. This study highlights the simple strategy of bimetallic MOFs in enhancing the performance of electrochemical sensors. In another study related to bimetallic MOFs, Sun et al. employed the substantial surface area, synergistic and electronic effects of 2D Co/Fe MOF, and the abundant functional groups of PDA, to develop a Co/Fe MOF‐toluidine blue (TB)/PDA system (Figure 11b).^[^ ^199^ ^]^ This system permitted the increased loading of Ab2 and signaling molecules (TB), thereby providing an amplified electrochemical signal. This enhancement significantly improves the accuracy of alpha‐fetoprotein (AFP) detection in clinical diagnoses, thereby demonstrating the potential of bimetallic MOFs to enhance the performance of biosensors in healthcare applications.

Considering electrochemiluminescence (ECL) sensors, Fe‐MOFs–based composites not only act as support materials for active agents but also serve as co‐reaction promoters, enhancing the luminescence efficiency of systems. For instance, Jiang et al. demonstrated that incorporating MIL‐53(Fe) significantly amplified the ECL signal intensity, enabling the detection of carcinoembryonic antigen at extremely low concentration ranges.^[^ ^198^ ^]^ In a recent study, Li et al. developed an ECL quenching system using multimetallic–organic frameworks (MMOFs) for the detection of heart‐type fatty acid‐binding protein (H‐FABP), a biomarker for acute myocardial infarction (AMI).^[^ ^192^ ^]^ As shown in Figure 11c, They crafted bimetallic MOFs with ruthenium and manganese centers to function as robust ECL emitters, while a trimetal‐based MOF based on iron, platinum, and rhodium (Fe/Pt/Rh MOF) served as an efficient ECL quencher, leveraging enhanced electron transfer properties. This novel system demonstrated a wide detection range from 0.01 to 100 ng mL^−1^ and an impressively low detection limit of 6.8 pg mL^−1^ for H‐FABP. Its high specificity and reproducibility make MMOFs a promising tool for AMI diagnosis in clinical settings. Moreover, the cooperation of two different MOFs within these systems significantly enhances the applicability of biosensors and illustrates the potential for tailored diagnostic approaches. In another study combining two MOFs, Cheng et al. developed a photoelectrochemical immunosensor by employing MOF‐525 as the photoanode material and peroxidase‐mimetic MIL‐101(Fe)‐NH_2_ as a signal amplification tag.^[^ ^200^ ^]^ The high cathodic photoelectrochemical activity of MOF‐525 combined with the signal amplification from MIL‐101(Fe)‐NH_2_ allowed for the ultra‐sensitive detection of prostate‐specific antigen. This sensor achieved a remarkably low detection limit of 3 fg mL^−1^. This development underscores the effectiveness of utilizing coordinated functions of different MOFs to enhance the performance and sensitivity of biosensors, paving the way for more precise and reliable disease detection in clinical practice.

Fe‐MOFs have recently been used to improve the efficacy of biosensing by combining promising machine‐learning techniques. Supianto et al. introduced a colorimetric LFA using MIL‐101(Fe) to detect transglutaminase 2 (TGM2), a biomarker for chronic kidney disease (CKD).^[^ ^141^ ^]^ Machine learning techniques were utilized to precisely quantify TGM2 concentrations by analyzing immunoreaction sites. This methodological enhancement not only improved the assay's sensitivity by 55 times compared to conventional methods but also ensured rapid and accurate point‐of‐care testing suitable for clinical diagnostics. This integration signified a major step forward in the development of more efficient, sensitive, and rapid diagnostic platforms for CKD and possibly other diseases.

### 4.2 Anticancer

**Figure 12:**
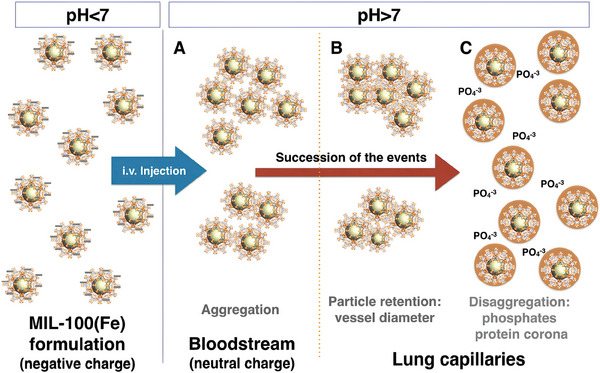
Scheme of passive targeting of MIL‐100(Fe) nanoparticles to address lung cancer. A) After intravenous administration blood pH causes early nanoMOF aggregation. B) Particle aggregates are rapidly retained in the lung capillaries due to the small vessel diameter. C) Progressive disaggregation is the result of the combination of various factors such as phosphate coordination to the iron sites and protein adsorption on the surface of the nanoparticles. Reproduced with permission.^[^ ^201^ ^]^ Copyright 2017, Wiley.

Anticancer treatment is still facing significant limitations, including non‐specificity, systemic toxicity, and the emergence of resistance in traditional therapeutic approaches. Nanotechnology, particularly through the development of drug carriers, offers promising new avenues to address these challenges. In 2017, Simon Yarza et al. employed passive targeting of bare MIL‐100(Fe) nanoparticles to address lung cancer (**Figure** **12**), taking advantage of the aggregation/degradation of MOF nanoparticles into the lung blood capillaries, pioneering the first in vivo utilization of Fe‐MOFs for anticancer treatment.^[^ ^201^ ^]^ Subsequently, Fe‐MOF–based composites are at the forefront of advancing multimodal treatment methods and enhancing imaging capabilities, providing a comprehensive strategy to combat cancer effectively.

Recently, Fe‐MOF–based composites have shown promises in the field of oncology by providing a platform that integrates various therapeutic modalities, including DDS,^[^ ^151^, ^202^, ^203^, ^204^ ^]^ chemotherapy,^[^ ^150^, ^205^, ^206^ ^]^ CDT,^[^ ^207^, ^208^, ^209^ ^]^ immunotherapy,^[^ ^138^, ^210^ ^]^ PDT,^[^ ^97^, ^211^, ^212^, ^213^ ^]^ PTT,^[^ ^97^, ^212^, ^213^ ^]^ SDT,^[^ ^210^, ^214^, ^215^ ^]^ and microwave thermal therapy.^[^ ^64^ ^]^ **Table** **6** provides a summary of recent advances in Fe‐MOFs–based composites in the field of anticancer treatment, highlighting the strategies and in vitro/in vivo models employed in their study.

**Table 6:** Recent advances in Fe‐MOFs–based composites in anticancer treatment.

Fe‐MOFs	Strategy	Bioactive agent	Particle size [nm]	Zeta potential [mV]	In vitro/In vivo model	Route	Ref.
MIL‐101(Fe)‐NH_2_	DDS	luteolin (LC = 9.8 wt%), matrine (LC = 14.1 wt%)	395	12.7	RKO and FHC cells		[216]
Fe/Ni‐BDC‐NH_2_	DDS	silymarin (Syl) (EE = 68%)	10–20		MCF‐7, SHYSY5Y, and HUVEC cells		[202]
MIL‐101(Fe)	DDS	irinotecan	200–300	11.4	MCF‐7 and HCT116 cells		[203]
MIL‐88B(Fe)‐NH_2_	DDS	alpha‐tocopheryl succinate (LC = 9 wt%), curcumin (LC = 42 wt%, EE = 73%)	60–70	−10	HeLa cells		[217]
Fe‐BDC‐NH_2_	DDS	larotrectinib (LC = 10 wt%)	500		4T1 tumor‐bearing mice	oral	[204]
Co_8_FeS_8_@Co_1‐x_S	CDT		>1000		mice by axillary injection of H22 cancer cells	intratumoral injection	[208]
MIL‐53(Fe)‐NH_2_	CDT, chemotherapy, PDT	upconversion nanoparticles (UCNPs), Doxorubicin (DOX) (LC = 7 wt%)	20	20	GL261 tumor‐bearing mice	intravenous injection	[218]
MIL‐101(Fe)	CDT, chemotherapy, PTT	Cu‐TA, camptothecin (CPT) (LC = 24 wt%)	118	−10.6	HepG2 cells		[150]
SMn*_x_* Fe*_y_* H MOF	CDT, chemotherapy, starvation therapy	AuNPs, DSCP, syringic acid (LC = 6 wt %, EE = 60%)	64	−5	B16F10 tumor‐bearing mice	intravenous injection	[133]
MIL‐100(Fe)	CDT, DDS	arginine–glycine–aspartic acid	500		KLE tumor‐bearing mice	intratumoral injection	[209]
Fe/Mn‐TCPP	CDT, PDT		196	−17	CT‐26 tumor‐bearing mice	intratumoral injection	[211]
MIL‐53(Fe)	ferroptosis	atorvastatin (LC = 9 wt%)	65	close to 0	mice by axillary injection of CT‐26 cancer cells	intravenous injection	[140]
MIL‐101(Fe)	ferroptosis	Pt	100–200		4T1 tumor‐bearing mice	intratumoral injection	[219]
MIL‐100(Fe)	ferroptosis, chemotherapy	oxaliplatin (LC = 27 wt%)	278		mice by injection of HGC‐27 cells into the right buttock	tail vein injection	[220]
MIL‐101(Fe)	ferroptosis, chemotherapy, immunotherapy, PDT	copper nanoparticles (CuNPs), cisplatin (LC = 40.5 wt%), 1‐methyl‐d‐tryptophan (1‐MT) (LC = 9.5 wt%), TCPP, CaO_2_	150	−30	mice by axillary injection of H22 cancer cells	tail vein injection	[138]
MIL‐53(Fe)	ferroptosis, chemotherapy, immunotherapy, SDT	CM‐272 (LC = 56.4wt%)	100	6.3	mice by subcutaneous injection of MB49 cells	tail vein injection	[210]
MIL‐101(Fe)‐NH_2_	ferroptosis, chemotherapy, starvation therapy	Pt(IV) prodrug (LC = 11 wt%), GOx (LC = 20 wt%)	200	−15	4T1 tumor‐bearing mice	intratumoral injection	[205]
MIL‐100(Fe)	DDS, hyperthermia treatment, MRI	FeAu NP, DOX	16	−19	mice by injection of HSC‐3 cells into the right forelimb		[221]
Gd/Fe MOF	DDS, microwave thermal therapy, MRI	Lenvatinib (LC = 15 wt%)	150	−1.8	H22 tumor‐bearing mice	intravenous injection	[64]
Fe/ZIF‐8	DDS, PDT, PTT, bioimaging	IR820 (EE = 13%), GOx (EE = 2%), tilazamine (EE = 5%)	190	−20	4T1 tumor‐bearing mice	tail vein injection	[212]
MIL‐100(Fe)	DDS, PL/MR dual‐modal imaging	DOX (LC = 87 wt%), PL NP	48	−6.7	4T1 tumor‐bearing mice	intratumoral injection	[98]
MIL‐88B(Fe)‐NH_2_	CDT, DDS, bioimaging	MG132 (LC = 48 wt%)	250	−30	CT‐26 tumor‐bearing mice	tail vein injection	[154]
Co‐Fc MOF	CDT, DDS, bioimaging	vitamin k3 (LC = 12 wt%)	150	−8.8	4T1 tumor‐bearing mice	intravenous injection	[222]
Fe/ZIF‐8	CDT, DDS, gas therapy, bioimaging	l‐arginine, DOX (LC = 7 wt%)	170	−21	4T1 tumor‐bearing mice	tail vein injection	[144]
Fe‐2‐MeIm	CDT, MRI	DOX (EE = 20%)	400	17	4T1 tumor‐bearing mice	intravenous injection	[223]
Fe‐BDC	ferroptosis, apoptosis, bioimaging	DOX (EE = 13%)	195	−18.5	4T1 tumor‐bearing mice	intravenous injection	[224]
HM@Fe‐BTC	ferroptosis, chemotherapy, bioimaging	DOX (LC = 12 wt%)	150		4T1 tumor‐bearing mice	intratumoral injection	[225]
Fe‐BDC‐NH_2_	ferroptosis, chemotherapy, bioimaging	buthionine sulfoxide amine (BSO), oxaliplatin	150	15	4T1 tumor‐bearing mice	intravenous injection	[151]
Fe‐2‐MIM	ferroptosis, chemotherapy, bioimaging	DOX	142	−21.5	A549 tumor‐bearing mice	intravenous injection	[155]
TA‐Fe^3+^MOF	ferroptosis, immunotherapy, bioimaging	triptolide (TPL) (LC = 36 wt%, EE = 81%)	200	−17	B16F10 tumor‐bearing mice	tail vein injection	[143]
MIL‐101(Fe)‐NH_2_	ferroptosis, immunotherapy, bioimaging	dihydroartemisinin (DHA)	150–200	−30	mice by subcutaneous injection of Lewis lung cancer cells	intravenous injection	[226]
Fe‐TCPP	ferroptosis, chemotherapy, PDT, bioimaging	Cu^2+^, disulfiram (DF) (LC = 9 wt%, EE = 39%), tannic acid	150	−38.6	MDA‐MB‐231 tumor‐bearing mice	intravenous injection	[206]
Fe‐TCPP	ferroptosis, chemotherapy, PDT, bioimaging	oxaliplatin (LC = 3.2 wt%)	164	−30	4T1 tumor‐bearing mice	intravenous injection	[157]
MIL‐101(Fe)‐NH_2_	ferroptosis, chemotherapy, SDT, bioimaging	gemcitabine (GEM) (LC = 20 wt%, EE = 77%)	250	9	PANC‐1/GEM tumor‐bearing mice	intravenous injection	[214]
MIL‐100(Fe)	DDS, radiotherapy, chemotherapy	GEM monophosphate (LC = 10 wt%, EE >98%)	220		Hela cells		[227]
MIL‐88B(Fe)‐NH_2_	ferroptosis, chemotherapy, SDT, bioimaging	paclitaxel (PTX) (EE = 86%), chlorin e6 (Ce6) (EE = 98%)	400		4T1 tumor‐bearing mice	intratumoral injection	[215]
PCN‐224(Fe)	ferroptosis, chemotherapy, SDT, near‐infrared (NIR)‐IIb imaging	sorafenib (LC = 72 wt%), DSNP	108	−12.4	mice by injection of C6‐Luc cells into the right brain	intravenous injection	[228]
MIL‐88B(Fe)‐NH_2_	ferroptosis, cuproptosis, bioimaging	Cu*_x_* OS	164	20	mice by injection of 4T1 cells into the left flank	intravenous injection	[229]
FcMOF	ferroptosis, mild magnetic hyperthermia therapy, bioimaging		156	−23	mice by injection of H22 cancer cells into the hind leg	intratumoral injection	[128]
MIL‐100(Fe)	ferroptosis, PDT, PTT, bioimaging	PtNP, iodinated‐cyanine dye (LC = 22 wt%, EE = 90%)	150	−24	mice by axillary injection of CT‐26 cells	intravenous injection	[97]
PCN‐600	PDT, PTT, MRI	DOX (LC = 78 wt%)	180	close to 0	4T1 tumor‐bearing mice	intratumoral injection	[213]
MIL‐101(Fe)	radiotherapy, single‐photon emission computed tomography (SPECT), fluorescence imaging (FI)	^177^Lu	166	14.8	U87MG tumor‐bearing mice	intravenous and intratumor injection	[147]

LC: loading capacity; EE: Encapsulation efficiency.

#### 4.2.1 DDS and Chemotherapy

Fe‐MOF–based composites optimize the delivery of chemotherapeutic agents, significantly reducing systemic toxicity while enhancing tumor‐specific accumulation. This selective delivery helps to mitigate the common side effects associated with traditional chemotherapy, thereby improving patient outcomes. The range of chemotherapeutic agents is extensive, with specific drugs targeting various types of cancer. For instance, Oxaliplatin is widely used in the treatment of cancer, particularly colorectal cancer. DOX is utilized for treating a multitude of cancers, such as leukemia, lymphoma, breast cancer, and liver cancer. 5‐Fluorouracil is employed in the treatment of colorectal, esophageal, and stomach cancers. GEM is used to treat testicular cancer, breast cancer, ovarian cancer, and more. In recent anticancer research (Table 6), DOX is most used as a model drug in most Fe‐MOFs–based studies. From a chemical structure perspective, the phenolic hydroxyl groups in the DOX molecule can coordinate with ligands or Fe^3+^ sites in trimer‐based carboxylate Fe‐MOFs while the phenyl rings of the drug is expected to interact via dispersive interactions with the ligand of the MOF, also ensuring a strong hydrophobic character of the pores. The formation of these interactions is known to stabilize the loading and strongly influences the release kinetics of DOX.

In a recent study, Li et al. introduced a strategy that employed a Fe‐MOF encapsulating DOX, GOx, and the NO precursor l‐arginine to address tumor drug resistance by integrating chemotherapy, CDT, NO‐based gas therapy, and starvation therapy.^[^ ^144^ ^]^ The Fe‐MOF–based composites catalyzed the degradation of glucose and GSH while generating substantial amounts of ROS and NO. This multifaceted approach disrupted cellular metabolism and redox homeostasis, effectively inhibiting tumor proliferation both in vitro and in vivo. The utilization of drug‐loaded Fe‐MOFs allowed for the simultaneous implementation of ferroptosis and various other therapeutic strategies and potentially reduced the risk of resistance associated with chemotherapy. However, the complexity of coordinating multiple therapeutic modalities could complicate treatment plans, necessitating more precise management and monitoring. This could increase costs and patient burden and impact the accessibility of such therapies. From a research perspective, evaluating the combined effects of multiple treatments is inherently more complex than assessing single‐agent therapies. Further clinical trials and data are required to verify the safety and efficacy of this combined treatment. Li et al. pioneered the use of MIL‐100(Fe) loaded with GEM monophosphate to achieve enhanced radiotherapy and chemotherapy.^[^ ^227^ ^]^ In their study, MIL‐100(Fe) acted as both drug carriers and radiosensitizers, demonstrating a radiotoxicity enhancement factor of 1.8.

In addition to loading chemotherapeutic drugs, Fe‐MOFs are also commonly used to load natural medicines. An innovative nano‐composite carrier, NH_2_‐MIL‐101(Fe)@GO (MG), was developed by Shen et al. to co‐deliver luteolin and marine for enhanced anti‐colorectal cancer therapy.^[^ ^216^ ^]^ The composites successfully addressed luteolin's poor solubility and susceptibility to degradation by gastrointestinal enzymes by exploiting the complexation between the o‐phenol hydroxyl group of luteolin and the iron ions at the metal center, which is pH‐sensitive and breaks in acidic conditions, thereby facilitating targeted release in the acidic tumor microenvironment. The loading capacities achieved for luteolin and marine were 9.8% and 14.1%, respectively. The release of luteolin was significantly higher at pH 5 compared to pH 7.4, indicating effective pH‐responsive release characteristics. In comparison to the individual components, the drug‐loaded MG exhibited superior anticancer activities, including the inhibition of tumor cell migration, the increase in ROS production, and the upregulation of the expression of apoptosis‐inducing proteins Caspase‐3 and Caspase‐9.

#### 4.2.2 CDT and Ferroptosis

CDT and ferroptosis are intricately connected, both involving the catalytic role of Fe ions and the generation of ROS. In the context of CDT, the production of hydroxyl radicals can facilitate the peroxidation of lipids, potentially contributing to ferroptosis. The concepts of CDT and ferroptosis are often conflated. CDT is a therapeutic strategy that primarily utilizes the Fenton reaction to produce hydroxyl radicals, which directly kill cells. Conversely, ferroptosis is a form of cell death triggered by specific treatment strategies, resulting in cell death through lipid peroxidation‐induced loss of cell membrane integrity. This form of cell death may serve as a potential mechanism in cancer therapy. A recent review of Kudarha et al. provides a comprehensive overview of the various therapeutic mechanisms induced by Fe‐MOFs that lead to ferroptosis, and discusses their significance in research.^[^ ^19^ ^]^

Fe ions in Fe‐MOF–based composites can initiate both CDT and ferroptosis. The Fenton reaction, catalyzed by these composites, generates ROS within the tumor microenvironment. This not only triggers cell death through oxidative stress but also promotes ferroptosis, illustrating a synergistic effect that enhances the overall therapeutic efficacy and mitigates drug resistance. The effectiveness of CDT is significantly influenced by the Fenton reactions, typically depends on the interaction between H_2_O_2_ and Fe^2+^. Generally, lowering the reaction potential of Fenton reactions can improve the effect of CDT in the TME. In addition, CDT can be enhanced by remodeling the TME, influencing iron metabolism, providing suitable reaction conditions for Fenton reactions.^[^ ^19^ ^]^ The customizability of Fe‐MOFs makes them adaptable for enhancing CDT by different pathways. Additionally, Fe‐MOFs can facilitate synergistic interactions with drugs such as ferroptosis inducers, effectively triggering ferroptosis.^[^ ^151^ ^]^

**Figure 13:**
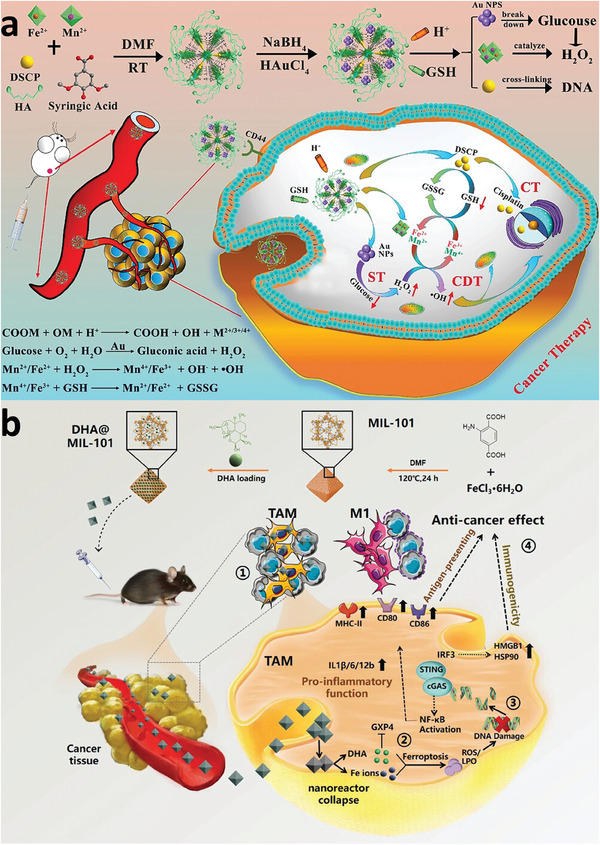
a) Schematic diagram of the synthesis and intracellular working mechanism for the constructed SMnFeCGH. Reproduced with permission.^[^ ^133^ ^]^ Copyright 2023, Wiley. b) Schematic diagram of the DHA nanoreactor‐triggered M1 polarization of TAM through ferroptosis‐driven activation of the cGAS/STING signaling pathway. Reproduced with permission.^[^ ^226^ ^]^ Copyright 2023, Wiley.

Chen et al. emphasized the pivotal role of metal ions in enhancing the efficacy of CDT.^[^ ^133^ ^]^ Their study introduced a sophisticated nanoplatform‐based on pH and GSH‐dual responsiveness Mn/Fe‐based MOFs, SMnFeCGH, engineered for synergistic cancer therapy (**Figure** **13**a). The integration of AuNPs, syringic acid, and cisplatin prodrugs into these MOFs promoted a tripartite mechanism involving CDT, starvation therapy, and chemotherapy. This multimodal approach, bolstered by the selective catalysis of Fe and Mn ions, proved significantly effective in inducing cancer cell death, offering a novel strategy for constructing multifunctional therapeutic systems.

Moreover, cascade nanozyme systems are designed to enhance CDT and ferroptosis. Wang et al demonstrated the dual enzymatic activities of MOF‐derived nanozymes, Co8FeS8@Co1‐xS, including peroxidase‐like and glutathione oxidation functions.^[^ ^208^ ^]^ By inhibiting the anti‐apoptotic protein Bcl‐2, activating caspase proteins, and influencing iron metabolism through genes such as Hmox‐1, they effectively enhanced the Fenton reaction. These composites effectively generated hydroxyl radicals and depleted glutathione, thereby triggering apoptosis and ferroptosis in tumor cells. These properties position Fe‐MOF–based nanozymes as potent tools for targeted cancer therapy.

Immunosuppressive agents and ferroptosis inducers are also used to enhance CDT and ferroptosis with Fe‐MOFs. Wang et al. developed a bi‐modal nanoplatform, TPL@TA‐Fe^3+^@BSA‐FA, aiming at potent anti‐tumor therapy by inducing both ferroptosis and pyroptosis.^[^ ^143^ ^]^ These TPL@TA‐Fe^3+^nanoparticles, functionalized with BSA‐FA, not only facilitated targeted drug delivery but also enhanced iron endocytosis. TPL heightened the production of intracellular ROS by suppressing the expression of nuclear factor erythroid‐2–related factor (Nrf2). Upon cellular uptake, the platform dissociated, leveraging the Fenton reaction to produce ROS and inhibit Nrf2, thus amplifying cell death pathways and triggering a systemic immune response against tumor growth and metastasis. While the results are promising for cancer therapy, it is important to note that if the TA‐Fe^3+^ complex is neither crystalline or nor porous, then it cannot be qualified as a MOF. Furthermore, further research should explore the characteristics of each so‐called “MOF.” These materials should exhibit a 2D or 3D structure and be micro or meso‐porous. The frequent misuse of the “MOF” terminology within the biomedical field highlights the necessity to distinguish between true MOFs and other coordination complexes or amorphous solids. This is of paramount importance for the accurate and precise communication and application of scientific findings.

In another study, Zhang et al. employed a biodegradable zwitterionic polymer, PMPC, to coat MIL‐53(Fe).^[^ ^140^ ^]^ MIL‐53(Fe) was loaded with a ferroptosis inducer, ATV, for ferroptosis‐inducing cancer therapy. The GSH‐triggered release of Fe^2+^and ATV enhanced tumor accumulation and prolonged circulation time. Once internalized by cancer cells, the nanoplatform degraded in response to GSH at the tumor site, depleting GSH and releasing ATV and Fe^2+^. This release disrupted MVA biosynthesis and further depleted GSH, suppressing GPX4 expression. Meanwhile, the liberated Fe^2+^ catalyzed the Fenton reaction, generating high levels of ROS. These processes collectively led to lethal lipid peroxidation, effectively inducing ferroptosis. The nanoplatform demonstrated significant tumor growth inhibition in vivo, with an 86% tumor‐inhibitory ratio. Furthermore, it exhibited excellent biocompatibility, indicating its potential as a promising strategy for cancer therapy. Rao et al. introduced a novel ferroptosis amplification approach by employing BSO to effectively enhance CDT.^[^ ^151^ ^]^ Their strategy involved the dual action of iron‐mediated ROS production via the Fenton reaction and the concurrent inhibition of GSH biosynthesis by BSO. This action resulted in both the inactivation of GPX4 and the accumulation of lipid peroxides, thereby significantly amplifying ferroptotic cell death. In a recent study, Bu et al. introduced a novel epigenetic modulation strategy utilizing MIL‐88B(Fe)‐NH_2_ loaded with the 26S proteasome inhibitor MG132 to enhance CDT to overcome tumor resistance in metastatic colorectal cancer (mCRC).^[^ ^154^ ^]^ The MG132‐mediated ubiquitination blockade was designed to block the phosphorylation of nuclear factor‐κB (NF‐κB) transcription factors in mCRC, thereby silencing the carcinogenic driving genes associated with mCRC. This further magnified the CDT‐induced oxidative stress.

CDT is often combined with chemotherapy to induce ferroptosis. Sun et al. presented a therapeutic strategy leveraging ferroptosis induced by MIL‐100(Fe), alongside chemotherapy induced by loaded oxaliplatin.^[^ ^220^ ^]^ This straightforward Fe‐MOF–based combination demonstrated the potential for integrating ferroptosis into oncological pharmacotherapy. In this context, Qian et al. introduced an HM@FM (hollow mesoporous organosilica nanoparticles@Fe‐MOF) nanosuspension that encapsulated DOX.^[^ ^225^ ^]^ This design leveraged the disulfide bonds for dual‐responsive drug release and employed iron ions from the Fe‐MOF shell to enhance oxidative stress within tumor cells, triggering ferroptosis synergistically with DOX‐induced ROS. Also, their HM@FM is biodegradable in GSH solution. This nanosuspension not only provides sustained chemotherapy but also boosts antitumor immunity by serving as an immunologic adjuvant, demonstrating the significant potential for postoperative cancer treatment and reducing the recurrence and metastasis of breast cancer.

In another study, Luo et al. presented a biomimetic Fe‐based nanoparticles, synthesized by iron sulfate and dimethylimidazole designed to address drug resistance in non‐small cell lung cancer by combining chemotherapy with ferroptosis.^[^ ^155^ ^]^ Further structural characterization of the MOF nanoparticles is necessary to confirm whether they are MOFs or other nanocomplexes. Their “Fe‐MOF,” loaded with DOX, effectively released Fe^2+^ and DOX in the acidic intracellular environment, enhancing ROS generation and promoting GPX4‐mediated ferroptosis alongside DOX‐induced apoptosis. The synergistic effect of ferroptosis and apoptosis significantly curtailed tumor growth and lung metastasis, highlighting the potential of Fe‐MOFs in enhancing the efficacy of lung cancer chemotherapy. However, it is crucial to provide systematic analysis on the quantity of drug loaded within the carrier and the overall formulation. This transparency enables the assessment of the efficiency and potential of the formulation.

CDT and ferroptosis can also be combined with immunotherapy to achieve cancer treatment. In this innovative study, Li et al. introduced the utilization of the DHA@MIL‐101(Fe)‐NH_2_, which reprogramed tumor‐associated macrophages (TAMs) into an anti‐tumor M1 phenotype through ferroptosis.^[^ ^226^ ^]^ The study demonstrated that MIL‐101(Fe)‐NH_2_ enhanced the water solubility and biocompatibility of DHA, significantly boosting its efficacy in TAM reprogramming. As shown in Figure 13b, this reprogramming was mediated by the ferroptosis‐induced DNA damage and subsequent activation of the cGAS/STING signaling pathway, which further stimulated NF‐κb and interferon‐related factor pathways, enhancing the immunogenicity and inflammatory responses of TAMs. The strategy made use of the selective uptake of Fe‐MOFs–based composites by TAMs over malignant cells, exploiting the inherent differences in basal iron levels to induce low‐level ferroptosis in TAMs, thereby promoting an anti‐cancer immune response. This work demonstrated a novel strategy involving ferroptosis for targeted immune modulation in lung cancer therapy.

#### 4.2.3 PDT and PTT

Fe‐MOF–based composites play a pivotal role in both PDT and PTT, where they function as photosensitizers or heat generators to target and destroy cancer cells. Although Fe‐MOFs are colored, they exhibit minimal absorption of IR or near‐IR light, which experiences less scattering and absorption in biological tissues and allows for deeper penetration into the human body, enabling the light to reach deeper tumors or diseased areas. Therefore, it is necessary to couple Fe‐MOFs with phototherapeutic agents that absorb in this region to achieve effective treatment. In PDT, the light‐mediated activation of photosensitizers encapsulated within the Fe‐MOF–based composites produces ROS that can effectively kill cancer cells. This approach can absorb specific wavelengths of light, triggering a chemical reaction that generates cytotoxic species without invasive procedures. Which is particularly advantageous for the treatment of superficial or light‐accessible tumors.

A recent review of Huang et al. exemplifies the potential of PDT combined with ferroptosis as a synergistic antitumor therapy strategy.^[^ ^230^ ^]^

**Figure 14:**
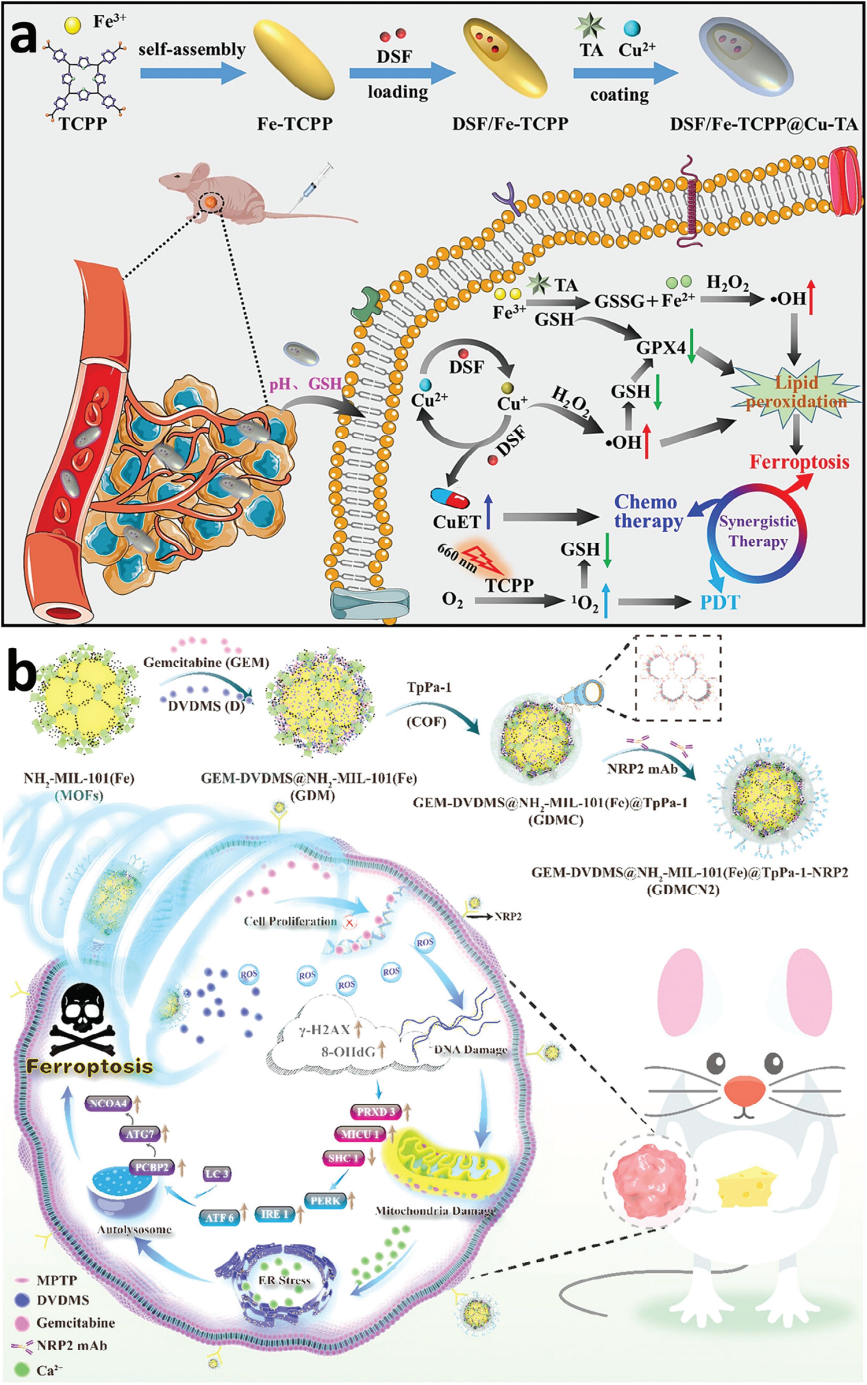
a) Schematic diagram of the preparation of DSF/Fe‐TCPP@Cu‐TA and the proposed mechanism of the synergistic antitumor effect by combining Fe^3+^‐inducing ferroptosis, DSF/Cu^2+^‐dependent chemotherapy/ferroptosis, and TCPP‐participating PDT. Reproduced with permission.^[^ ^206^ ^]^ Copyright 2024, ScienceDirect. b) Schematic diagram of the design of a novel nanocage, GEM‐DVDMS@MOFs@COF‐NRP2 (GDMCN2), which specifically targets human GEM‐resistant cells (PANC‐1/GEM) via pancreatic cancer‐specific targeting. Upon sonodynamic stimulation, GDMCN2 generates a substantial amount of ROS that induces mitochondrial and DNA damage in cells, leading to the activation of ER stress and ultimately triggers autophagy‐dependent ferroptosis. Reproduced with permission.^[^ ^214^ ^]^ Copyright 2023, Wiley.

Among the Fe‐MOF family, some porphyrin‐based MOFs can activate PDT with long‐wavelength light, offering deeper tissue penetration and fewer side effects. Fe‐ TCPP has been extensively utilized in cancer research focusing on PDT and ferroptosis. Meng et al. developed Fe‐TCPP, coated it with a Cu‐TA metal‐phenolic network, and loaded it with DSF to create DSF/Fe‐TCPP@Cu‐TA nanocomposites.^[^ ^206^ ^]^ As demonstrated in **Figure** **14**a, the nanocomposites exploited the acidic tumor microenvironment to facilitate the release of Cu^2+^, Fe^3+^, and TA. Concurrently, GSH reduced Fe^3+^ to Fe^2+^, producing highly toxic hydroxyl radicals via the Fenton reaction. Additionally, Cu^2+^ reacted with DSF to form CuET, enhancing chemotherapy effects. Upon 660 nm laser irradiation, TCPP in Fe‐TCPP generated singlet oxygen, augmenting PDT efficacy. ROS and GSH depletion release drove extensive lipid peroxidation and inhibited GPX4, promoting ferroptotic cell death. The integration of ROS generation, PDT, and chemotherapy resulted in a robust synergistic effect between apoptosis and ferroptosis, leading to significant tumor inhibition in both in vitro and in vivo studies. This strategy highlights the potential of integrating multiple therapeutic modalities to enhance cancer treatment effectiveness. In another study, Zhang et al. introduced a novel multi‐bioinspired MOF delivery system (FeTPt@CCM) using droplet microfluidics to load oxaliplatin and employ homologous CCM camouflage, mimicking a Trojan Horse.^[^ ^157^ ^]^ The FeTPt@CCM system integrated chemotherapy, ferroptosis, and PDT, exhibiting enhanced tumor targeting and therapeutic efficacy. Specifically, their system showed promise in promoting GSH depletion and ROS generation for ferroptosis induction, alongside oxaliplatin release for chemotherapy. Additionally, the TCPP component was activated under a red light for effective PDT. This multifaceted therapeutic approach, capable of inducing immunogenic cell death (ICD), effectively suppresses tumor growth in vivo and potentially stimulates antitumor immunity, paving the way for new integrated treatment methods.

The development of MOF‐based phototherapeutic agents has generated interest due to their structural flexibility and extensive catalytic sites. However, the majority of Fe‐MOFs, with the exception of those based on porphyrins, only utilize UV/visible light and lack responsiveness to near‐infrared light. To improve this, Lv et al. proposed a simple method to integrate UCNPs with MOFs to construct near‐infrared responsive composite photocatalysts for glioblastoma treatment.^[^ ^218^ ^]^ They prepared MIL‐53(Fe)‐coated UCNPs (round shape, diameter of ≈20 nm) modified with lactoferrin for enhanced blood–brain barrier penetration and tumor targeting, significantly inhibiting glioma growth in vitro and in vivo through an enhanced PDT effect combined with CDT and chemotherapy.

Following PDT, PTT employs the heat generated by Fe‐MOF–based composites upon exposure to near‐infrared light to induce localized hyperthermia. This targeted heating approach effectively kills tumor cells while sparing the surrounding healthy tissues. The capacity of Fe‐MOF–based composites to transform light into heat presents a non‐invasive alternative to conventional cancer treatments, offering a precise method to combat tumors that are challenging to reach with traditional surgical techniques. Dong et al. presented a novel strategy for constructing nanomedicines using MIL‐101(Fe) nanoparticles coated with Cu‐TA polymers.^[^ ^150^ ^]^ This design facilitated the encapsulation of the antitumor drug CPT, achieving multiple stimuli‐responsive controlled drug release and enhanced anticancer effects. Notably, the CPT@MIL‐101(Fe)@TA‐Cu nanomedicines exhibited photothermal performance and increased ROS generation, enabling combined tumor therapy through PTT, CDT, and chemotherapy.

#### 4.2.4 SDT

MOFs have been identified as promising candidates for SDT.^[^ ^210^, ^231^, ^232^ ^]^ The presence of porous structures significantly increases the specific surface area, thereby promoting the interaction between ultrasound waves and MOF structures, which leads to enhanced ROS generation.^[^ ^233^ ^]^ Fe‐MOFs can initiate redox reactions by altering their valence states, providing opportunities for the activation of active substances in TME, such as H_2_O_2_ and GSH, and preventing the leakage of Fe ions before reaching the target tumor site.

Liu et al. presented a promising anticancer approach by combining SDT with ferroptosis and ICD through a simple MIL‐53(Fe) based nanosystem.^[^ ^210^ ^]^ They utilized MIL‐53(Fe) to load the chemotherapy drug CM‐272 for bladder cancer treatment, achieving a high loading rate of 56.4%. The Fe^3+^ ions within MIL‐53(Fe) were effective in converting H_2_O_2_ into O_2_ and enhancing ROS production, while simultaneously reducing GSH levels in the TME. This composite also leveraged the sonosensitizing capabilities of MIL‐53(Fe), amplified by 2‐NH_2_‐BDC, and increased oxygen levels, which enabled effective SDT. Furthermore, the elevated oxygen levels attenuated the drug resistance of CM‐272 and facilitated ICD, enhancing the efficacy of subsequent immunotherapeutic approaches.

In another study, Zhao et al. prepared a novel Fe‐MOF–based nanosystem (Figure 14b), GEM‐DVDMS@MOFs@COF‐NRP2 (GDMCN2), targeting GEM‐resistant pancreatic cancer cells with exceptional specificity and efficiency.^[^ ^214^ ^]^ Upon sonodynamic stimulation, GDMCN2 induced autophagy‐dependent ferroptosis by generating substantial ROS, which led to mitochondrial and DNA damage and the activation of ER stress. This innovative approach not only enhanced the delivery and effectiveness of the therapy but also offered a promising strategy to overcome drug resistance, potentially revolutionizing the clinical management of resistant pancreatic cancer.

Combined with the shaping technology of Fe‐MOFs, Zhuang et al. introduced a calcium alginate‐based hydrogel to enhance tumor ferrotherapy by amplifying redox imbalance.^[^ ^215^ ^]^ MIL‐88B(Fe) nanoparticles were surface‐absorbed with PTX and Ce6, then dispersed into an alginate solution. Even the pore sizes of MIL‐88B(Fe) are not large enough to encapsulate the drug molecules, no burst release of PTX was observed. Injected locally, the hydrogel formed at the tumor site, where Ca^2+^induced crosslinking, creating a hybrid hydrogel patch that released therapeutic agents. This system leveraged the enzyme‐like activity of Fe‐MOFs to catalyze H_2_O_2_ into hydroxyl radicals and oxidize GSH, enhancing the oxidative stress within the TME and inhibiting anti‐ferroptosis defenses by the effect of PTX. Additionally, ultrasound radiation stimulated the therapeutic release and triggered Ce6 to produce toxic singlet oxygen, leading to significant tumor treatment efficacy.

#### 4.2.5 Other Synergistic Treatment Strategies

Chiral materials can interact in a stereospecific manner with biological systems, which often comprise chiral molecules. Functionalizing Fe‐MOF–based composites with chiral groups can lead to higher selectivity and binding affinity for certain biological targets, such as cancer cells. Hao et al. prepared a chiral d‐/l‐CuxOS–based nanoplatform encapsulated within Fe‐MOFs, designed to selectively target cancer cells and induce synergistic cuproptosis and ferroptosis with low systemic toxicity.^[^ ^215^ ^]^ This dual‐action nanocomposite released Cu(I) in response to reduced GSH levels, which preferentially targeted mitochondrial enzymes to induce cuproptosis. Concurrently, the breakdown of Fe‐MOF increased Fe levels, augmenting the ferroptosis pathway through a Fenton‐like reaction that produced ROS. This integrated approach effectively suppressed tumor growth, demonstrating the potential of chiral nanomaterials for precise and safe cancer therapy.

Wang et al. positioned Fe‐MOF–based composites as a potent magnetic hyperthermia agent with significant therapeutic potential and exhibited a promising strategy for integrating hyperthermia with ferroptosis‐inducing capabilities for enhanced cancer treatment.^[^ ^128^ ^]^ They synthesized a novel paramagnetic Lac‐FcMOF, functionalized with Lac. The key to the function of Lac‐FcMOF was its utilization of ferrocene units in a dual redox reaction. Once internalized by cells, the ferrocene cation ([Fc(COOH)_2_]^+^) consumed GSH, while the neutral Fc(COOH)_2_ catalyzed the production of hydroxyl radicals by a Fenton‐like reaction. These processes were enhanced under an alternating magnetic field (AMF), causing increased oxidative damage and inhibition of heat shock protein 70 (HSP70) synthesis in the mitochondria due to reduced ATP levels. This resulted in significant downregulation of HSP70 and promoted magnetically modulated hyperthermia therapy (MMHT). Furthermore, the intensified redox dynamics (RDH) induced by Lac‐FcMOF triggered ferroptosis. In vitro and in vivo experiments demonstrated that Lac‐FcMOF effectively targeted HepG2 cells, enhanced RDH, and led to HSP70 downregulation and ferroptosis, synergistically with MMHT, resulting in a tumor inhibition rate of 90.4%.

Multimodal synergistic therapy utilizing Fe‐MOF–based composites, represents a current trend in cancer research, yet it encompasses several limitations. For instance, a recent study introduced a novel nanoplatform, Cu@MIL‐101@PMTPC, which leverages Fe‐MOFs for combined chemodynamic, photodynamic, and immune cancer therapies.^[^ ^138^ ^]^ The innovative design featured in situ growth of CuNPs on MIL‐101(Fe), enhancing the chemodynamic response. Sequential loading of therapeutics (1‐MT, cisplatin, TCPP) and a PDA coating, followed by an oxygen‐generating CaO_2_ layer, addressed tumor hypoxia and facilitated pH‐dependent drug release. This multifunctional platform demonstrated good tumor cell eradication in vitro through synergistic mechanisms of immunogenic cell death and dendritic cell maturation, highlighting its potential for targeted multimodal tumor therapy. Nevertheless, while the study's conclusion on the efficacy of the Cu@MIL‐101@PMTPC nanoplatform is promising, it fails to address critical aspects that could impact its clinical relevance. Specifically, the complexity of the system raises significant challenges in quantifying the loading capacities of each material involved. Discussing therapeutic effectiveness without addressing precise dosages is unconvincing, as the dosage directly influences treatment outcomes and safety profiles. Additionally, the potential interactions between the various therapeutic strategies employed are unclear. It is essential to ascertain whether these modalities act in a synergistic, additive, or antagonistic manner to each other to validate the platform's effectiveness and optimize its design for clinical applications. These gaps must be addressed to substantiate the claims of efficacy and safety.

#### 4.2.6 Bioimaging‐Assisted Therapy

Integrating advanced imaging techniques is crucial for precise cancer diagnosis and therapy monitoring. Fe‐MOF–based composites significantly improve the performance of imaging modalities such as FI, MRI, and persistent luminescent (PL) imaging.^[^ ^56^, ^65^, ^98^, ^234^ ^]^ The magnetic and luminescent properties of Fe‐MOF–based composites improve the clarity and depth of imaging signals, thereby enhancing tumor localization and monitoring therapeutic responses in real time. This not only aids in precise diagnostics but also in tailoring individualized treatment plans. The development of Fe‐MOF–based composites is of significant importance in improving the capabilities of bioimaging for guiding synergistic cancer therapies.^[^ ^223^ ^]^

Jia et al. introduced an approach for glioma therapy that employed NIR‐IIb imaging‐guided self‐enhancing SDT using PCN‐224(Fe)–based nanocomposites.^[^ ^228^ ^]^ These nanocomposites accumulated optimally at the tumor site 10 h post‐injection, enhancing blood–brain barrier penetration and allowing for dynamic treatment monitoring. The Fe‐MOF enhanced ROS generation and reduced GSH levels within the tumor microenvironment, promoting oxidative stress and inducing ferroptosis. Notably, the reduction of Fe^3+^ from the MOF facilitated the restoration of near‐infrared‐IIb imaging, enabling dynamic imaging during the SDT process.

**Figure 15:**
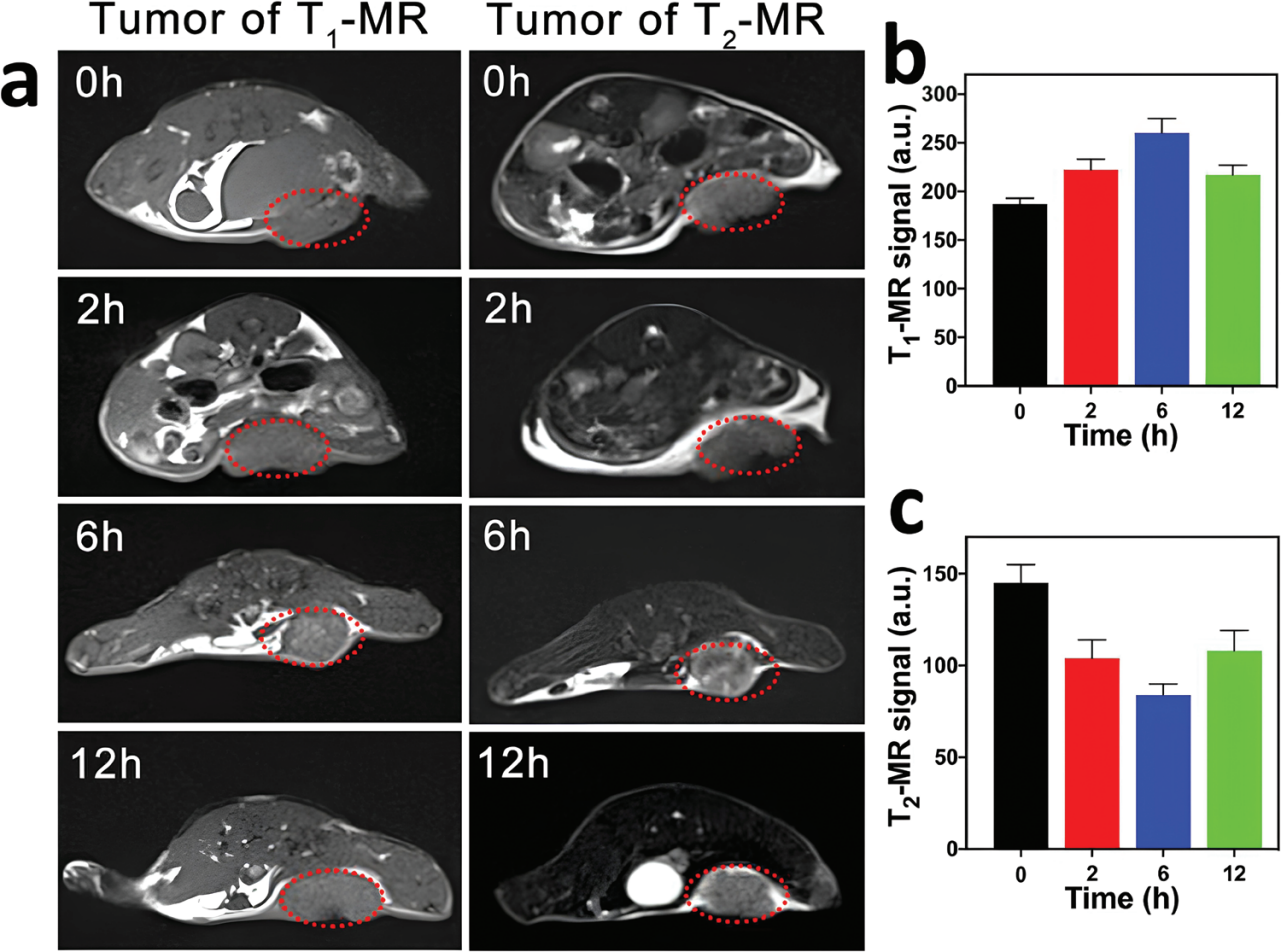
Dynamic MRI imaging results assessing the in vivo biodistribution of Gd/FeMOF‐PEG in tumors. a) T_1_–T_2_ MRI images and b,c) signal intensity values were assessed in tumors at 0, 2, 6, and 12 h post‐injection with Gd/FeMOF‐PEG in BALB/c mice bearing H22 tumors. Reproduced with permission.^[^ ^64^ ^]^ Copyright 2023, ScienceDirect.

To improve the efficacy of Fe‐MOF for MRI, Wang et al. presented a lenvatinib‐loaded Gd/Fe composites that served as an effective dual MRI contrast agent, enabling therapeutic diagnostics within the TME and integrating microwave‐enhanced dynamic therapy with immunotherapy.^[^ ^64^ ^]^ The bimetallic nature of the MOFs capitalized on Gd's utility in MRI contrast enhancement and Fe's role in augmenting ROS generation and ICD. The maximum tumor T1 and T2 MRI signal intensity was evident 6 h post‐injection, a time point identified as optimal for implementing microwave irradiation (**Figure** **15**). This multifunctional approach facilitated MRI‐guided targeting of tumor tissues and remodeled the TME by releasing lenvatinib to inhibit PD‐L1 expression. In vivo results demonstrated the nanoplatform's potential to strengthen antitumor immunity and diminish tumor recurrence, indicating a promising strategy for cancer theranostics. However, the introduction of Gd into MOFs, which degrade much faster than the commonly used Gd chelates, is highly risky and questionable. As Gd ions are released into the body when the MOF is degraded by phosphates. In the case of Gd chelates, they are mostly excreted by urines due to their ultra‐small size.

**Figure 16:**
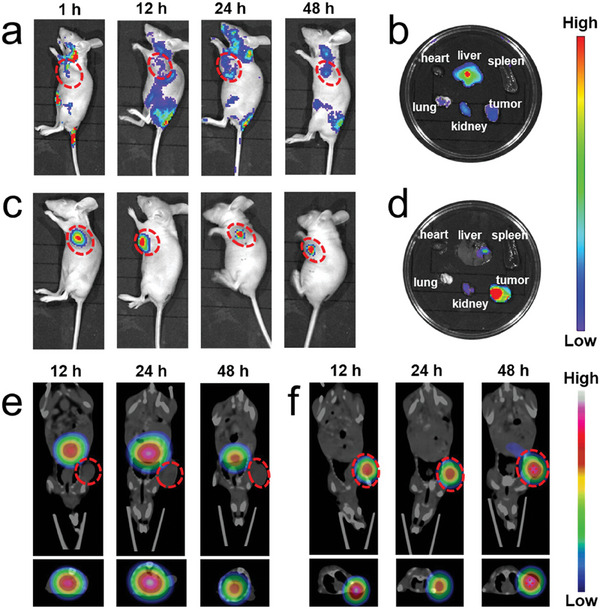
a,c) In vivo FI of tumor‐bearing mice at 1, 12, 24, and 48 h after intravenous (a) and intratumoral (c) injection of MIL‐101(Fe)/PEG‐FA/Cy. b,d) Ex vivo FI of major organs of one subject at 48 h after intravenous (b) and intratumoral (d) injection of MIL‐101(Fe)/PEG‐FA/Cy. e,f) SPECT/CT imaging of U87MG‐bearing mice at 12, 24, and 48 h after being intravenously (e) and intratumorally (f) injected with ^177^Lu‐MIL‐101(Fe)/PEG‐FA (tumors are circled in red). Reproduced with permission.^[^ ^147^ ^]^ Copyright 2023, ACS.

Liang et al. introduced a ^177^Lu‐labeled MIL‐101(Fe)/PEG‐FA/cyanine7(Cy) as a multifunctional nanoplatform for integrating FI, SPECT, and endoradiotherapy.^[^ ^147^ ^]^ The platform demonstrated tumor targeting and anticancer efficacy in glioma models, showcasing the potential of Fe‐MOFs in precise pre‐evaluation and treatment of cancer. As shown in **Figure** **16**a, the fluorescent probe rapidly accumulated in the liver, lungs, spleen, kidneys, and tumors within an hour post‐intravenous injection. Over the subsequent 48 h, the tumor site's fluorescence intensity gradually increased, while that in the liver, lungs, and spleen decreased. This indicated that the nanocarrier possessed excellent tumor affinity and was capable of timely clearance from normal organs and tissues. The substantial accumulation of fluorescence in tumor tissue observed in Figure 16b further proved the tumor‐targeting capability of MIL‐101(Fe)/PEG‐FA/Cy. Notably, the intravenous injection of MIL‐101(Fe)/PEG‐FA/Cy exhibited high uptake rates in the reticuloendothelial system organs such as the liver and lungs, and also demonstrated prolonged retention in the kidneys. Furthermore, the tumor retention of MIL‐101(Fe)/PEG‐FA/Cy7 following intratumoral injection was examined. It was found in Figure 16c,d that the fluorescence signal was well retained at the tumor site, with no significant accumulation observed in other normal organs or tissues. However, the results of SPECT/CT imaging conducted 12 to 48 h after intravenous injection of Fe‐MOF–based composites demonstrated no significant tumor uptake, with all tracers remaining in the reticuloendothelial system (Figure 16e). The apparent discrepancy in tumor affinity observed between FI and SPECT imaging in the body was attributed to the differences in tissue penetration capabilities and detection sensitivity between the two imaging techniques.^[^ ^147^ ^]^ The results from SPECT/CT imaging were consistent with those from FI studies (Figure 16f). This biological imaging investigation indicated that optical imaging is a promising option due to its sufficient spatial resolution to visually indicate cellular uptake. However, the validity of the biological distribution provided by FI probes should be carefully verified. Another concern is that fluorescent probes, especially those constructed by loading dye molecules into porous carriers, should possess sufficient in vivo stability to complex physiological conditions.

### 4.3 Antimicrobial

The World Health Organization has identified antibiotic resistance as a significant public health challenge, marking our entry into the post‐antibiotic era. To address this challenge, Fe‐MOFs–based composites may offer new solutions, primarily involving: i) DDS; ii) CDT; iii) Phototherapy; iv) Bioimaging‐assisted therapy. **Table** **7** provides a summary of recent advances in Fe‐MOF–based composites in the field of antimicrobial treatment, highlighting the strategies and in vitro/in vivo models employed in their study.

**Table 7:** Recent advances in Fe‐MOFs–based composites in antimicrobial treatment.

Fe‐MOFs	Strategy	Bioactive agent	Particle size [nm]	Zeta potential [mV]	In vitro/In vivo model	Ref.
MIL‐100(Fe)	DDS	Fe_3_O_4_@SiO_2_, ciprofloxacin (LC = 16.3wt%, EE = 97.5%)	<200	18	*P. aeruginosa, S. epidermidis*, and MCF‐7 cells	[235]
MIL‐88B(Fe/Co) derivative	DDS	spiro‐oxindole derivatives	1000–2000		*E. coli*, *S. aureus*, and MRSA	[237]
[Fe(atrz)(IO_3_)_2_]*_n_*	DDS					[238]
MIL‐101(Fe)	DDS	silver nanoparticles (AgNPs)	2200	−7.8	*S. aureus*, *S. saprophyticus*, and *E. coli*	[239]
MIL‐88(Fe)	DDS	TC (EE = 90.1%)	>1000	18.3	*E. coli* and *S. aureus*	[240]
MIL‐100(Fe)	DDS	azidothymidine (AZT)‐triphosphate (TP) (LC = 42 wt%)	238		peripheral blood mononuclear cells	[60]
MIL‐100(Fe)	DDS	AZT‐TP (LC = 25 wt%], AZT‐monophosphate (MP) [LC = 40 wt%)	200	−40		[241]
MIL‐101(Fe)	DDS	T‐705 (LC = 27 wt%)	200–500	15.8	*S. aureus*, influenza A (H1N1) virus	[242]
MIL‐101(Fe) derivative	CDT		230		H22 liver cancer xenograft tumor‐bearing mice, mice infected with MRSA	[236]
MIL‐88B(Fe)‐NH_2_	CDT	GOx	<200		mice infected with MRSA	[168]
Fe/Cu‐MOF	CDT	GOx	300	−13	rat infected with *S. aureus*	[173]
Fe/Zn‐BDC‐NH_2_	CDT		300–800	23	mice infected with *E. coli*	[243]
MIL‐101(Fe)	CDT				*E. coli* and *S. aureus*	[169]
MIL‐88B(Fe)‐NH_2_@Fe_3_O_4_	CDT	GOx (LC = 8 wt%)	800	−22.7	mice infected with MDR‐AB	[101]
Al/Fe‐TCPP	CDT	GOx (LC = 4 wt%)	342	−8.2	*E. coli* and *S. aureus*	[244]
MIL‐53(Fe)	CDT				coronaviruses (HCoV‐229E and SARS‐CoV‐2)	[245]
MIL‐53(Fe)	CDT	GOx (LC = 6 wt%)	220	−16.7	*S. aureus*‐infected mice	[139]
Fe‐BDC‐X	CDT, SDT	rose bengal (RB) (LC = 20 wt%), AgNPs	170–230	−17.7	drug‐resistant *C. albicans*	[246]
Mo/Fe‐MOF	radical generation	peroxymonosulfate			sulfate‐reducing bacteria, *P. aeruginosa* and *E. coli*	[247]
MIL‐100(Fe)	PDT	MB (LC = 2 wt%, EE = 84%)	57	−29.7	*C. trachomatis*	[248]
Prussian blue (PB) MOF	CDT, PDT, PTT	MoS_2_	650	−25.2	mice infected with *S. aureus*	[249]
Ti_3_C_2_ MXene/Fe‐BDC	CDT, PTT		200	5	mice infected with MDR‐AB	[250]
MIL‐53(Fe)	photocatalyst	riboflavin, NaYF_4_:Yb,Tm	100		*E. coli* and *S. aureus*	[251]
PCN‐224(Fe)	photocatalyst	zero‐valent Fe	200		*E.coli*, *P. aeruginosa*, *S. aureus*, and *V. parahaemolyticus*	[252]
MIL‐88B(Fe)‐NH_2_	DDS, MRI	AgNPs	>400		*E. coli*, *S. epidermidis*, and *S. aureus*	[172]
Ag@CuPB	CDT, PTT, surface‐enhanced Raman scattering (SERS) imaging		104	−14.8	*S. aureus*‐infected mice	[253]

LC: loading capacity; EE: Encapsulation efficiency.

Recent work has demonstrated that Fe‐MOF materials can simultaneously achieve antitumor and antimicrobial effects, confirming their similar actions between antitumor and antimicrobial treatment.^[^ ^235^, ^236^ ^]^ For example, the work of Sun et al. demonstrated the utility of Fe‐MOFs in concurrently addressing cancer and bacterial infections.^[^ ^236^ ^]^ The study introduced FeS_2_@MoS_2_, a bimetallic sulfide nanomaterial derived from MIL‐101(Fe), which demonstrated enzyme‐mimetic activities against tumors and bacteria. While the basic principles of antitumor and antimicrobial actions are similar, there are some differences in their practical applications. i) Anticancer treatment typically involves inducing apoptosis, necrosis, or autophagy in tumor cells. In contrast, antimicrobial treatment especially for antibacterial treatment focuses on disrupting the cell walls or membranes of microbes, thereby blocking their critical life‐sustaining pathways; ii) DDS for anticancer treatment often requires consideration of tumor penetration and the EPR effect. Antimicrobial treatments, especially those targeting pathogens that form biofilms, necessitate the development of strategies to penetrate biofilms and continuously release antimicrobial agents in the infected area; iii) The characteristics of the TME (defined by low pH and high reducing property) and microbial growth environments have a significant impact on the degradation of Fe‐MOF–based composites, drug release, and the efficiency of ROS generation; iv) In contrast to mammalian cells, different bacteria employ distinct redox strategies, resulting in notable variations in the therapeutic effects of ROS‐based Fe‐MOF treatments. These differences highlight the need for tailored strategies to maximize therapeutic efficacy in different clinical scenarios.

#### 4.3.1 DDS

DDS enhance the bioavailability of antimicrobial agents by modifying or encapsulating them in Fe‐MOFs, which are characterized by their high surface area and tunable structures. This approach helps reduce the toxicity and resistance associated with high doses of antimicrobials.

Compounds containing pyrazole, spiro, indigo, uracil, and indole moieties exhibit antibacterial, antifungal, anti‐inflammatory, antiviral, and antitumor activities. These heterocyclic structures are now popular in the drug market, demonstrating efficacy in inhibiting various pathogens and offering potential as broad‐spectrum therapeutic agents.^[^ ^237^ ^]^ Tavakoli et al. functionalized the MIL‐88 (Fe, Co) with heterocyclic moieties possessing antibacterial properties using click chemistry and demonstrated potent antibacterial activities against *S. aureus*, MRSA and *E. coli*.^[^ ^237^ ^]^ Furthermore, some cyanin dyes featuring heterocyclic structures have been incorporated into Fe‐MOFs for biomedical applications such as bioimaging and phototherapy.^[^ ^254^, ^255^ ^]^ However, the synergistic effect between their inherent antibacterial activity and the effect of Fe‐MOFs has seldom been investigated.

In contrast to antibacterial therapies, which target bacterial cell walls, proteins, or metabolic pathways to inhibit bacterial growth or kill bacteria, antiviral treatments mostly focus on inhibiting viral replication and entry into host cells. Xu et al. used MIL‐101(Fe) to in situ encapsulate the antiviral drug favipiravir (T‐705). MIL‐101(Fe)@T705 demonstrated good antiviral activity against the H1N1 virus and antibacterial properties against *S. aureus*. The results indicated that MIL‐101(Fe)‐T705 exhibited superior antiviral activity and lower cytotoxicity in comparison to T‐705 alone. The suggested mechanism involved MIL‐101(Fe) preventing the virus from attaching to the cell membrane, followed by the release of T‐705 to inhibit viral replication.^[^ ^242^ ^]^ However, the use of toxic solvents (DMF) and high temperatures (150 °C) for in situ drug encapsulation raised concerns about potential toxicity and drug inactivation. Furthermore, the potential antiviral effects of ROS generated by Fe^2+^/Fe^3+^ was mentioned in another study on the use of MIL‐53(Fe) against coronaviruses.^[^ ^245^ ^]^

**Figure 17:**
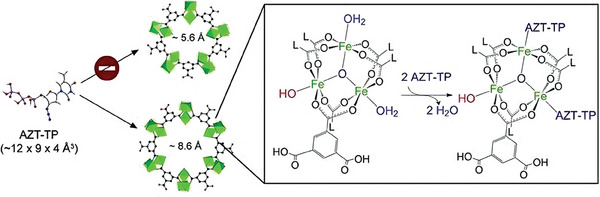
The interaction between AZT‐TP and MIL‐100(Fe) nanoMOFs. AZT‐TP molecules can cross the hexagonal microporous windows, to interact with open metal sites located within large mesoporous cages. In contrast, the pentagonal openings are too narrow to allow drug penetration within the small mesoporous cages. Once inside the nanoMOFs porous core, AZT‐TP can efficiently interact with the matrix by coordination between phosphate and open metal sites, replacing the previously coordinated water molecules.^[^ ^60^ ^]^ Copyright 2013, Wiley.

Antiretroviral treatments, which are specifically designed to target retroviruses like HIV, inhibit reverse transcription and integrate proviral DNA into the host genome. These therapies often involve nucleoside reverse transcriptase inhibitors and require efficient intracellular conversion to active forms, which presents a significant challenge in achieving clinical efficacy. This distinction underscores the necessity for the development of advanced delivery systems to enhance the stability, cellular uptake, and targeted release of antiviral and antiretroviral agents. AZT are prodrugs that inhibit retrotranscription and proviral DNA synthesis, protecting cells from HIV. These must be metabolized by intracellular kinases into active TP derivatives to exert their antiretroviral activity. However, the inefficient triphosphorylation of these drugs limits their clinical efficacy, leading to the development of drug resistance and the administration of high therapeutic doses, which can result in adverse effects.^[^ ^60^ ^]^ Nucleoside analogues also face challenges due to their poor stability and low cellular uptake when administered directly.^[^ ^241^ ^]^ Gref et al. first reported the unique mechanism that the open metal sites of MIL‐100(Fe) formed strong interactions with drug phosphates, enabling high drug loading efficiencies and controlled release (Figure 17).^[^ ^60^ ^]^ Their study demonstrated the use of MIL‐100(Fe) for delivering AZT‐TP. The nanoMOFs exhibited high loading efficiency and an unexpected slow release in simulated body fluids for a few days, which was attributed to the strong interactions between the phosphate groups of AZT‐TP and the unsaturated iron(III) sites. This highlights the unique ability of such Fe‐MOFs to bind hydrophilic drugs bearing strongly complexing groups (phosphates, carboxylates, cathecolates, etc.) leading to a new drug release mechanism with an exchange between the phosphates from the body fluids and the coordinated drug molecules. These AZT‐TP‐loaded MIL‐100(Fe) were able to successfully penetrate HIV target cells, deliver the drug intracellularly, and significantly protect against HIV infection.^[^ ^60^ ^]^ In another related study, Gref et al. compared the release profiles of AZT‐MP and AZT‐TP loaded in MIL‐100(Fe).^[^ ^241^ ^]^ They found that AZT‐MP molecules were released faster in physiological buffer compared to AZT‐TP. This suggested that the release rate of phosphate drugs from MIL‐100(Fe) could be controlled by varying the number of phosphate groups, thereby meeting different therapeutic needs.

#### 4.3.2 CDT

The presence of open metal sites in selected Fe oxoclusters based Fe‐MOFs–based composites and the delivery of Fe^2+^ from MOFs’ degradation can serve as catalysts for Fenton‐like reactions, which facilitate the transformation of reactive substances, such as H_2_O_2_ and peroxysulfates, into highly reactive radicals.^[^ ^256^ ^]^ These latter can destroy microorganisms, including bacteria, fungi, and viruses, and play a role in ferroptosis‐like death. Zhong et al. introduced zinc‐doped Fe‐BDC‐NH_2_ (ZFMs) for antibacterial treatment and wound care.^[^ ^243^ ^]^ The ZFMs demonstrated a superior capability to produce ROS that effectively inactivated drug‐resistant *E. coli* with over 98% lethality at a relatively low concentration (150 µg mL^−1^). Additionally, the doping of Zn promoted wound healing by up‐regulating VEGFα and supporting fibroblast proliferation. Moreover, the enhanced stability of ZFMs prevented the explosive release of metal ions seen in traditional Zn‐MOFs or Fe‐MOFs, thereby avoiding toxicity. In another study, Zhang et al. introduced a Fe‐BDC–based MOF integrated with RB and AgNPs, named MRA, for the treatment of drug‐resistant *C. albicans*.^[^ ^246^ ^]^ The MRA combined CDT and SDT, exhibiting potent fungicidal capabilities in acidic conditions that mimic the vaginal environment. Demonstrating a remarkable 99.999% efficacy against drug‐resistant *C. albicans* at a concentration of 120 µg mL^−1^.

**Figure 18:**
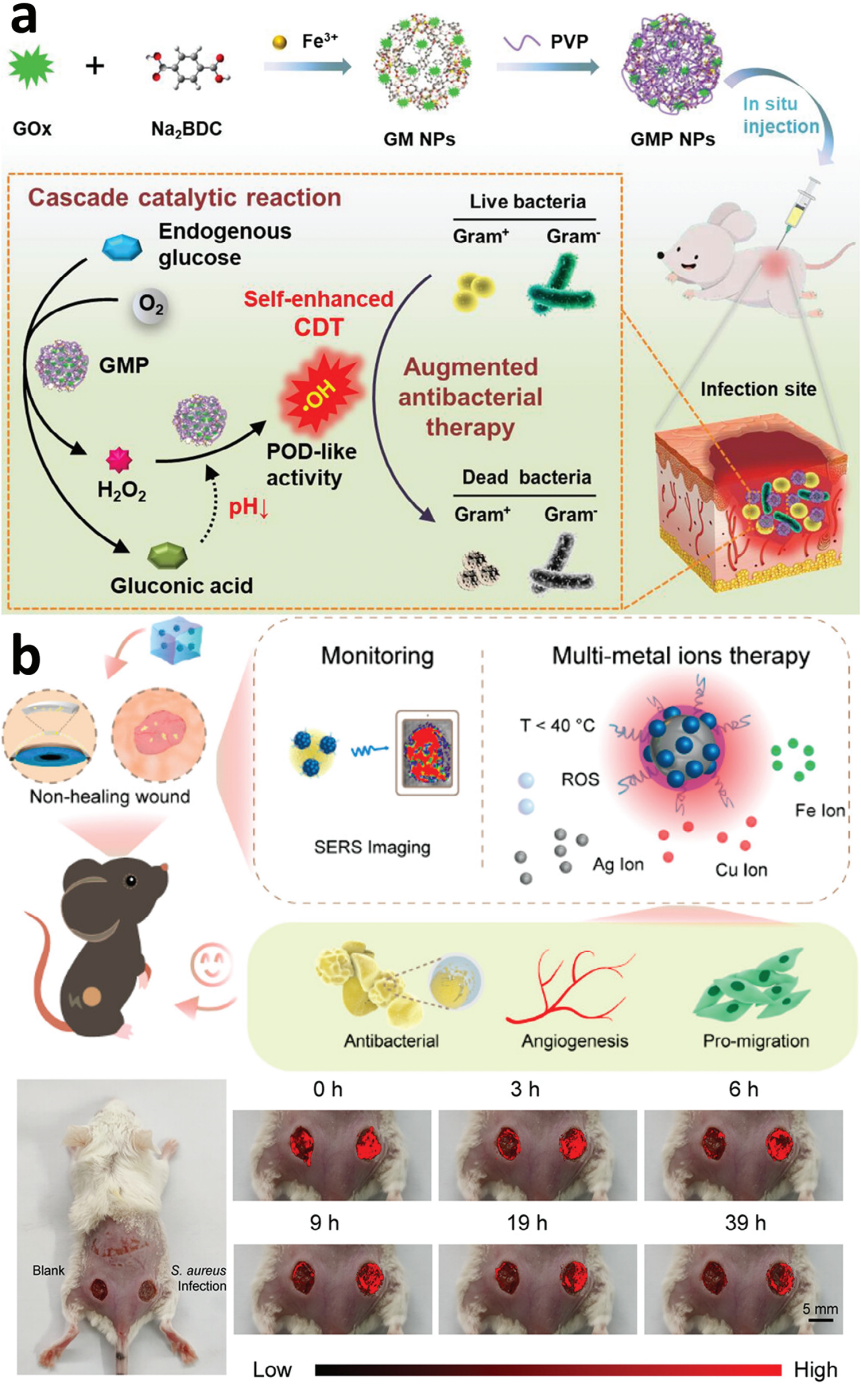
a) Schematic diagram of the synthesis of GOx@MIL‐53(Fe)@PVP with enhanced cascade catalytic activity and its application in wound disinfection. Reproduced with permission.^[^ ^139^ ^]^ Copyright 2023, ACS. b) (up) Schematic diagram of ACPA‐based zero‐background SERS bioimaging‐guided multimetal ions synergistic therapy for an infected wound. (down) Photographic image of a BALB/c mouse with blank and *S. aureus*‐infected wounds post‐ACPA application, including SERS images at 2086 cm^−1^ of *S. aureus* (right) and blank (left) infected wounds at various time points. Reproduced with permission.^[^ ^253^ ^]^ Copyright 2023, ACS.

The addition of exogenous H_2_O_2_ is a feasible method for enhancing the effect of CDT. However, it inevitably results in toxicity on normal tissues. GOx, one of the most widely used food enzymes, can catalyze the oxidation of glucose to H_2_O_2_ and gluconic acid. In recent studies, GOx has often been paired with Fe‐MOFs to act as an endogenous H_2_O_2_ producer, thereby enhancing the CDT effect. Ou et al. introduced a novel GOx@MIL‐53(Fe)@PVP cascade catalytic nanosystem, which employed MIL‐53(Fe) to embed GOx through in situ method (**Figure** **18**a).^[^ ^139^ ^]^ This system attempted to convert glucose into gluconic acid and H_2_O_2_, with the H_2_O_2_ further reacting to produce hydroxyl radicals via the peroxidase‐like activity of MIL‐53(Fe), thus enhancing CDT. Their results showed that the nanosystem demonstrated outstanding antibacterial efficacy, achieving kill rates of 99.7% and 99.8% against *S. aureus* and *E. coli*, respectively, and promoting rapid wound healing in bacterial‐infected mouse models with minimal damage to healthy tissues. However, concerns remain regarding the synthesis conditions of MIL‐53(Fe), which involve high‐temperature hydrothermal reactions and toxic reagents that could potentially inactivate GOx. Moreover, post‐synthesis characterization revealed that GOx is here only loaded at 5.9 wt% on the external surfaces of MOFs. Given these considerations, unless using a much larger pore size MOF and an in situ route to embed the enzyme into the pores (e.g., MIL‐100(Fe)^[^ ^257^ ^]^), employing a post‐synthesis loading approach for GOx might better preserve enzyme activity and avoid decreasing loading efficiency when removing the toxic organic agent, highlighting the importance of selecting an appropriate encapsulation strategy to maximize the therapeutic potential of Fe‐MOF–based DDS. In another study using GOx, Song et al. enhanced the peroxidase‐like activity by using MIL‐88B(Fe)‐NH_2_ coupled to Fe_3_O_4_ nanoparticles and immobilize GOx through amide coupling.^[^ ^101^ ^]^ The concurrent use of Fe‐MOFs and Fe_3_O_4_ nanoparticles amplified a self‐activated cascade reaction, displaying a higher affinity for H_2_O_2_. Furthermore, this approach leveraged glucose as a non‐toxic fuel, thereby reducing the adverse effects typically associated with direct H_2_O_2_ application and significantly improving biosafety. Then the generated hydroxyl radicals caused substantial damage to bacterial cell structures, effectively combating drug‐resistant bacterial infections while minimizing harm to healthy tissues. However, to our knowledge, no study has yet revealed how the structure of Fe‐MOF protects the activity of GOx, what types of Fe‐MOF are suitable for encapsulating GOx, or whether GOx exposed on the surface of MOFs might risk deactivation. Understanding these mechanisms would be beneficial for the development of a new generation of Fe/GOx systems.

In addition, Fenton‐like reactions can treat wastewater containing harmful microbes. Wang et al. highlighted the role of Fe ions in MOFs for developing novel catalysts for environmental antimicrobial applications.^[^ ^247^ ^]^ The flexible conversion ability of Fe^2+^/Fe^3+^ ions significantly contributes to the efficient activation of peroxymonosulfate, leading to the generation of powerful oxidizing radicals. These radicals effectively kill waterborne pathogens, offering a promising strategy for environmental sanitation.

#### 4.3.3 PDT and PTT

Exposure to specific wavelengths of light allows Fe‐MOF–based composites to generate ROS through PDT, or locally increase the temperature of the infected area through PTT. Phototherapy affects only the microbes in the irradiated area, thus preventing oxidative stress or thermal damage to healthy tissues. The mechanisms of action differ from antibiotics, reducing the possibility of microbial resistance. However, phototherapy presents some shortcomings: i) Short wavelengths struggle to penetrate deeply into biological tissues, necessitating more effective treatments for deeper regions; ii) The studies employed high‐powered light sources, raising safety concerns; iii) High treatment temperatures (above 45 °C) and excess ROS can cause damage to normal tissues and potentially trigger an inflammatory storm in the body.^[^ ^258^ ^]^

Yang et al. introduced a novel MoS_2_@PBMOF composite, leveraging a core–shell interface of PB MOF and MoS_2_ nanosheets to enhance photocatalytic and photothermal activities under visible light irradiation.^[^ ^249^ ^]^ This heterostructure significantly increased the separation efficiency of photo‐induced electron–hole pairs and enhanced oxygen species adsorption, facilitating the production of ROS and the efficient release of Fe ions. Moreover, the composite demonstrated bactericidal efficiencies of 99.73% and 99.58% against *S. aureus* and *E. coli*, respectively, offering a synergistic approach that combines PDT, PTT, and CDT to effectively treat bacterial infections and promote wound healing. However, their use of PTT generated temperatures up to 55 °C, likely causing irreversible damage to normal tissues. Zhao et al. synthesized a novel composite of Ti_3_C_2_‐MXene and Fe‐BDC, named MXM, that utilized NIR‐induced localized surface plasmon resonance to eradicate bacteria.^[^ ^250^ ^]^ This activation generated hot electrons that enhanced the Fenton reaction on the surface of Fe‐MOFs, thereby boosting the CDT effect. In combination with phototherapy, the composite exhibited potent antimicrobial properties and facilitated angiogenesis and collagen deposition, contributing to accelerated wound healing, as evidenced in both in vitro and in vivo studies. However, to achieve mild PTT, a strong light source (808 nm, 0.84 W cm^−2^) and the addition of extra H_2_O_2_ are required in their work, which may raise concerns regarding the safety of the treatment. Recently, our group utilized MIL‐100(Fe) to load size‐matched cyanine dye, IR775. The MIL‐100(Fe)@IR775 nanoparticles exhibited exceptional photostability and minimal dye release in a physiological environment, achieving high eradication efficiency against MRSA under low power of LED irradiation (808 nm, 0.096 W cm^−2^). The nanoparticles were incorporated into electrospun gelatin films to develop wound dressings, which effectively promoted the healing of MRSA‐infected mice wounds under mild LED irradiation (808 nm, 0.096 W cm^−2^).^[^ ^259^ ^]^

Similar to PDT, the photocatalytic properties of Fe‐MOFs can also be utilized to eliminate harmful microbes in wastewater, where the material's toxicity requirements are relatively lower compared to in‐body biomedical research. Shu et al. synthesized NaYF4:Yb,Tm@MIL‐53(Fe)–based nanocomposites using an efficient hydrothermal method, demonstrating significant photocatalytic and antibacterial activities under high‐power full‐spectrum sunlight and 980 nm NIR light irradiation, effectively treating organic pollutants and pathogens in wastewater.^[^ ^251^ ^]^ The nanocomposites exhibited potent antibacterial effects, with concentrations of 200 and 12.5 µg mL^−1^ completely inhibiting the growth of *E. coli* and *S. aureus*, respectively. However, the robust antibacterial performance is dependent on the high intensity of the light source. In another study, Zhao et al. prepared a nano zero‐valent Fe‐doped PCN‐224 for the synergistic remediation of uranium‐contaminated wastewater, demonstrating high efficiency in inhibiting different microbes.^[^ ^252^ ^]^ This system leveraged the photocatalytic activity to generate ROS, promoting antimicrobial and anti‐algae effects. This suggests that it has the potential for applications in both the environmental and biomedical domains. However, further studies are necessary to evaluate the potential toxicity of this composite material for biomedical applications.

#### 4.3.4 Bioimaging‐Assisted Therapy

Bioimaging‐assisted therapy is particularly suited for deep tissue infections or hard‐to‐reach infection sites. Fe‐MOF–based composites can serve as contrast agents for MRI, enhancing imaging signals to provide clear images of lesion areas. Moreover, by combining Fe‐MOFs with fluorescent markers, such as fluorescent dyes or quantum dots, real‐time visualization of diseased areas through fluorescence imaging is achievable, aiding in the monitoring of infection progression and treatment outcomes. Notably, Guo et al. presented the development of a novel theranostic nanoprobe, silver@cupriferous PB aptamer (ACPA), designed for the multifaceted treatment of chronic diabetic wounds. ACPA leveraged its NIR‐triggered mild PTT to promote the release of multimetal ions (Ag, Cu, and Fe).^[^ ^253^ ^]^ This combination of PTT and CDT achieved effective antibacterial activity, epithelium migration, and vascularization, addressing major challenges in the management of non‐healing diabetic wounds. Additionally, ACPA incorporated a core–satellite nanostructure that enhanced its SERS capabilities, enabling precise, zero‐background SERS imaging to monitor residual bacteria during the healing process, as shown in Figure 18b. Despite its high photothermal conversion efficiency of 38.5%, the practical application may be limited as it requires a high‐power light source (2 W cm^−2^) for optimal thermal treatment as well as rely on a somehow complex potentially quite toxic formulation.

### 4.4 Gas Therapy

**Figure 19:**
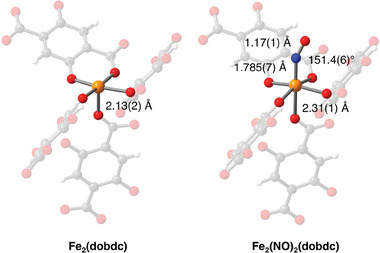
Structures of Fe_2_(dobdc) and Fe_2_(NO)_2_(dobdc) as determined from Rietveld analysis of neutron powder diffraction data. Orange, blue, red, gray, and white spheres represent Fe, N, O, C, and H atoms, respectively. Reproduced with permission.^[^ ^262^ ^]^ Copyright 2015, ACS.

MOFs have attracted significant attention for their potential use in gas storage. Gases such as NO, hydrogen sulfide (H_2_S), and carbon monoxide (CO) play a crucial role in regulating numerous physiological and pathological processes, demonstrating wound healing, anticancer, and antibacterial properties. The efficacy of carriers for gas delivery is dependent on their capacity to release the gas at a controlled rate. Burst, high‐concentration releases may potentially cause adverse effects of gas therapy. The open metal sites and/or chemical functionalities within the pores of Fe‐MOFs are particularly useful for coordinating and adsorbing gases. In 2013, Morris et al. first investigated the adsorption and release under wet gas of biologically active NO using Fe‐MOFs.^[^ ^260^ ^]^ The study on MIL‐88(Fe) demonstrated NO loading capacities ranging from 1 to 2.5 mmol g^−1^, and MIL‐88A(Fe) releasing a total of 0.3 mmol g⁻¹ NO over 16 h in PBS. Fe‐MOFs with open metal sites can undergo reduction, and the presence of Fe^2^⁺ open metal sites facilitate a unique back donation interaction with NO molecules. This interaction enables a prolonged NO release under humid conditions, extending to over 20 h at the biological level.^[^ ^261^ ^]^ Later, Long et al. developed microporous Fe_2_(dobdc), capable of slowly releasing up to ≈4 mmol g^−1^ of NO under wet gas over 10 days.^[^ ^262^ ^]^ The strong binding of NO to Fe^2^⁺ open metal sites is shown in **Figure** **19**. However, the synthesis of this MOFs necessitated the use of DMF and methanol as solvents, which brought potential toxicity concerns.^[^ ^263^ ^]^ While all these MOFs release NO in liquid phase in a few minutes only preventing from their use in body fluids, recently, some of us employed the hydrothermal method to synthesize an ultra‐narrow pores Fe bisphosphonate MOF, denoted MIP‐210(Fe), which exhibited an adsorption capacity of ≈1.9 mmol g^−1^.^[^ ^264^ ^]^ This MOFs permitted the gradual replacement of NO by H_2_O in biological media, enabling an extended NO delivery period of at least 72 h, and exhibited ability in promoting angiogenesis.

In another study, partially reduced MIL‐100(Fe) was developed as a CO carrier, exhibiting a CO release time of up to 24 h under wet nitrogen. The prolonged release of CO played a crucial role in effectively mitigating inflammation by modulating macrophage polarization and cytokine expression, while also promoting endothelial cell growth.^[^ ^265^ ^]^

In addition to direct gas delivery, it is also possible to load the gas donor onto the Fe‐MOFs to catalyze in situ generation of gas in a series of biological reactions leading to indirect gas delivery. For instance, Hong et al. immobilized a NO donor [Fe_2_(µ‐SCH_2_CH_2_COOH)_2_(NO)_4_] onto MIL‐88B, thereby enhancing the oral bioavailability of NO.^[^ ^266^ ^]^ Similarly, Chung et al. conjugated a NO donor ([Fe(µ‐S‐thioglycerol)(NO)_2_]_2_) with MOF‐derived porous Fe_3_O_4_@C, followed by encapsulating them in thermo‐responsive PLGA microspheres.^[^ ^267^ ^]^ This system enabled controlled NO release through an AMF, resulting in effective antibacterial activity and improved wound healing. Recently, Ji et al. employed PCN‐223(Fe) loaded with the anticancer drug irinotecan and the NO donor l‐arginine.^[^ ^268^ ^]^ Their approach effectively reversed multidrug resistance in cancer cells and enhanced anticancer efficacy through the combined effects of chemotherapy and gas therapy. In another anticancer study, Yao et al. introduced MIL‐100(Fe)@DOX functionalized with the CO donor [Mn(CO)_5_Br]. These nanocomposites achieved NIR‐responsive CO‐DOX combination cancer therapy, offering a novel strategy for synergistic cancer treatment.^[^ ^269^ ^]^

## 5 Conclusions and Perspective

Over the past decade, many researches have studied porous biocompatible and biodegradable Fe‐MOFs as potential platforms for biosensing, anticancer, and antimicrobial diagnostics and therapies. Additionally, Fe‐MOF–based nanozyme systems have demonstrated significant potential in modulating inflammatory responses, like in the treatment of arthritis^[^ ^270^ ^]^ and Parkinson's disease.^[^ ^271^ ^]^ By regulating oxidative stress and reducing pro‐inflammatory cytokines, Fe‐MOF nanozymes can mitigate the progression of chronic inflammatory diseases. Despite a series of proofs of concepts at the laboratory scale, the translation of Fe‐MOFs into clinical products remains an unrealized goal. So far these materials have shown most substantial breakthroughs in industrial applications gas storage and separation, water harvesting, and catalysis. One can leverage the industrial breakthroughs of MOFs, including Fe‐MOFs, to significantly advance the field of biomedicine, particularly by focusing on the pathomechanisms and therapeutic strategies of specific diseases. For example, the catalytic properties of MOFs have been extensively utilized in the proof‐of‐concept stages of bio‐applications. The gas storage capabilities of MOFs also have opened new avenues for wound care and the treatment of chronic inflammatory diseases through gas therapy. Innovative research into new MOFs can enhance gas therapy. For instance, the recent development of ultra‐microporous MIP‐210(Fe), reported by our group, is promising to advance long‐term gas therapy.^[^ ^264^ ^]^ It is important to note that biomedical applications impose far stricter requirements on MOFs than industrial uses. Before new MOFs can be integrated into clinical practice, it is essential to conduct systematic advanced studies about their toxicity and biodistribution in relation to their composition, particle size, stability, morphology, surface chemistry, and administration routes. The integration of comprehensive in vitro and in vivo evaluations, in conjunction with advanced modelling techniques, will facilitate a more comprehensive understanding of the interactions between these materials and biological systems.

While promising, the translation of new therapeutic platforms is a notoriously slow and costly process. Currently, FDA approved DDS like liposomes and hydrogels have increased research interest in developing Fe‐MOF–based composites based on surface engineering and shaping, but also prolong the clinical approval process for Fe‐MOFs products. The realization of environmentally friendly, low‐cost, and customizable production represents a significant advantage of Fe‐MOFs over current approved DDS. Although individual Fe‐MOFs have successfully been scaled from laboratory to industrial production in a green and efficient manner, they have not yet met some of the customized medical needs. Moreover, researchers must continually reassess potential administration routes. If most studies have theoretically improved the targeting capability of Fe‐MOFs through strategies like surface engineering, yet they still cannot avoid off‐target effects and systemic toxicity, which often leads to adverse systemic effects, making intratumoral injections seem a safer option. However, such injections are not suitable for diffuse or multiple tumors and are less feasible for deep, hard‐to‐locate, or difficult‐to‐access tumors. Additionally, a more disease‐driven perspective with appropriate strategies is necessary to face current medical challenges. Whether used alone, or in combination therapy, the dosage of materials shall be carefully considered to optimize therapeutic outcomes and minimize adverse effects. This is particularly challenging when following combination therapies, where the final nanocarrier system often involves multiple drugs. Determining the optimal loading capacity, encapsulation efficiency, and release profile for each drug component adds further complexity, making the precise determination of therapeutic dosages more difficult.

Despite the promising potential of Fe‐MOF–based combination therapies, there remain several critical research challenges to be addressed. Foremost among these is the insufficient investigation into the interaction mechanisms between different therapeutic modalities, particularly the way the multifunctionality of Fe‐MOFs can be harnessed to achieve synergistic effects in combination therapies. Addressing these issues is essential for the advancement and development of more effective multifunctional therapeutic platforms. Moreover, the translation of Fe‐MOF–based combination therapies from laboratory research to clinical practice still face significant challenges. In addition to the occasional complexity to the production process, regulatory agencies shall establish comprehensive evaluation criteria for these complex systems. Also, the lack of robustness of the larger scale production of Fe‐MOFs is an important limitation for their widespread clinical application. Additionally, some of the largest pore size Fe‐MOFs degrade too fast in body fluids conditions, unless sophisticated coatings are implemented, which calls for new more robust sustainable Fe‐MOFs.

The ongoing research into Fe‐MOFs is poised to further significantly expand their biomedical applications. These versatile Fe‐MOF–based composites are likely to play a pivotal role in addressing some of the most pressing health challenges in the 21st century, particularly by providing solutions to diseases that current medical tools cannot effectively manage. Should the field of research continue to progress in the anticipated direction, it is plausible that Fe‐MOFs will strongly impact the landscape of disease treatment, opening new avenues for addressing complex medical challenges with flexibility and efficiency.

## Conflict of Interest

The authors declare no conflict of interest.

## References

[1] I. Abánades Lázaro , X. Chen , M. Ding , A. Eskandari , D. Fairen‐Jimenez , M. Giménez‐Marqués , R. Gref , W. Lin , T. Luo , R. S. Forgan , Nat. Rev. Methods Primers 2024, 4, 42.

[2] E. Linnane , S. Haddad , F. Melle , Z. Mei , D. Fairen‐Jimenez , Chem. Soc. Rev. 2022, 51, 6065. 35770998 10.1039/d0cs01414a PMC9289890

[3] Y. Lu , H. Zhou , H. Yang , Z. Zhou , Z. Jiang , H. Pang , J. Mater. Chem. A. 2024, 12, 6243.

[4] J. Jiang , Y. Shi , M. Wu , M. Rezakazemi , T. M. Aminabhavi , R. Huang , C. Jia , S. Ge , Chem. Eng. J. 2024, 494, 152932.

[5] S. Daliran , A. R. Oveisi , C.‐W. Kung , U. Sen , A. Dhakshinamoorthy , C.‐H. Chuang , M. Khajeh , M. Erkartal , J. T. Hupp , Chem. Soc. Rev. 2024, 53, 6244. 38743011 10.1039/d3cs01057k

[6] A. Dhakshinamoorthy , Z. Li , S. Yang , H. Garcia , Chem. Soc. Rev. 2024, 53, 3002. 38353930 10.1039/d3cs00205e

[7] D. Han , X. Liu , S. Wu , Chem. Soc. Rev. 2022, 51, 7138. 35866702 10.1039/d2cs00460g

[8] W. Zou , L. Zhang , J. Lu , D. Sun , Chem. Eng. J. 2024, 480, 148220.

[9] Y. Xiong , Q. Feng , L. Lu , X. Qiu , S. Knoedler , A. C. Panayi , D. Jiang , Y. Rinkevich , Z. Lin , B. Mi , G. Liu , Y. Zhao , Adv. Mater. 2024, 36, 2302587. 10.1002/adma.202302587 37527058

[10] J. Zhao , Y. Zhang , Y. Luo , W. Zheng , X. Xu , F. Luo , Chem. Eng. J. 2024, 492, 152295.

[11] A. Wang , M. Walden , R. Ettlinger , F. Kiessling , J. J. Gassensmith , T. Lammers , S. Wuttke , Q. Peña , Adv. Funct. Mater. 2023, 2308589. 10.1002/adfm.202308589 PMC7617264 39726715

[12] M. Koshy , M. Spiotto , L. E. Feldman , J. J. Luke , G. F. Fleming , D. Olson , J. W. Moroney , R. Nanda , A. Rosenberg , A. T. Pearson , A. Juloori , F. Weinberg , C. Ray , R. C. Gaba , P. J. Chang , L. A. Janisch , Z.‐Q. Xu , W. Lin , R. R. Weichselbaum , S. J. Chmura , J. Clin. Orthod. 2023, 41, 2527.

[13] D. Cattaneo , S. J. Warrender , M. J. Duncan , C. J. Kelsall , M. K. Doherty , P. D. Whitfield , I. L. Megson , R. E. Morris , RSC Adv. 2016, 6, 14059. 27019705 10.1039/c5ra24023a PMC4786954

[14] B. Xiao , P. J. Byrne , P. S. Wheatley , D. S. Wragg , X. Zhao , A. J. Fletcher , K. M. Thomas , L. Peters , J. S. O. Evans , J. E. Warren , W. Zhou , R. E. Morris , Nat. Chem. 2009, 1, 289. 21495253 10.1038/nchem.254

[15] X. Peng , L. Xu , M. Zeng , H. Dang , Int. J. Nanomed. 2023, 18, 4907. 10.2147/IJN.S417543 PMC10479543 37675409

[16] H. Yang , D. Liao , Z. Cai , Y. Zhang , A. Nezamzadeh‐Ejhieh , M. Zheng , J. Liu , Z. Bai , H. Song , RSC Med. Chem. 2023, 14, 2473. 38107167 10.1039/d3md00416c PMC10718519

[17] R. Zhu , M. Cai , T. Fu , D. Yin , H. Peng , S. Liao , Y. Du , J. Kong , J. Ni , X. Yin , Pharmaceutics 2023, 15, 1599. 37376050 10.3390/pharmaceutics15061599 PMC10301457

[18] Y. Zhong , W. Liu , C. Rao , B. Li , X. Wang , D. Liu , Y. Pan , J. Liu , Curr. Med. Chem. 2021, 28, 6179. 33992053 10.2174/0929867328666210511014129

[19] R. Kudarha , N. Dhas , S. Mutalik , Coord. Chem. Rev. 2023, 494, 215330.

[20] P. Horcajada , R. Gref , T. Baati , P. K. Allan , G. Maurin , P. Couvreur , G. Férey , R. E. Morris , C. Serre , Chem. Rev. 2012, 112, 1232. 22168547 10.1021/cr200256v

[21] R. Ettlinger , U. Lächelt , R. Gref , P. Horcajada , T. Lammers , C. Serre , P. Couvreur , R. E. Morris , S. Wuttke , Chem. Soc. Rev. 2022, 51, 464. 34985082 10.1039/d1cs00918d

[22] P. P. Fu , Q. Xia , H.‐M. Hwang , P. C. Ray , H. Yu , J. Food Drug Anal 2014, 22, 64. 24673904 10.1016/j.jfda.2014.01.005 PMC9359151

[23] R. L. Auten , J. M. Davis , Pediatr. Res. 2009, 66, 121. 19390491 10.1203/PDR.0b013e3181a9eafb

[24] J.‐M. Moulis , BioMetals 2010, 23, 877. 20524046 10.1007/s10534-010-9336-y

[25] Z. Wang , B. Liu , Q. Sun , L. Feng , F. He , P. Yang , S. Gai , Z. Quan , J. Lin , ACS Nano 2021, 15, 12342. 34160201 10.1021/acsnano.1c04280

[26] A. B. G. Lansdown , Adv. Pharmacol. Pharm Sci. 2010, 2010, e910686. 10.1155/2010/910686 PMC3003978 21188244

[27] R. A. Goyer , T. W. Clarkson , Toxic effects of metals, In C. D. Klaassen , ed, Casarett and Doull's Toxicology: The Basic Science of Poisons, McGraw‐Hill, New York, NY, USA, 2001, pp 811–868.

[28] K. Sule , J. Umbsaar , E. J. Prenner , Biochim. Biophys. Acta Biomembr. 2020, 1862, 183250. 32126229 10.1016/j.bbamem.2020.183250

[29] S. Rojas , A. Arenas‐Vivo , P. Horcajada , Coord. Chem. Rev. 2019, 388, 202.

[30] K. M. de la Harpe , P. P. D. Kondiah , Y. E. Choonara , T. Marimuthu , L. C. du Toit , V. Pillay , Cells 2019, 8, 1209. 31591302 10.3390/cells8101209 PMC6829615

[31] C. Tamames‐Tabar , D. Cunha , E. Imbuluzqueta , F. Ragon , C. Serre , M. J. Blanco‐Prieto , P. Horcajada , J. Mater. Chem. B 2013, 2, 262. 32261505 10.1039/c3tb20832j

[32] X. Ma , Z. Yu , F. Nouar , I. Dovgaliuk , G. Patriarche , N. Sadovnik , M. Daturi , J.‐M. Grenèche , M. Lepoitevin , C. Serre , Chem. Mater. 2024, 36, 167.

[33] R. G. Pearson , J. Am. Chem. Soc. 1963, 85, 3533.

[34] R. G. Pearson , Science 1966, 151, 172.17746330

[35] M. J. de Velásquez‐Hernández , M. Linares‐Moreau , E. Astria , F. Carraro , M. Z. Alyami , N. M. Khashab , C. J. Sumby , C. J. Doonan , P. Falcaro , Coord. Chem. Rev. 2021, 429, 213651.

[36] E. Bellido , M. Guillevic , T. Hidalgo , M. J. Santander‐Ortega , C. Serre , P. Horcajada , Langmuir 2014, 30, 5911. 24801765 10.1021/la5012555

[37] I. Christodoulou , P. Lyu , C. V. Soares , G. Patriarche , C. Serre , G. Maurin , R. Gref , Int. J. Mol. Sci. 2023, 24, 3362. 36834775 10.3390/ijms24043362 PMC9965190

[38] A. Dasgupta , A. M. Sofias , F. Kiessling , T. Lammers , Nat. Rev. Bioeng. 2024, 2, 714. 39376248 10.1038/s44222-024-00203-3 PMC7616668

[39] I. V. Zelepukin , O. Y. Griaznova , K. G. Shevchenko , A. V. Ivanov , E. V. Baidyuk , N. B. Serejnikova , A. B. Volovetskiy , S. M. Deyev , A. V. Zvyagin , Nat. Commun. 2022, 13, 6910. 36376302 10.1038/s41467-022-34718-3 PMC9661469

[40] L. Luan , L. Du , W. Shi , Y. Li , Q. Zhang , Molecules 2022, 27, 8908. 36558041 10.3390/molecules27248908 PMC9781904

[41] N. T. T. Nguyen , T. T. T. Nguyen , S. Ge , R. K. Liew , D. T. C. Nguyen , T. Van Tran , Nanoscale Adv. 2024, 6, 1800. 38545292 10.1039/d3na01075a PMC10964756

[42] F. Nouar , C. Serre , M. L. Pinto , M. I. Severino , V. Pimenta , C. Freitas , ChemRxiv, 2023. 10.1021/acs.iecr.4c02618 PMC11803623 39926435

[43] I. Christodoulou , T. Bourguignon , X. Li , G. Patriarche , C. Serre , C. Marlière , R. Gref , Nanomaterials 2021, 11, 722. 33805652 10.3390/nano11030722 PMC8001454

[44] Z. Mo , X. Pan , X. Pan , L. Ye , H. Hu , Q. Xu , X. Hu , Z. Xu , J. Xiong , G. Liao , S. Yang , J. Mater. Chem. B 2022, 10, 8760. 36255232 10.1039/d2tb01691e

[45] I. Y. Skobelev , A. B. Sorokin , K. A. Kovalenko , V. P. Fedin , O. A. Kholdeeva , J. Catal. 2013, 298, 61.

[46] M. Almáši , V. Zeleňák , P. Palotai , E. Beňová , A. Zeleňáková , Inorg. Chem. Commun. 2018, 93, 115.

[47] T. Devic , P. Horcajada , C. Serre , F. Salles , G. Maurin , B. Moulin , D. Heurtaux , G. Clet , A. Vimont , J.‐M. Grenèche , B. L. Ouay , F. Moreau , E. Magnier , Y. Filinchuk , J. Marrot , J.‐C. Lavalley , M. Daturi , G. Férey , J. Am. Chem. Soc. 2010, 132, 1127. 20038143 10.1021/ja9092715

[48] S. Devautour‐Vinot , G. Maurin , F. Henn , C. Serre , T. Devic , G. Férey , Chem. Commun. 2009, 2733. 10.1039/b822834e 19532937

[49] J.‐W. Zhang , H.‐T. Zhang , Z.‐Y. Du , X. Wang , S.‐H. Yu , H.‐L. Jiang , Chem. Commun. 2013, 50, 1092. 10.1039/c3cc48398c 24317416

[50] T. Chalati , P. Horcajada , R. Gref , P. Couvreur , C. Serre , J. Mater. Chem. 2011, 21, 2220. 10.2217/nnm.11.69 22122581

[51] V. P. Viswanathan , S. V. Mathew , D. P. Dubal , N. N. Adarsh , S. Mathew , ChemistrySelect 2020, 5, 7534.

[52] M. Ma , A. Bétard , I. Weber , N. S. Al‐Hokbany , R. A. Fischer , N. Metzler‐Nolte , Cryst. Growth Des. 2013, 13, 2286.

[53] C. Serre , F. Millange , S. Surblé , G. Férey , Angew. Chem., Int. Ed. 2004, 43, 6285. 10.1002/anie.200454250 15372643

[54] D. E. Al‐Ansari , M. Al‐Badr , Z. Z. Zakaria , N. A. Mohamed , G. K. Nasrallah , H. C. Yalcin , H. Abou‐Saleh , Toxicol Rep. 2022, 9, 951. 35875258 10.1016/j.toxrep.2022.04.016 PMC9301604

[55] H. Chevreau , A. Permyakova , F. Nouar , P. Fabry , C. Livage , F. Ragon , A. Garcia‐Marquez , T. Devic , N. Steunou , C. Serre , P. Horcajada , CrystEngComm 2016, 18, 4094.

[56] P. Horcajada , T. Chalati , C. Serre , B. Gillet , C. Sebrie , T. Baati , J. F. Eubank , D. Heurtaux , P. Clayette , C. Kreuz , J.‐S. Chang , Y. K. Hwang , V. Marsaud , P.‐N. Bories , L. Cynober , S. Gil , G. Férey , P. Couvreur , R. Gref , Nat. Mater. 2010, 9, 172. 20010827 10.1038/nmat2608

[57] T. Baati , L. Njim , F. Neffati , A. Kerkeni , M. Bouttemi , R. Gref , M. F. Najjar , A. Zakhama , P. Couvreur , C. Serre , P. Horcajada , Chem. Sci. 2013, 4, 1597.

[58] R. Grall , T. Hidalgo , J. Delic , A. Garcia‐Marquez , S. Chevillard , P. Horcajada , J. Mater. Chem. B 2015, 3, 8279. 32262883 10.1039/c5tb01223f

[59] Q. Xia , H. Wang , B. Huang , X. Yuan , J. Zhang , J. Zhang , L. Jiang , T. Xiong , G. Zeng , Small 2019, 15, 1803088. 10.1002/smll.201803088 30548176

[60] V. Agostoni , T. Chalati , P. Horcajada , H. Willaime , R. Anand , N. Semiramoth , T. Baati , S. Hall , G. Maurin , H. Chacun , K. Bouchemal , C. Martineau , F. Taulelle , P. Couvreur , C. Rogez‐Kreuz , P. Clayette , S. Monti , C. Serre , R. Gref , Adv. Healthcare Mater. 2013, 2, 1630. 10.1002/adhm.201200454 23776182

[61] X. Li , L. Lachmanski , S. Safi , S. Sene , C. Serre , J. M. Grenèche , J. Zhang , R. Gref , Sci. Rep. 2017, 7, 13142. 29030570 10.1038/s41598-017-13323-1 PMC5640595

[62] M. D. L. Vuong , Y. Horbenko , M. Frégnaux , I. Christodoulou , C. Martineau‐Corcos , P. Levitz , A.‐L. Rollet , R. Gref , M. Haouas , ACS Appl. Mater. Interfaces 2024, 16, 2086. 38166380 10.1021/acsami.3c14301

[63] Y. Zhang , C. Liu , F. Wang , Z. Liu , J. Ren , X. Qu , Chem. Commun. 2017, 53, 1840. 10.1039/c6cc09280b 28111662

[64] Q. Wang , X. Zhu , X. Meng , H. Zhong , Acta Biomater. 2023, 172, 382. 37797707 10.1016/j.actbio.2023.09.052

[65] H. Zhao , S. Sene , A. M. Mielcarek , S. Miraux , N. Menguy , D. Ihiawakrim , O. Ersen , C. Péchoux , N. Guillou , J. Scola , J.‐M. Grenèche , F. Nouar , S. Mura , F. Carn , F. Gazeau , E. Dumas , C. Serre , N. Steunou , J. Mater. Chem. B 2023, 11, 3195. 36951043 10.1039/d2tb02094g

[66] B. R. Stockwell , J. P. F. Angeli , H. Bayir , A. I. Bush , M. Conrad , S. J. Dixon , S. Fulda , S. Gascón , S. K. Hatzios , V. E. Kagan , K. Noel , X. Jiang , A. Linkermann , M. E. Murphy , M. Overholtzer , A. Oyagi , G. C. Pagnussat , J. Park , Q. Ran , C. S. Rosenfeld , K. Salnikow , D. Tang , F. M. Torti , S. V. Torti , S. Toyokuni , K. A. Woerpel , D. D. Zhang , Cell 2017, 171, 273. 28985560 10.1016/j.cell.2017.09.021 PMC5685180

[67] S. J. Dixon , K. M. Lemberg , M. R. Lamprecht , R. Skouta , E. M. Zaitsev , C. E. Gleason , D. N. Patel , A. J. Bauer , A. M. Cantley , W. S. Yang , B. Morrison , B. R. Stockwell , Cell 2012, 149, 1060. 22632970 10.1016/j.cell.2012.03.042 PMC3367386

[68] W. S. Yang , R. SriRamaratnam , M. E. Welsch , K. Shimada , R. Skouta , V. S. Viswanathan , J. H. Cheah , P. A. Clemons , A. F. Shamji , C. B. Clish , L. M. Brown , A. W. Girotti , V. W. Cornish , S. L. Schreiber , B. R. Stockwell , Cell 2014, 156, 317. 24439385 10.1016/j.cell.2013.12.010 PMC4076414

[69] Y. Xie , X. Song , X. Sun , J. Huang , M. Zhong , M. T. Lotze , H. J. Zeh , R. Kang , D. Tang , Biochem. Biophys. Res. Commun. 2016, 473, 775. 27037021 10.1016/j.bbrc.2016.03.052

[70] M. Gao , J. Yi , J. Zhu , A. M. Minikes , P. Monian , C. B. Thompson , X. Jiang , Mol. Cell 2019, 73, 354. 30581146 10.1016/j.molcel.2018.10.042 PMC6338496

[71] J. M. Ubellacker , A. Tasdogan , V. Ramesh , B. Shen , E. C. Mitchell , M. S. Martin‐Sandoval , Z. Gu , M. L. McCormick , A. B. Durham , D. R. Spitz , Z. Zhao , T. P. Mathews , S. J. Morrison , Nature 2020, 585, 113. 32814895 10.1038/s41586-020-2623-z PMC7484468

[72] N. Kang , S. Son , S. Min , H. Hong , C. Kim , J. An , J. S. Kim , H. Kang , Chem. Soc. Rev. 2023, 52, 3955. 37218295 10.1039/d3cs00001j

[73] L. Shao , T. Hu , X. Fan , X. Wu , F. Zhou , B. Chen , S. Tan , H. Xu , A. Pan , S. Liang , Y. He , ACS Appl. Mater. Interfaces 2022, 14, 13122. 35286061 10.1021/acsami.2c01913

[74] L. Jiang , N. Kon , T. Li , S.‐J. Wang , T. Su , H. Hibshoosh , R. Baer , W. Gu , Nature 2015, 520, 57. 25799988 10.1038/nature14344 PMC4455927

[75] H. Hou , X. Huang , G. Wei , F. Xu , Y. Wang , S. Zhou , ACS Appl. Mater. Interfaces 2019, 11, 29579. 31359756 10.1021/acsami.9b09671

[76] Y. Zheng , J. Zheng , M. Du , Y. Yang , X. Li , H. Chen , Y. Gao , J. Mater. Chem. B 2023, 11, 4958. 37203438 10.1039/d3tb00029j

[77] R. Song , T. Li , J. Ye , F. Sun , B. Hou , M. Saeed , J. Gao , Y. Wang , Q. Zhu , Z. Xu , H. Yu , Adv. Mater. 2021, 33, 2101155. 10.1002/adma.202101155 34170581

[78] M. Kuzu , F. M. Kandemir , S. Yildirim , S. Kucukler , C. Caglayan , E. Turk , Biomed. Pharmacother. 2018, 106, 443. 29990832 10.1016/j.biopha.2018.06.161

[79] L.‐S. Lin , T. Huang , J. Song , X.‐Y. Ou , Z. Wang , H. Deng , R. Tian , Y. Liu , J.‐F. Wang , Y. Liu , G. Yu , Z. Zhou , S. Wang , G. Niu , H.‐H. Yang , X. Chen , J. Am. Chem. Soc. 2019, 141, 9937. 31199131 10.1021/jacs.9b03457

[80] S. Stentzel , H. C. Vu , A. M. Weyrich , N. Jehmlich , F. Schmidt , M. G. Salazar , L. Steil , U. Völker , B. M. Bröker , Proteomics 2014, 14, 1857. 24888718 10.1002/pmic.201300512

[81] M. C. del Parquet , K. A. Savage , D. S. Allan , R. J. Davidson , B. E. Holbein , Front Microbiol 2018, 9, 1811. 30154764 10.3389/fmicb.2018.01811 PMC6103240

[82] K. A. Savage , M. C. del Parquet , D. S. Allan , R. J. Davidson , B. E. Holbein , E. A. Lilly , P. L. Fidel , Antimicrob. Agents Chemother. 2018, 62, e02576. 29844048 10.1128/AAC.02576-17 PMC6105849

[83] G. V. Smirnova , O. N. Oktyabrsky , Biochemistry 2005, 70, 1199. 16336178 10.1007/s10541-005-0248-3

[84] L. Wongsaroj , K. Saninjuk , A. Romsang , J. Duang‐nkern , W. Trinachartvanit , P. Vattanaviboon , S. Mongkolsuk , PLoS One 2018, 13, e0205815. 30325949 10.1371/journal.pone.0205815 PMC6191110

[85] M. B. Toledano , C. Kumar , N. Le Moan , D. Spector , F. Tacnet , FEBS Lett. 2007, 581, 3598. 17659286 10.1016/j.febslet.2007.07.002

[86] G. L. Newton , R. C. Fahey , M. Rawat , Microbiology 2012, 158, 1117. 22262099 10.1099/mic.0.055715-0 PMC3949421

[87] R. C. Fahey , Biochimica et Biophysica Acta (BBA) – General Subjects 2013, 1830, 3182. 23075826 10.1016/j.bbagen.2012.10.006

[88] K. Van Laer , L. Buts , N. Foloppe , D. Vertommen , K. Van Belle , K. Wahni , G. Roos , L. Nilsson , L. M. Mateos , M. Rawat , N. A. J. van Nuland , J. Messens , Mol. Microbiol. 2012, 86, 787. 22970802 10.1111/mmi.12030

[89] R. Ma , L. Fang , L. Chen , X. Wang , J. Jiang , L. Gao , Theranostics 2022, 12, 2266. 35265210 10.7150/thno.66663 PMC8899587

[90] Z. Wang , H. Li , W. Zhou , J. Lee , Z. Liu , Z. An , D. Xu , H. Mo , L. Hu , X. Zhou , Biomaterials 2022, 290, 121842. 36206665 10.1016/j.biomaterials.2022.121842

[91] P. Horcajada , C. Serre , G. Maurin , N. A. Ramsahye , F. Balas , M. Vallet‐Regí , M. Sebban , F. Taulelle , G. Férey , J. Am. Chem. Soc. 2008, 130, 6774. 18454528 10.1021/ja710973k

[92] S. Surblé , C. Serre , C. Mellot‐Draznieks , F. Millange , G. Férey , Chem. Commun. 2006, 284. 10.1039/b512169h 16391735

[93] P. Horcajada , S. Surblé , C. Serre , D.‐Y. Hong , Y.‐K. Seo , J.‐S. Chang , J.‐M. Grenèche , I. Margiolaki , G. Férey , Chem. Commun. 2007, 2820. 10.1039/b704325b 17609787

[94] K. M. L. Taylor‐Pashow , J. D. Rocca , Z. Xie , S. Tran , W. Lin , J. Am. Chem. Soc. 2009, 131, 14261. 19807179 10.1021/ja906198y PMC2760011

[95] H. Peng , X. Zhang , P. Yang , J. Zhao , W. Zhang , N. Feng , W. Yang , J. Tang , Bioact. Mater. 2023, 19, 1. 35415315 10.1016/j.bioactmat.2021.12.018 PMC8980498

[96] J. Du , M. Zhou , Q. Chen , Y. Tao , J. Ren , Y. Zhang , H. Qin , Adv. Funct. Mater. 2023, 33, 2215244.

[97] Y. Ye , H. Yu , B. Chen , Y. Zhao , B. Lv , G. Xue , Y. Sun , J. Cao , J. Colloid Interface Sci. 2023, 645, 882. 37178565 10.1016/j.jcis.2023.05.003

[98] G. Shu , H. Zhao , X. Zhang , Biomater. Sci. 2023, 11, 1797. 36655655 10.1039/d2bm01920e

[99] C. Yang , Z. Chen , M. Wei , S. Hu , M. Cai , N. Wang , Y. Guan , F. Li , Q. Ding , D. Ling , J. Controlled Release 2023, 357, 20. 10.1016/j.jconrel.2023.03.030 36940774

[100] X.‐X. Yang , X. Xu , M.‐F. Wang , H.‐Z. Xu , X.‐C. Peng , N. Han , T.‐T. Yu , L.‐G. Li , Q.‐R. Li , X. Chen , Y. Wen , T.‐F. Li , J. Nanobiotechnol. 2022, 20, 230. 10.1186/s12951-022-01455-0 PMC9107746 35568865

[101] X. Song , Q. Jiang , J. Ma , Y. Liu , L. Zhang , T. Jiang , J. Zhang , Q. Li , J. Sun , Ceram. Int. 2024, 50, 7486.

[102] X. Li , G. Li , Q. Pan , F. Xue , Z. Wang , C. Peng , Biosens. Bioelectron. 2024, 250, 116044. 38271888 10.1016/j.bios.2024.116044

[103] Y. Liu , X.‐Z. Meng , X. Luo , H.‐W. Gu , X.‐L. Yin , W.‐L. Han , H.‐C. Yi , Y. Chen , Sens. Actuators, B 2024, 410, 135682.

[104] M. U. Akbar , A. Akbar , U. A. K. Saddozai , M. I. U. Khan , M. Zaheer , M. Badar , Mater. Adv. 2023, 4, 5653.

[105] Y.‐K. Seo , J. W. Yoon , J. S. Lee , U.‐H. Lee , Y. K. Hwang , C.‐H. Jun , P. Horcajada , C. Serre , J.‐S. Chang , Microporous Mesoporous Mater. 2012, 157, 137.

[106] M. Oveisi , N. M. Mahmoodi , M. A. Asli , J. Cleaner Prod. 2019, 222, 669.

[107] T. Steenhaut , S. Hermans , Y. Filinchuk , New J. Chem. 2020, 44, 3847.

[108] B. Yuan , X. Wang , X. Zhou , J. Xiao , Z. Li , Chem. Eng. J. 2019, 355, 679.

[109] K. Guesh , C. A. D. Caiuby , Á. Mayoral , M. Díaz‐García , I. Díaz , M. Sanchez‐Sanchez , Cryst. Growth Des. 2017, 17, 1806.

[110] W. Li , T. Zhang , L. Lv , Y. Chen , W. Tang , S. Tang , Colloids Surf. A. 2021, 624, 126791.

[111] M. Panchal , F. Nouar , C. Serre , M. Benzaqui , S. Sene , N. Steunou , M. G. Marqués , EP3357929A1, 2018.

[112] P. Dinh Du , P. N. Hoai , Adv. Mater. Sci. Eng. 2021, 2021, e5540344.

[113] Y. Jin , Y. Li , Q. Du , B. Chen , K. Chen , Y. Zhang , M. Wang , Y. Sun , S. Zhao , Z. Jing , J. Wang , X. Pi , Y. Wang , Microporous Mesoporous Mater. 2023, 348, 112404.

[114] S. H. Jhung , J.‐H. Lee , J. W. Yoon , C. Serre , G. Férey , J.‐S. Chang , Adv. Mater. 2007, 19, 121.

[115] J. Amaro‐Gahete , R. Klee , D. Esquivel , J. R. Ruiz , C. Jiménez‐Sanchidrián , F. J. Romero‐Salguero , Ultrason. Sonochem. 2019, 50, 59. 30219350 10.1016/j.ultsonch.2018.08.027

[116] D. Chakraborty , A. Yurdusen , G. Mouchaham , F. Nouar , C. Serre , Adv. Funct. Mater. 2023, 2309089.

[117] M. Samal , J. Panda , B. P. Biswal , R. Sahu , CrystEngComm 2018, 20, 2486.

[118] J.‐L. Do , T. Friščić , ACS Cent. Sci. 2017, 3, 13. 28149948 10.1021/acscentsci.6b00277 PMC5269651

[119] W. Wang , M. Chai , M. Y. B. Zulkifli , K. Xu , Y. Chen , L. Wang , V. Chen , J. Hou , Mol. Syst. Des. Eng. 2023, 8, 560.

[120] J. Troyano , C. Çamur , L. Garzón‐Tovar , A. Carné‐Sánchez , I. Imaz , D. Maspoch , Acc. Chem. Res. 2020, 53, 1206. 32496790 10.1021/acs.accounts.0c00133

[121] L. Garzón‐Tovar , M. Cano‐Sarabia , A. Carné‐Sánchez , C. Carbonell , I. Imaz , D. Maspoch , React. Chem. Eng. 2016, 1, 533.

[122] A. Carné‐Sánchez , I. Imaz , M. Cano‐Sarabia , D. Maspoch , Nat. Chem. 2013, 5, 203. 23422562 10.1038/nchem.1569

[123] M. P. Abuçafy , R. C. G. Frem , G. Polinario , F. R. Pavan , H. Zhao , A. Mielcarek , C. Boissiere , C. Serre , L. A. Chiavacci , Int. J. Mol. Sci. 2022, 23, 7670. 35887018 10.3390/ijms23147670 PMC9324886

[124] Y.‐P. He , Y.‐X. Tan , J. Zhang , Inorg. Chem. 2012, 51, 11232. 23043487 10.1021/ic3017529

[125] C. R. Quijia , A. Ocaña , C. AlonsoMoreno , R. C. Galvão Frem , M. Chorilli , J. Mol. Struct. 2024, 1305, 137801.

[126] T. Hidalgo , M. Giménez‐Marqués , E. Bellido , J. Avila , M. C. Asensio , F. Salles , M. V. Lozano , M. Guillevic , R. Simón‐Vázquez , A. González‐Fernández , C. Serre , M. J. Alonso , P. Horcajada , Sci. Rep. 2017, 7, 43099. 28256600 10.1038/srep43099 PMC5335263

[127] L. Cui , X. Wang , Z. Liu , Z. Li , Z. Bai , K. Lin , J. Yang , Y. Cui , F. Tian , Int. J. Biol. Macromol. 2023, 240, 124370. 37044320 10.1016/j.ijbiomac.2023.124370

[128] Y. Wang , Z. Chen , J. Li , Y. Wen , J. Li , Y. Lv , Z. Pei , Y. Pei , Adv. Sci. 2024, 11, 2306178. 10.1002/advs.202306178 PMC10953551 38161219

[129] P. Zhang , J. Fu , J. Hu , Q. You , X. Yao , D. Hua , J. Yin , Y. Mao , Chem. Eng. J. 2023, 454, 140044.

[130] L. Yang , H. Chen , S. Zhu , S. Zhao , S. Huang , D. Cheng , H. Xu , Z. Zhang , ACS Nano 2024, 18, 6533. 38355215 10.1021/acsnano.3c12352

[131] A. D. Sontakke , S. Tiwari , P. Gupta , S. K. Banerjee , M. K. Purkait , Mater. Today Commun. 2024, 38, 108560.

[132] M. Ding , J. Qiu , S. Rouzière , C. Rihouey , L. Picton , R. Gref , Int. J. Mol. Sci. 2023, 24, 1757. 36675274 10.3390/ijms24021757 PMC9866736

[133] W. J. Chen , D. Gupta , M. Yang , F. Yang , N. Feng , J. Song , M. J. A. Wood , L. Qiu , J. Chen , Small 2023, 19, 2303403. 10.1002/smll.202303403 37649230

[134] X. Zheng , X. Li , S. Meng , G. Shi , H. Li , H. Du , L. Dai , H. Yang , J. Nanobiotechnol. 2023, 21, 127. 10.1186/s12951-023-01878-3 PMC10088258 37041537

[135] Z. Feng , G. Chen , M. Zhong , L. Lin , Z. Mai , Y. Tang , G. Chen , W. Ma , G. Li , Y. Yang , Z. Yu , M. Yu , Biomaterials 2023, 302, 122333. 37738743 10.1016/j.biomaterials.2023.122333

[136] L. Chen , D. Zhao , X. Ren , J. Ren , X. Meng , C. Fu , X. Li , ACS Biomater. Sci. Eng. 2023, 9, 5405. 37638660 10.1021/acsbiomaterials.3c00644 PMC10498989

[137] Y. Chen , L. Gu , B. Ma , X. Li , Y. Mei , J. Zhou , Y. Chong , M. Ma , M. Zhang , L. Wang , Y. Cheng , K. Wu , J. Zeng , M. Cheng , P. Guo , P. Zhang , D. He , Chem. Eng. J. 2023, 454, 140438.

[138] G. An , H. Zheng , L. Guo , J. Huang , C. Yang , Z. Bai , N. Wang , W. Yang , Y. Zhu , J. Colloid Interface Sci. 2024, 662, 298. 38354557 10.1016/j.jcis.2024.02.055

[139] C. Ou , F. Yang , M. Gong , C. Xu , S. Yang , J. Yang , W. Qi , Y. Luo , Z. Peng , L. Deng , D. He , ACS Appl. Nano Mater. 2023, 6, 15860.

[140] M. Zhang , X. Yao , J. Xu , J. Song , S. Mai , W. Zhu , Y. Zhang , L. Zhu , W. Yang , Int. J. Pharm. 2024, 655, 124032. 38521374 10.1016/j.ijpharm.2024.124032

[141] M. Supianto , D. K. Yoo , H. Hwang , H. B. Oh , S. H. Jhung , H. J. Lee , ACS Sens. 2024, 9, 1321. 38471126 10.1021/acssensors.3c02250

[142] Y.‐S. Yu , C.‐H. Hsu , P.‐H. Cheng , K. C.‐W. Wu , C.‐H. Liu , Mater. Chem. Phys. 2022, 292, 126840.

[143] S. Wang , Q. Guo , R. Xu , P. Lin , G. Deng , X. Xia , J. Nanobiotechnol. 2023, 21, 383. 10.1186/s12951-023-02146-0 PMC10585872 37858186

[144] G. Li , X. Lu , S. Zhang , J. Zhang , X. Fu , M. Zhang , L. Teng , F. Sun , ACS Appl. Mater. Interfaces 2023, 15, 31285. 37344958 10.1021/acsami.3c05337

[145] M. Giménez‐Marqués , E. Bellido , T. Berthelot , T. Simón‐Yarza , T. Hidalgo , R. Simón‐Vázquez , Á. González‐Fernández , J. Avila , M. C. Asensio , R. Gref , P. Couvreur , C. Serre , P. Horcajada , Small 2018, 14, 1801900. 10.1002/smll.201801900 30091524

[146] A. Zimpel , T. Preiß , R. Röder , H. Engelke , M. Ingrisch , M. Peller , J. O. Rädler , E. Wagner , T. Bein , U. Lächelt , S. Wuttke , Chem. Mater. 2016, 28, 3318.

[147] R. Liang , F. Li , X. Chen , F. Tan , T. Lan , J. Yang , J. Liao , Y. Yang , N. Liu , ACS Appl. Mater. Interfaces 2023, 15, 45713. 37738473 10.1021/acsami.3c11098

[148] I. A. Lázaro , M. Vicent‐Morales , G. M. Espallargas , M. Giménez‐Marqués , J. Mater. Chem. B 2023, 11, 9179. 37718709 10.1039/d3tb01819a

[149] X. Tang , J. Tang , Q. Zhang , D. Suonanmu , Y. Zhang , Q. Ren , F. Tao , C. Li , F. Wang , Sens. Actuators, B 2024, 403, 135176.

[150] J. Dong , T. Li , H. Yan , Y. Chen , C. Wang , X. Luan , X. Li , H. Li , X. Du , Colloids Surf. A 2024, 683, 133101.

[151] Z. Rao , Y. Xia , Q. Jia , Y. Zhu , L. Wang , G. Liu , X. Liu , P. Yang , P. Ning , R. Zhang , X. Zhang , C. Qiao , Z. Wang , J. Nanobiotechnol. 2023, 21, 265. 10.1186/s12951-023-01998-w PMC10416514 37563614

[152] T. Cui , Y. Zhang , G. Qin , Y. Wei , J. Yang , Y. Huang , J. Ren , X. Qu , Nat. Commun. 2023, 14, 1974. 37031242 10.1038/s41467-023-37580-z PMC10082843

[153] P. Graván , S. Rojas , D. F. Picchi , F. Galisteo‐González , P. Horcajada , J. A. Marchal , Nanomaterials 2024, 14, 784. 38727378 10.3390/nano14090784 PMC11085653

[154] Z. Bu , J. Yang , Y. Zhang , T. Luo , C. Fang , X. Liang , Q. Peng , D. Wang , N. Lin , K. Zhang , W. Tang , Adv. Sci. 2023, 10, 2301638. 10.1002/advs.202301638 PMC10427397 37303273

[155] Y. Luo , X. Yu , Y. Yao , L. Zhu , W. Qin , Z. Shan , L. Song , J. Wang , L. Wang , H. Yan , M. Chen , ACS Appl. Nano Mater. 2023, 6, 12744.

[156] J. Chen , Z. Zhu , Q. Pan , Y. Bai , M. Yu , Y. Zhou , Adv. Funct. Mater. 2023, 33, 2300235.

[157] Q. Zhang , G. Kuang , H. Wang , Y. Zhao , J. Wei , L. Shang , Adv. Sci. 2023, 10, 2303818. 10.1002/advs.202303818 PMC10667824 37852943

[158] A. A. Edlo , K. Akhbari , Appl. Organomet. Chem. 2024, 38, e7326.

[159] K. Li , Z. Yu , I. Dovgaliuk , C. L. Coeur , V. Lütz‐Bueno , E. Leroy , B. Brissault , Y. d. R. de Mimerand , M. Lepoitevin , C. Serre , J. Penelle , B. Couturaud , Chem. Commun. 2023, 59, 4923. 10.1039/d2cc06088d 37010849

[160] V. Agostoni , P. Horcajada , M. Noiray , M. Malanga , A. Aykaç , L. Jicsinszky , A. Vargas‐Berenguel , N. Semiramoth , S. Daoud‐Mahammed , V. Nicolas , C. Martineau , F. Taulelle , J. Vigneron , A. Etcheberry , C. Serre , R. Gref , Sci. Rep. 2015, 5, 7925. 25603994 10.1038/srep07925 PMC4300503

[161] J. Szejtli , Chem. Rev. 1998, 98, 1743.11848947

[162] A. Cheung , H. J. Bax , D. H. Josephs , K. M. Ilieva , G. Pellizzari , J. Opzoomer , J. Bloomfield , M. Fittall , A. Grigoriadis , M. Figini , S. Canevari , J. F. Spicer , A. N. Tutt , S. N. Karagiannis , OncoTargets Ther. 2016, 7, 52553. 10.18632/oncotarget.9651 PMC5239573 27248175

[163] H. Cheng , J.‐Y. Zhu , S.‐Y. Li , J.‐Y. Zeng , Q. Lei , K.‐W. Chen , C. Zhang , X.‐Z. Zhang , Adv. Funct. Mater. 2016, 26, 7847.

[164] Y. Teng , W. Li , J. Wang , S. Jia , H. Zhang , T. Yang , X. Li , L. Li , C. Wang , Sep. Purif. Technol. 2023, 315, 123610.

[165] L. K. Njaramba , Y. Yoon , C. M. Park , npj Clean Water 2024, 7, 14.

[166] F. O. Kirbay , İ. Yazgan , D. O. Demirkol , Sens. Actuators, B 2022, 372, 132621.

[167] M. R. Ramezani , Z. Ansari‐Asl , E. Hoveizi , A. R. Kiasat , Mater. Chem. Phys. 2019, 229, 242.

[168] P. Zhang , X. Xu , W. He , H. Li , Y. Huang , G. Wu , Nanomedicine: Nanotechnology, Biology and Medicine 2023, 51, 102683. 37105341 10.1016/j.nano.2023.102683

[169] H. Fu , H. Li , Q. He , G. Du , J. Li , Y. Zhang , Y. Liang , B. Lin , Chem. Eng. J. 2024, 490, 151879.

[170] P. Tignol , V. Pimenta , A.‐L. Dupont , S. Carvalho , A. A. Mohtar , M. Inês Severino , F. Nouar , M. L. Pinto , C. Serre , B. Lavédrine , Small Methods 2024, 8, 2301343. 10.1002/smtd.202301343 38032133

[171] C. Sun , C. Li , M. Guo , X. Yang , Y. Luo , L. Chen , H. Zheng , S. Zhao , F. Li , J. Hazard. Mater. 2023, 458, 131946. 37418967 10.1016/j.jhazmat.2023.131946

[172] S. Mansi , S. V. Dummert , G. J. Topping , M. Z. Hussain , C. Rickert , K. M. A. Mueller , T. Kratky , M. Elsner , A. Casini , F. Schilling , R. A. Fischer , O. Lieleg , P. Mela , Adv. Funct. Mater. 2024, 34, 2304907.

[173] M. Tian , L. Zhou , C. Fan , L. Wang , X. Lin , Y. Wen , L. Su , H. Dong , Acta Biomater. 2023, 158, 252. 36584802 10.1016/j.actbio.2022.12.049

[174] J. Chen , H. Niu , L. Guan , Z. Yang , Y. He , J. Zhao , C. Wu , Y. Wang , K. Lin , Y. Zhu , Adv. Healthcare Mater. 2023, 12, 2202474. 10.1002/adhm.202202474 36420881

[175] S. Miao , J. Guo , Z. Deng , J. Yu , Y. Dai , J. Cleaner Prod. 2022, 370, 133566.

[176] M. Abrigo , S. L. McArthur , P. Kingshott , Macromol. Biosci. 2014, 14, 772. 24678050 10.1002/mabi.201300561

[177] S. Zhang , J. Ye , X. Liu , Y. Wang , C. Li , J. Fang , B. Chang , Y. Qi , Y. Li , G. Ning , J. Colloid Interface Sci. 2021, 599, 390. 33962200 10.1016/j.jcis.2021.04.109

[178] J. Y. C. Lim , L. Goh , K. Otake , S. S. Goh , X. J. Loh , S. Kitagawa , Biomater. Sci. 2023, 11, 2661. 36810436 10.1039/d2bm01906j

[179] X. Li , T. T. Y. Tan , Q. Lin , C. C. Lim , R. Goh , K. Otake , S. Kitagawa , X. J. Loh , J. Y. C. Lim , ACS Biomater. Sci. Eng. 2023, 9, 5724. 37729089 10.1021/acsbiomaterials.3c01103

[180] J. K. Patra , G. Das , L. F. Fraceto , E. V. R. Campos , M. d. P. Rodriguez‐Torres , L. S. Acosta‐Torres , L. A. Diaz‐Torres , R. Grillo , M. K. Swamy , S. Sharma , S. Habtemariam , H.‐S. Shin , J. Nanobiotechnol. 2018, 16, 71. 10.1186/s12951-018-0392-8 PMC6145203 30231877

[181] P. Makvandi , R. Jamaledin , G. Chen , Z. Baghbantaraghdari , E. N. Zare , C. Di Natale , V. Onesto , R. Vecchione , J. Lee , F. R. Tay , P. Netti , V. Mattoli , A. Jaklenec , Z. Gu , R. Langer , Mater. Today 2021, 47, 206. 10.1016/j.mattod.2021.03.012 PMC9635273 36338772

[182] S. Yao , J. Chi , Y. Wang , Y. Zhao , Y. Luo , Y. Wang , Adv. Healthcare Mater. 2021, 10, 2100056. 10.1002/adhm.202100056 33938635

[183] Z. Li , L. Cao , J. Sui , L. Wang , H. Lin , K. Wang , Food Chem. 2024, 447, 138902. 38458132 10.1016/j.foodchem.2024.138902

[184] Y. Xia , J. He , L. Tang , M. Hu , J. Zhou , Y.‐Y. Xiao , Z.‐C. Jiang , X. Jiang , Food Chem X 2024, 21, 101247. 38434695 10.1016/j.fochx.2024.101247 PMC10907182

[185] X. Lin , J. Li , J. Wu , K. Guo , N. Duan , Z. Wang , S. Wu , ACS Appl. Mater. Interfaces 2024, 16, 11809. 38386848 10.1021/acsami.3c18878

[186] Y. Shu , Q. Ye , J. Tan , H. Lv , Z. Liu , Q. Mo , ACS Appl. Nano Mater. 2022, 5, 17909.

[187] B. Li , H. Wang , M. Liu , L. Geng , S. Dou , S. Zhai , J. Liu , J. Sun , W. Zhao , Y. Guo , X. Sun , Anal. Bioanal. Chem. 2024, 416, 1105. 38189917 10.1007/s00216-023-05104-9

[188] Z. Han , Q. Fu , Y. lv , N. Wang , X. Su , Talanta 2024, 272, 125704. 38359716 10.1016/j.talanta.2024.125704

[189] J. Hou , J. Wang , J. Han , J. Wang , D. Chao , Q. Dong , D. Fan , S. Dong , Biosens. Bioelectron. 2024, 249, 116035. 38244294 10.1016/j.bios.2024.116035

[190] Q. Cao , W. Xu , H. Lu , Q. Jia , Microchem. J. 2024, 199, 109946.

[191] L. Kong , F. Hong , P. Luan , Y. Chen , Y. Feng , M. Zhu , Food Chem. 2024, 438, 137631. 37983998 10.1016/j.foodchem.2023.137631

[192] Y. Li , G. Zhao , B. An , K. Xu , D. Wu , X. Ren , H. Ma , X. Liu , R. Feng , Q. Wei , Anal. Chem. 2024, 96, 4067. 38419337 10.1021/acs.analchem.3c04515

[193] X. Huang , H. Deng , X. Deng , L. Li , M. Wu , C. Huang , Y. Zhang , H. Zhao , Microchim. Acta 2024, 191, 111. 10.1007/s00604-024-06188-5 38252316

[194] Z. Shu , H. Hu , Z. Yuan , Y. Zou , Q. Zhang , Y. Wang , X. Liu , S. Duan , F. Pi , J. Wang , X. Liu , H. Dai , Microchim. Acta 2024, 191, 210. 10.1007/s00604-024-06281-9 38499672

[195] S. Zhang , Y. Zhang , H. Wang , Y. Wang , H. Ma , D. Wu , Z. F. Gao , D. Fan , X. Ren , Q. Wei , Anal. Chim. Acta 2024, 1298, 342407. 38462332 10.1016/j.aca.2024.342407

[196] N. Duan , Y. Chang , T. Su , X. Zhang , M. Lu , Z. Wang , S. Wu , Biosens. Bioelectron. 2024, 249, 116022. 38219468 10.1016/j.bios.2024.116022

[197] T. Cao , S. Li , X. Wang , Y. Sun , C. Luo , Talanta 2024, 267, 125144. 37699268 10.1016/j.talanta.2023.125144

[198] W. Jiang , W. Liang , C. Zhao , W. Lai , B. Cong , S. Zhang , M. Jiang , H. Li , C. Hong , Sens. Actuators, B 2024, 407, 135498.

[199] H.‐N. Sun , M. Wang , H.‐S. Tan , H.‐P. Liu , M. Liu , S.‐S. Li , Talanta 2024, 273, 125876. 38458082 10.1016/j.talanta.2024.125876

[200] Z. Cheng , G. He , R. Liao , Y. Tan , W. Deng , Bioelectrochemistry 2024, 157, 108677. 38430576 10.1016/j.bioelechem.2024.108677

[201] T. Simon‐Yarza , M. Giménez‐Marqués , R. Mrimi , A. Mielcarek , R. Gref , P. Horcajada , C. Serre , P. Couvreur , Angew. Chem. 2017, 129, 15771. 10.1002/anie.201707346 28960750

[202] F. Rahimi , S. Shahraki , M. R. Hajinezhad , S. Fathi‐Karkan , S. Mirinejad , S. Sargazi , M. Barani , R. Saravani , J. Drug. Deliv. Sci. Technol. 2024, 92, 105372.

[203] A. Ghosh , A. Ghosh , A. Bhattacharyya , R. Mitra , B. B. Das , A. Bhaumik , Dalton Trans. 2024, 53, 3010. 38265230 10.1039/d3dt03654e

[204] L. Gan , P. Ji , J. Zhang , H. Chen , Y. Yao , Z. Ren , Front. Bioeng. Biotechnol. 2023, 11, 1197484. 37324434 10.3389/fbioe.2023.1197484 PMC10267385

[205] P.‐H. Wu , P.‐F. Cheng , W. Kaveevivitchai , T.‐H. Chen , Colloids Surf., B 2023, 225, 113264. 10.1016/j.colsurfb.2023.113264 36921426

[206] X. Meng , J. Wu , Z. Hu , X. Zheng , Z. Wang , Chem. Eng. J. 2024, 484, 149313.

[207] X. Wang , Q. Chen , C. Lu , Molecules 2022, 27, 4247. 35807491 10.3390/molecules27134247 PMC9268424

[208] L. Wang , T. Wang , Y. Zhuo , S. Xu , H. Liu , X. Jiang , Z. Lu , X. Wang , H. Rao , D. Wu , Y. Wang , B. Feng , M. Sun , J. Colloid Interface Sci. 2024, 662, 962. 38382379 10.1016/j.jcis.2024.01.153

[209] X. Gong , J. Wang , L. Yang , L. Li , X. Gao , X. Sun , J. Bai , J. Liu , X. Pu , Y. Wang , Small 2023, 19, 2301497. 10.1002/smll.202301497 37086131

[210] R. Liu , J. Yang , Y. Du , X. Yu , Y. Liao , B. Wang , K. Yuan , M. Wang , Y. Yao , P. Yang , Adv. Mater. 2024, 36, 2310522. 10.1002/adma.202310522 38064417

[211] D. Zhang , J. Cao , H. Zhang , ACS Appl. Nano Mater. 2023, 6, 11361.

[212] G. Li , J. Zhang , S. Zhang , L. Teng , F. Sun , J. Controlled Release 2023, 362, 309. 10.1016/j.jconrel.2023.08.046 37634552

[213] Z. Chen , Y. Sun , J. Wang , X. Zhou , X. Kong , J. Meng , X. Zhang , ACS Nano 2023, 17, 9003. 37116070 10.1021/acsnano.2c10310

[214] Z. Zhao , Y. Wu , X. Liang , J. Liu , Y. Luo , Y. Zhang , T. Li , C. Liu , X. Luo , J. Chen , Y. Wang , S. Wang , T. Wu , S. Zhang , D. Yang , W. Li , J. Yan , Z. Ke , F. Luo , Adv. Sci. 2023, 10, 2303872. 10.1002/advs.202303872 PMC10602529 37661565

[215] H. Zhuang , P. Xue , S. Shao , X. Zeng , S. Yan , Int. J. Biol. Macromol. 2024, 258, 128952. 38143049 10.1016/j.ijbiomac.2023.128952

[216] J.‐J. Shen , S.‐J. Xue , Z.‐H. Mei , T.‐T. Li , H.‐F. Li , X.‐F. Zhuang , L.‐M. Pan , Heliyon 2024, 10, e28066. 38524612 10.1016/j.heliyon.2024.e28066 PMC10957435

[217] R. K. Alavijeh , K. Akhbari , ChemBioChem 2023, 24, 202300415. 10.1002/cbic.202300415 37553295

[218] Z. Lv , Y. Cao , D. Xue , H. Zhang , S. Zhou , N. Yin , W. Li , L. Jin , Y. Wang , H. Zhang , J. Mater. Chem. B 2023, 11, 1100. 36629834 10.1039/d2tb02273g

[219] C. Wu , D. Xu , M. Ge , J. Luo , L. Chen , Z. Chen , Y. You , Y. Zhu , H. Lin , J. Shi , Nano Today 2022, 46, 101574.

[220] B. Sun , X. Zheng , X. Zhang , H. Zhang , Y. Jiang , ACS Omega 2024, 9, 16676. 38617668 10.1021/acsomega.4c00658 PMC11007804

[221] U. Dhawan , C.‐L. Tseng , P.‐H. Wu , M.‐Y. Liao , H.‐Y. Wang , K. C.‐W. Wu , R.‐J. Chung , Nanomedicine 2023, 48, 102652. 36623714 10.1016/j.nano.2023.102652

[222] X. Wang , J. Luo , J. Wang , J. Cao , Y. Hong , Q. Wen , Y. Zeng , Z. Shi , G. Ma , T. Zhang , P. Huang , ACS Appl. Mater. Interfaces 2023, 15, 6442. 36700645 10.1021/acsami.2c19476

[223] L. He , Y. He , B. Chi , M. Xu , Q. Song , T. Yang , L. Li , J. Wang , Nanotechnology 2022, 34, 065101. 10.1088/1361-6528/aca0f8 36347034

[224] X. Kong , Z. He , Y. Zhang , Y. Fang , D. Liu , H. Wu , J. Ji , Y. Xi , L. Ye , X. Yang , G. Zhai , Chem. Eng. J. 2023, 468, 143729.

[225] M. Qian , G. Jiang , W. Guo , R. Huang , Nano Lett. 2024, 24, 3165. 38426438 10.1021/acs.nanolett.3c05146

[226] L.‐G. Li , X.‐X. Yang , H.‐Z. Xu , T.‐T. Yu , Q.‐R. Li , J. Hu , X.‐C. Peng , N. Han , X. Xu , N.‐N. Chen , X. Chen , J.‐M. Tang , T.‐F. Li , Adv. Healthcare Mater. 2023, 12, 2301561. 10.1002/adhm.202301561 37567571

[227] X. Li , E. Porcel , M. Menendez‐Miranda , J. Qiu , X. Yang , C. Serre , A. Pastor , D. Desmaële , S. Lacombe , R. Gref , ChemMedChem 2020, 15, 274. 31765517 10.1002/cmdc.201900596

[228] T. Jia , J. Du , J. Yang , Y. Li , T. Y. Ohulchanskyy , X. Fang , G. Chen , Adv. Funct. Mater. 2024, 34, 2307816.

[229] C. Hao , L. Huang , H. Zhang , L. Xu , M. Sun , H. Kuang , Q. Wang , C. Xu , Adv. Funct. Mater. 2024, 34, 2312795.

[230] Y. Huang , X. Li , Z. Zhang , L. Xiong , Y. Wang , Y. Wen , Cancers 2023, 15, 5043. 37894410 10.3390/cancers15205043 PMC10604985

[231] R. Ling , G. Chen , X. Tang , N. Liu , Y. Zhou , D. Chen , Discov Onc 2022, 13, 58. 10.1007/s12672-022-00521-1 PMC9263018 35798917

[232] T. Menter , A. Tzankov , S. Dirnhofer , Hematol Oncol 2021, 39, 3. 33105031 10.1002/hon.2821

[233] C. Zhang , L. Xin , J. Li , J. Cao , Y. Sun , X. Wang , J. Luo , Y. Zeng , Q. Li , Y. Zhang , T. Zhang , P. Huang , Adv. Healthcare Mater. 2022, 11, 2101946. 10.1002/adhm.202101946 34706160

[234] S. Sene , M. T. Marcos‐Almaraz , N. Menguy , J. Scola , J. Volatron , R. Rouland , J.‐M. Grenèche , S. Miraux , C. Menet , N. Guillou , F. Gazeau , C. Serre , P. Horcajada , N. Steunou , Chem 2017, 3, 303.

[235] F. Parsa , M. Setoodehkhah , S. M. Atyabi , Inorg. Chem. Commun. 2023, 155, 111056.

[236] M. Sun , L. Wang , Y. Zhuo , S. Xu , H. Liu , X. Jiang , Z. Lu , X. Wang , Y. Wang , G. Yue , B. Feng , H. Rao , D. Wu , Small 2024, 20, 2309593. 10.1002/smll.202309593 38126566

[237] E. Tavakoli , H. Sepehrmansourie , M. A. Zolfigol , A. Khazaei , A. Mohammadzadeh , E. Ghytasranjbar , M. A. As'Habi , Inorg. Chem. 2024, 63, 5805. 38511836 10.1021/acs.inorgchem.3c03742

[238] P. Zhang , J. Zhang , S. Bu , X. Huang , L. Shi , L. Cheng , J. Zhang , K. Wang , J. Phys. Chem. C 2024, 128, 2240.

[239] M. Beiranvand , S. Farhadi , A. Mohammadi‐Gholami , RSC Adv. 2023, 13, 13683. 37152578 10.1039/d3ra01180a PMC10157360

[240] M. Abbasian , M. Khayyatalimohammadi , Int. J. Biol. Macromol. 2023, 234, 123665. 36791936 10.1016/j.ijbiomac.2023.123665

[241] V. Agostoni , R. Anand , S. Monti , S. Hall , G. Maurin , P. Horcajada , C. Serre , K. Bouchemal , R. Gref , J. Mater. Chem. B 2013, 1, 4231. 32261018 10.1039/c3tb20653j

[242] M. Xu , X. Li , H. Zheng , J. Chen , X. Ye , T. Liu , Molecules 2022, 27, 2288. 35408686 10.3390/molecules27072288 PMC9000774

[243] D. Zhong , Y. Zuo , Y. Shi , P. Zhang , Y. Xu , B. Li , Chem. Eng. J. 2023, 460, 141837.

[244] S. Liao , Y. Yao , J. Duan , Q. Zhang , J. Du , S. Wu , F. Wang , C. Li , New J. Chem. 2024, 48, 544.

[245] O.‐E. Plastiras , P. Bouquet , C. Lecœur , J. Dhainaut , J.‐P. Dacquin , S. Royer , T. Loiseau , A. Goffard , C. Volkringer , Microporous Mesoporous Mater. 2024, 367, 112975.

[246] H. Zhang , Y. Zhang , Y. Li , M. Hu , Z. Rong , L. Meng , X. Zhang , L. Wen , X. Liang , Z. Chen , C. Liu , Advanced Therapeutics 2023, 6, 2300074.

[247] J. Wang , W. Ren , Y. Wang , D. Zhang , Y. Wang , P. Ju , K. Dou , Chem. Eng. J. 2023, 475, 146232.

[248] X. Qi , E. Grafskaia , Z. Yu , N. Shen , E. Fedina , A. Masyutin , M. Erokhina , M. Lepoitevin , V. Lazarev , N. Zigangirova , C. Serre , M. Durymanov , ACS Infect. Dis. 2023, 9, 1558. 37477515 10.1021/acsinfecdis.3c00131

[249] Z. Yang , C. Chen , B. Li , Y. Zheng , X. Liu , J. Shen , Y. Zhang , S. Wu , Chem. Eng. J. 2023, 451, 139127.

[250] X. Zhao , Y. Chen , R. Niu , Y. Tang , Y. Chen , H. Su , Z. Yang , X. Jing , H. Guan , R. Gao , L. Meng , Adv. Mater. 2023, 36, 2307839. 10.1002/adma.202307839 37812814

[251] Y. Shu , J. Wu , J. Zhang , X. Linghu , Y. Zhao , W. Liu , M. Di , D. Shan , X. Li , B. Wang , Appl. Surf. Sci. 2023, 639, 158249.

[252] J. Zhao , C. Lyu , R. Zhang , Y. Han , Y. Wu , X. Wu , J. Hazard. Mater. 2023, 442, 130018. 36155301 10.1016/j.jhazmat.2022.130018

[253] Y. Guo , Y. Li , R. Fan , A. Liu , Y. Chen , H. Zhong , Y. Liu , H. Chen , Z. Guo , Z. Liu , Nano Lett. 2023, 23, 8761. 37695577 10.1021/acs.nanolett.3c02857

[254] H. Liu , C. Xu , M. Meng , S. Li , S. Sheng , S. Zhang , W. Ni , H. Tian , Q. Wang , Acta Biomater. 2022, 144, 132. 35307591 10.1016/j.actbio.2022.03.023

[255] X. Cai , B. Liu , M. Pang , J. Lin , Dalton Trans. 2018, 47, 16329. 30403239 10.1039/c8dt02941e

[256] X. Li , N. Semiramoth , S. Hall , V. Tafani , J. Josse , F. Laurent , G. Salzano , D. Foulkes , P. Brodin , L. Majlessi , N.‐E. Ghermani , G. Maurin , P. Couvreur , C. Serre , M.‐F. Bernet‐Camard , J. Zhang , R. Gref , Part. Part. Syst. Charact. 2019, 36, 1800360.

[257] J. C. Díaz , B. Lozano‐Torres , M. Giménez‐Marqués , Chem. Mater. 2022, 34, 7817. 36117882 10.1021/acs.chemmater.2c01338 PMC9476658

[258] B. Yu , Q. Liu , J. Sun , X. Fu , Y. Zhang , X. Sun , Chem. Eng. J. 2024, 487, 150705.

[259] Z. Yu , X. Fu , T. Lucas , H. Zhao , C. Chen , I. Dubail , Y. Chen , G. Patriarche , J. Gateau , F. Gazeau , A. Jamet , M. Lepoitevin , C. Serre , ChemRxiv, 2024. 10.1002/adhm.202402418 PMC11694089 39460484

[260] A. C. McKinlay , J. F. Eubank , S. Wuttke , B. Xiao , P. S. Wheatley , P. Bazin , J.‐C. Lavalley , M. Daturi , A. Vimont , G. De Weireld , P. Horcajada , C. Serre , R. E. Morris , Chem. Mater. 2013, 25, 1592.

[261] J. F. Eubank , P. S. Wheatley , G. Lebars , A. C. McKinlay , H. Leclerc , P. Horcajada , M. Daturi , A. Vimont , R. E. Morris , C. Serre , APL Mater. 2014, 2, 124112.

[262] E. D. Bloch , W. L. Queen , S. Chavan , P. S. Wheatley , J. M. Zadrozny , R. Morris , C. M. Brown , C. Lamberti , S. Bordiga , J. R. Long , J. Am. Chem. Soc. 2015, 137, 3466. 25710124 10.1021/ja5132243

[263] E. D. Bloch , L. J. Murray , W. L. Queen , S. Chavan , S. N. Maximoff , J. P. Bigi , R. Krishna , V. K. Peterson , F. Grandjean , G. J. Long , B. Smit , S. Bordiga , C. M. Brown , J. R. Long , J. Am. Chem. Soc. 2011, 133, 14814. 21830751 10.1021/ja205976v

[264] R. V. Pinto , C.‐C. Cao , P. Lyu , I. Dovgaliuk , W. Shepard , E. Rivière , C.‐Y. Su , G. Maurin , F. Antunes , J. Pires , V. André , C. Henriques , A. Tissot , M. L. Pinto , C. Serre , Small 2024, 2405649. 39263810 10.1002/smll.202405649 PMC11600697

[265] Y. Mu , W. Li , X. Yang , J. Chen , Y. Weng , ACS Biomater. Sci. Eng. 2022, 8, 4777. 36256970 10.1021/acsbiomaterials.2c00959

[266] Y.‐H. Hong , M. Narwane , L. Y.‐M. Liu , Y.‐D. Huang , C.‐W. Chung , Y.‐H. Chen , B.‐W. Liao , Y.‐H. Chang , C.‐R. Wu , H.‐C. Huang , I.‐J. Hsu , L.‐Y. Cheng , L.‐Y. Wu , Y.‐L. Chueh , Y. Chen , C.‐H. Lin , T.‐T. Lu , ACS Appl. Mater. Interfaces 2022, 14, 3849. 35019259 10.1021/acsami.1c21409

[267] C.‐W. Chung , B.‐W. Liao , S.‐W. Huang , S.‐J. Chiou , C.‐H. Chang , S.‐J. Lin , B.‐H. Chen , W.‐L. Liu , S.‐H. Hu , Y.‐C. Chuang , C.‐H. Lin , I.‐J. Hsu , C.‐M. Cheng , C.‐C. Huang , T.‐T. Lu , ACS Appl. Mater. Interfaces 2022, 14, 6343. 35080366 10.1021/acsami.1c20802

[268] H. B. Ji , S.‐N. Kim , C. R. Kim , C. H. Min , J. H. Han , M. J. Kim , C. Lee , Y. B. Choy , Biomater. Adv. 2023, 145, 213268. 36580769 10.1016/j.bioadv.2022.213268

[269] J. Yao , Y. Liu , J. Wang , Q. Jiang , D. She , H. Guo , N. Sun , Z. Pang , C. Deng , W. Yang , S. Shen , Biomaterials 2019, 195, 51. 30610993 10.1016/j.biomaterials.2018.12.029

[270] H. Zhao , S. Becharef , E. Dumas , F. Carn , G. Patriarche , S. Mura , F. Gazeau , C. Serre , N. Steunou , Nanoscale 2024, 16, 12037. 38809107 10.1039/d3nr06685a

[271] Q. Li , X. Ding , Z. Chang , X. Fan , J. Pan , Y. Yang , X. Li , W. Jiang , K. Fan , Adv. Healthcare Mater. 2024, 13, 2303454. 10.1002/adhm.202303454 38031989

